# Dystrophins DP71 and DP427 determine cell viability during proliferation and myofibre differentiation

**DOI:** 10.1038/s41419-026-08725-x

**Published:** 2026-04-11

**Authors:** Sylwia Szwec, Alicja Durska, Paulina Kościelniak-Wawro, Jeffrey S. Chamberlain, Oleksandr Ostrovskyy, Karolina Dominiak, Solmaz Karimi, Patryk Konieczny

**Affiliations:** 1 Institute of Human Biology and Evolution, Faculty of Biology, Adam Mickiewicz University, Poznań, Poland; 2 Department of Neurology, University of Washington School of Medicine, Seattle, WA USA; 3 Senator Paul D. Wellstone Muscular Dystrophy Specialized Research Center, University of Washington School of Medicine, Seattle, WA USA; 4 Department of Biochemistry, University of Washington School of Medicine, Seattle, WA USA; 5 Department of Medicine, University of Washington School of Medicine, Seattle, WA USA

**Keywords:** Cell proliferation, Differentiation, Muscle stem cells, Mechanisms of disease

## Abstract

Duchenne muscular dystrophy (DMD) is an X-linked disease caused by mutations in the dystrophin gene. DMD manifests with progressive skeletal muscle wasting, cardiac dysfunction, and cognitive and neuropsychiatric symptoms. As the disease progresses, affected boys lose their ability to walk and die prematurely. All DMD patients lack the full-length DP427 dystrophin, whereas approximately 10% lose all dystrophins, including DP71, synthesized in various cell types, including myoblasts. There is evidence that the cognitive symptoms of DMD patients vary depending on the location of the mutation site and the number of missing dystrophins, and there is some indication that this may also be the case in skeletal muscle. We therefore investigated the roles of DP427 and DP71 during cell proliferation and fibre differentiation. We included paralogous utrophins in our analyses, particularly the UP395 isoform, owing to its presence in muscle and its partial ability to compensate for the lack of DP427. We demonstrated that DP71 and DP427 play important roles in cell viability during cell proliferation and fibre differentiation, respectively. Despite their different expression patterns, the absence of DP71 and DP427 resulted in a similar phenotype, including increased membrane permeability, mitochondrial aggregation, elevated ROS, and increased cyto- and genotoxicity, but induced substantially different transcriptome programs associated with impaired cell proliferation and structural reorganization of myotubes, respectively. The phenotype occurred independently of myofibre contraction, dysfunction of neuromuscular or myotendinous junctions, or other cell types. We further showed that the utrophin UP395 can partially compensate not only for DP427 but also for DP71. These results explain the observed differences in disease severity among DMD patients and suggest that individuals deficient in both DP71 and DP427 may require a different therapeutic approach than patients deficient in only DP427.

## Introduction

In the mid-19th century, the French neurologist Guillaume B.A. Duchenne de Boulogne identified a distinct type of muscular dystrophy that predominantly affected young boys. The disease named after him, Duchenne muscular dystrophy (DMD), affects approximately 1 in 5000 live male births and is not only one of the most severe forms of dystrophy but also the most common neuromuscular genetic disorder [1]. DMD manifests predominantly in progressive skeletal muscle wasting, respiratory difficulties, and cardiac dysfunction; however, it also affects smooth muscles, the nervous system and bones [2]. As the disease progresses, the affected boys lose their ambulation by the age of 13 and die prematurely usually in their 30 s, from heart failure or respiratory failure.

In the late 1980s, the dystrophin (*DMD*) gene was cloned, and DMD was characterised as a loss-of-function disease in which the full-length 427 kDa dystrophin (DP427) protein was absent from muscle samples [3]. Moreover, mildly affected Becker muscular dystrophy (BMD) patients also suffer from mutations in the *DMD* gene, but unlike DMD patients, their biopsies retain some dystrophin reactivity. Indeed, consistent with the reading frame hypothesis, DMD patients typically have out-of-frame mutations that lead to premature stop codons and loss of dystrophin mRNA, whereas in BMD patients, in-frame mutations result in translation of shorter and partially functional dystrophins, a property utilised for developing drugs for boys with DMD [2]. Available treatments include antisense oligonucleotides and a gene therapy treatment designed to generate partially functional truncated/micro-dystrophins similar to those in BMD.

The phenotype of DMD patients varies depending on the site of mutation [2]. All DMD patients have no functional DP427; however, most of them lack one or more shorter dystrophins. For example, among the 181 patients tested by Yamamoto et al., approximately 75% of individuals had mutations predicted to cancel the translation of DP260; 64% of DP260 and DP140; 12% of DP260, DP140 and DP116; and 7% of DP260, DP140, DP116 and DP71 [4]. Dystrophin isoforms are synthesized from 7 promoters within the *DMD* gene in a tissue- and time-dependent manner [5], and there is also an alternative polyadenylation site, which results in the generation of the shortest dystrophin, DP40. Another level of dystrophin diversity is based on the alternative splicing of dystrophin transcripts, which can determine the cellular localization and function of the generated proteins [6, 7]. One of the three full-length dystrophin proteins, DP427M, is translated in muscles, particularly in skeletal and cardiac muscles, where it assembles the membrane-localized dystrophin-glycoprotein complex (DGC), which is crucial for transmitting forces during contraction and signal transduction. The remaining two long isoforms, DP427C and DP427P, are present in neurons of the cortex and cerebral Purkinje cells, respectively. The shorter dystrophin variant DP260 is present in the retina; DP140 and DP40 are typical of the brain; DP116 occurs in Schwann cells, striated muscle and fibroblasts; and DP71 is ubiquitously synthesized throughout the body [2, 5]. While DP427 can interact with many proteins, including actin, microtubules, nNOS, syntrophins or β-dystroglycan, shorter isoforms lose some of their binding capacity but retain the ability to assemble the DGC [8–11].

The dystrophin paralogue utrophin has gained significant attention in the field of DMD therapy because of its increased synthesis in dystrophic myofibres and its potential to partially compensate for the lack of dystrophin [12] without inducing an immune response [8, 13, 14]. Current results indicate that, similar to the *DMD* gene, the utrophin (*UTRN*) gene is also transcribed from various promoters, giving rise to full-length utrophin (UP395) as well as shorter UP140, UP113 and UP71 isoforms, the synthesis of which is often complementary to that of dystrophin. DP71, DP116, DP427 and UP395 are reportedly present at various stages of myofibre differentiation [4, 15–18]. DP71 and UP395 are considered foetal isoforms, whereas DP427 is regarded as the adult isoform, the absence of which leads to fibre degeneration [8]. Nevertheless, the role of these dystrophin/utrophin isoforms at different stages of fibre differentiation is unclear and may be overshadowed by their mutual and compensatory functions.

Despite differences in mechanical properties and inability to bind proteins such as nNOS, UP395 interacts with actin filaments and assembles the utrophin-glycoprotein complex (UGC) [8, 19]. This dual activity is sufficient to partially prevent contraction-induced fibre damage and ameliorate muscular dystrophy in animal models of DMD, as also demonstrated in gene therapy treatments using recombinant AAV vectors carrying shortened dystrophin and utrophin sequences encoding micro-dystrophin and micro-utrophin proteins [13, 14, 20]. In contrast, DP71 synthesis in myofibres from a transgene in mice lacking DP427 (*mdx* mice) was shown not to be beneficial, even though the DGC was assembled [9, 10]. However, other data indicate that DP71 might have important functions during cell proliferation [6, 7], whereas the absence of DP427 is thought to result in myofibre death because of altered mechanical properties of the sarcolemma, increased reactive oxygen species generation, and overload of mitochondria with calcium [2, 21]. There are also data showing that the additional absence of utrophin in *mdx* (*mdx*/*Utrn*^−/−^; dko) mice worsens the mitochondrial phenotype in the muscle [22], possibly due to the lack of compensatory functions.

In the present work, we aimed to decipher the role of dystrophin and utrophin during cell proliferation and myofibre differentiation/regeneration. In particular, we aimed to (1) characterise the expression patterns of the *DMD* and *UTRN* genes at the RNA and protein levels, (2) assess compensatory expression of the *UTRN* gene in the absence of dystrophin, (3) evaluate the distinct and compensatory functions of various dystrophin and utrophin isoforms and (4) delineate the order in which pathologies occur in dystrophin and dystrophin/utrophin deficiency during myofibre differentiation.

## Methods

### Mice

Age- and sex-matched wt (C57/Bl6) and *mdx* ^*4cv*^/*Utrn*^−/−^ (dko [23]) 8–10-week-old mice were used throughout the study.

### Notexin (NTX) and BrdU injections

The EDL muscles were surgically exposed and injected with 100 μl of 1.0 μg/ml NTX as described previously [24]. A total of 750 μg of BrdU (250 μl; Sigma Aldrich) was administered subcutaneously.

### Cryosections and single-fibre cultures

Dissected muscles were frozen in cryomolds in liquid nitrogen-cooled isopentane and sectioned. Single muscle fibres were isolated from collagenase-treated EDL muscles, plated in the well of a Matrigel-coated chamber slide (NUNC, 177445), and incubated as previously described [25]. The media were supplemented with 1% penicillin/streptomycin solution.

### Cells and cocultures

The C2C12 (CRL-1772) and HeLa cells were obtained from ATCC, whereas the HSkM (A12555) and HEK293 cells were obtained from Thermo Fisher, and tested for mycoplasma contamination via PCR. C2C12, HEK293, and HeLa cells were cultured in Dulbecco’s modified Eagle’s medium (DMEM; Capricorn Scientific, DMEM-HA or Biowest, LO102-500) supplemented with 10% foetal bovine serum (FBS; Genos, FBS-HI-12A) and 1% antibiotic antimycotic reagent (AA; Sigma Aldrich, A5955). HSkM cells were cultured in Nutrient Mixture F-10 Ham Medium (Thermo Fisher, 11550043) supplemented with 20% FBS, 10 µg/µl epidermal growth factor (EGF; Sigma Aldrich, E9644), 1 mg/µl dexamethasone (Sigma Aldrich, D4902), and 1% AA. For differentiation, C2C12 and HSkM cells were seeded to high confluence (>80%) and then cultured in DMEM-based starvation medium (Capricorn Scientific, DMEM-HA) supplemented with 2% horse serum (Sigma Aldrich, H1138) and 1% AA. C2C12 and HSkM cells were seeded on cell culture plates covered with 0.1% gelatin (Sigma Aldrich, G9391). For cocultures, HSkM cells were seeded on 0.1% gelatin-coated 24-well plates in the proliferation medium. When the HSkM cells reached 60% confluence, the cells were washed with PBS, and differentiation was induced by switching to starvation medium with 6 × 10^4^ suspended HeLa cells (wt/ko). The cocultures were then maintained for 5 days. All the cells were grown at 37 °C in a humidified incubator containing 5% CO_2_.

### siRNA transfection

Cell transfection with Silence Select siRNA (Thermo Fisher) was performed with Lipofectamine RNAiMAX Transfection Reagent (Thermo Fisher, 13778150) according to the manufacturer’s protocol. The final concentration of each siRNA was 25 nM (for double siRNA transfection, the final concentration of negative control siRNA was 50 nM). For the experiments involving proliferating cells, the material was collected 48 h after transfection. For differentiation, siRNA was added simultaneously with the induction of differentiation. For C2C12 cells, siRNA transfection was performed twice, with an additional transfection on day 2. The siRNAs used in this study are listed in Table S1.

### *DMD* ko cell lines

*DMD* ko cell lines were derived from HeLa and HEK293 cells. The cells were seeded at 30% confluency in 24-well plates and transfected twice, 24 and 48 h after passage. For each transfection, 2.5 µl of Lipofectamine CRISPRMAX Cas9 Transfection Reagent (Thermo Fisher, CMAX00008) was used per well, along with 7.5 pmol of TrueGuide sgRNA (Thermo Fisher, A35534) and 1.25 µg of TrueCut Cas9 Protein v2 (Thermo Fisher, A36498). The cells were collected 96 h after seeding, with half of the material used to evaluate knockout efficiency and the other half used for single-colony expansion. Single-cell clones for further validation and banking were obtained via the limiting dilution cloning method. Clones were validated by Sanger sequencing, PCR, and Western blotting. The sgRNAs used in this study are listed in Supplementary Table S1.

### Immunofluorescence, histochemical staining, and imaging

#### Tissue sections, single fibres, and single fibre-derived cultures

Cultures derived from single fibres were immunostained according to the published protocol [26]. SDH, COX, and costameric staining were performed as previously described [27]. To visualise BrdU and desmin, cryosections were fixed with acetone at −20 °C for 10 min, denatured in 2.5 M HCl for 30 min, and permeabilized with 0.25% Triton X-100 for 5 min before the M.O.M. Basic Kit (Vector Laboratories, BMK-2202) was used. For single PAX7 immunostaining, cryosections and freshly isolated fibres were subjected to 4% formaldehyde (FA) fixation and antigen retrieval with citrate buffer at 100 °C for 15 min. Cryosections were then permeabilized with 0.25% Triton X-100/phosphate buffered saline (PBS) for 10 min, blocked, and immunostained via the M.O.M. kit. Fixed fibres were resuspended and blocked in 5% normal goat serum and 0.2% Triton X-100/PBS for 1 h and immunolabelled with the PAX7 antibody, which was added directly to the blocking solution O/N at 4 °C on a rotating mixer. For other single and double immunofluorescent labelling, cryosections were fixed with 2% FA and processed without antigen retrieval. The samples were incubated with the secondary antibody for 45 min at RT. After secondary antibody incubation, DAPI (Sigma Aldrich, D9542) was used to visualise the nuclei. Immunolabelled cell and tissue samples were mounted with Mowiol (Calbiochem, 475904-M). The antibodies used in this study are listed in Supplementary Table S2.

The images were captured with a Nikon Eclipse E1000 scope, QImaging QICAM Fast 1394 camera, Nikon Plan 10×/0.25 and Plan Apochromat 20×/0.75 objectives, and QImaging QCapture software (version 2.95); an Olympus ZX16 scope, DP71 camera, SDF PLAPO 2xPFC and 1×PF objectives, and DP Controller software (version 3.1); and a Carl Zeiss 510 META confocal scope, Plan Apochromat 20×/0.75 and Plan Apochromat 63×/1.4 objectives, and LSM510 software (version 4.2). Composite images were acquired via the Syncroscan stage attached to the Nikon Eclipse E1000 scope and Syncroscan software (4.05; Syncroscopy). Fibre areas and the number of nuclei in fibre-derived cultures were assessed via ImageJ software. After the images were converted to 8-bit grayscale, the background signal was removed via the Subtract Background function in the Process menu. The signals were then identified by adjusting the threshold (Image > Adjust > Threshold), and the number of nuclei was assessed via the Analyse > Analyse Particles option, and the myosin-positive areas were quantified via the Analyse > Measure option. To ensure consistency, all the images were processed via identical processing steps and settings.

#### Cells and cocultures

The cells were plated on 0.1% gelatin-coated coverslips, and the cocultures grown on 0.1% gelatin-coated plates were fixed in 2% FA for 15 min and then washed 3 times with PBS. The cells were then permeabilized with 0.2% Triton X-100 in PBS for 10 min and blocked with 5% normal donkey serum (NDS, Jackson ImmunoResearch, 017-000-121) and 0.2% Triton X-100 in PBS for 30 min. The samples were incubated with primary antibodies diluted in 2% NDS and 0.1% Triton X-100 in PBS for 2 h at RT. The cells were then washed 3 times with PBS, incubated with secondary antibodies diluted in 2% NDS, 0.1% Triton X-100 in PBS for 1 h at RT, and washed 3 times again with PBS. Coverslips were mounted on microscopic slides with ProLong Diamond Antifade Mountant (Thermo Fisher, P36965) and examined via a Leica Stellaris 8 confocal system (Leica, Germany) equipped with a white laser, HyD S detectors, HC PL APO CS2 20×/0.75, HC PL APO CS2 40×/1.10 and HC PL APO CS2 63×/1.40 OIL objectives. The plates with labelled myotubes and cells were also visualised with a Leica DM IL-Led (Leica, Germany) microscope with N Plan Fluor 10×/0.30 and N Plan Fluor 20×/0.40 objectives. For cocultures, four images from representative myosin-stained areas were taken for each well, and the images were analysed via ImageJ software as described above. All images from each well were added and divided by the number of images to obtain a value for one biological replicate.

All the images were processed in ImageJ or CorelDRAW (brightness, contrast) and assembled via CorelDRAW. The antibodies used in this study are listed in Supplementary Table S2.

### Immunoblotting

#### Muscles

Muscle protein extracts were prepared as described elsewhere [27]. The samples were diluted in NuPAGE lithium dodecyl sulfate (LDS) Sample Buffer (4×) with NuPAGE Reducing Agent (10×; Invitrogen). Proteins were separated via NuPAGE 4‒12% Bis‒Tris gels (Invitrogen) and electrophoretically transferred to polyvinylidene fluoride (PVDF) membranes at 100 V for 2 h at RT or at 30V, O/N, and 4 °C, or stained with Coomassie blue. The membranes were blocked with 5% BSA or 5% nonfat milk in tris-buffered saline (TBS) with 0.1% Tween (TTBS) for 1 h at RT. The primary antibodies were diluted in blocking solution and incubated with the membranes either for either 2 h at RT or O/N at 4 °C. The membranes were washed three times (10‒15 min each) with TTBS and probed with horseradish peroxidase-conjugated antibodies for 1 h at RT. After further washing, detection was performed with ECL/ECL plus (Pierce), and the samples were scanned with a Storm 860 imaging system (Amersham Biosciences). Densitometric analysis was performed with ImageJ. Band intensities were normalized to the total protein content, which was evaluated densitometrically from Coomassie blue-stained gels.

#### Cells

Protein extraction from the cell lines was performed with CelLytic M (Sigma Aldrich, C2978) supplemented with cOmplete Mini Protease Inhibitor Cocktail (Sigma Aldrich, 11836153001). After the protein concentration was measured with a Pierce BCA protein assay kit (Thermo Fisher, 23227), the extracts were mixed with sample buffer (4× sample buffer: 0.8 g of SDS, 2 ml of 1 M Tris (pH = 8.0), 4 ml of glycerol, 1 ml of 0.5 M EDTA (pH=8.0), 0.4 ml of β-mercaptoethanol, and 8 mg of Brilliant Blue) and then denatured for 5 min at 95 °C. Western blotting was performed by loading 20 μg of protein extracts per lane onto 6‒12% sodium dodecyl sulfate‒polyacrylamide gels (SDS‒PAGE). Proteins were then transferred to methanol-activated PVDF membranes (0.45 µm) (Thermo Fisher, 88518) for 16 h at 25 V at 4 °C via a wet blotting system (Bio-Rad). To improve the transfer of large proteins, such as DP427 and UP395, the transfer buffer was supplemented with 0.03% SDS. The membranes were blocked with 5% nonfat milk in TTBS for 1 h at RT and incubated with primary antibody in blocking buffer O/N at 4 °C or 2 h at RT. After incubation with primary antibodies, the membranes were washed 3 times for 20 min with TTBS and then incubated with secondary antibodies in blocking buffer for 1 h at RT, and the washing procedure was repeated. Protein detection with the Clarity Western ECL Substrate (Bio-Rad, 1705060) was performed on a ChemiDoc Imaging System (Bio-Rad). SimplyBlue SafeStain (Coomassie G-250; Thermo Fisher, LC6060)-stained gels and Ponceau S Staining Solution (Thermo Fisher, A40000278)-labelled membranes were used as loading controls.

The antibodies used in this study are listed in Supplementary Table S2. The original Western blot images are provided in the Supplementary files.

### RT-qPCR

Total RNA was extracted with a Total RNA Zol-Out kit (A&A Biotechnology, 030-100), and 1 µg of total RNA was reverse transcribed with a RevertAid First Strand cDNA Synthesis Kit (Thermo Fisher, EP0442). RT-qPCR was performed with Power SYBR™ Green PCR Master Mix (Thermo Fisher, 4368708) with 20 ng of cDNA and primers at a final concentration of 1.5 µM. All RT-qPCR data were analysed in Microsoft Excel via the 2^−ΔΔCt^ method. The Ct values of the analysed target genes were normalized against those of the reference housekeeping genes *RPLP0* or *Csnk2a2*. The primers used in this study are listed in Supplementary Table S1. The raw RT-qPCR data are provided in the Supplementary files.

### DNA isolation and mitochondrial DNA content

DNA was isolated with the GeneJET Genomic DNA Purification Kit (Thermo Fisher, K0721). The mitochondrial content was determined from the ratio of mitochondrial DNA (mtDNA, *ND1* (human)/*Nd1* (mouse) gene) to genomic DNA (gDNA, *SERPINA* (human)/*Hk2* (mouse) gene) via qPCR analysis (the primers are listed in Supplementary Table S1).

### Cell viability and proliferation

Cell viability/proliferation was determined via the RealTime-Glo™ MT Cell Viability Assay (Promega, G9711) according to the manufacturer’s instructions. Briefly, HeLa and HEK293 cells were plated on a Nunc MicroWell 96-well plate (Thermo Fisher) at a density of 1000 cells per well, and HSkM cells were plated at 5000 cells per well. The cells were cultured O/N in 100 µL of complete medium per well. For experiments involving siRNA, 40 µL of medium was removed from each well, and 10 µL of OPTI-MEM containing the siRNA mixture was added. Then, 30 µL of complete medium containing 0.1 µL of MT Cell Viability Substrate and 0.1 µL of Nanoluc Enzyme was added to each well. For the experiments without siRNA, 40 µL of the mixture was prepared. The first luminescence measurement was performed after a 1 h incubation at 37 °C and then every 24 h using a Tecan SPARK plate reader to assess cell viability and proliferation over time.

### Mitochondrial content and activity

The cells were plated on Greiner CELLSTAR 96-well plates (Sigma Aldrich), and measurements were taken via a Tecan SPARK plate reader. The mitochondria were stained with MitoTracker Green FM (Thermo Fisher, M7514), a carbocyanine-based dye that passively diffuses across the plasma membrane and accumulates in active mitochondria. The reagent was used according to the manufacturer’s instructions, and the intracellular localization of the mitochondria was confirmed via fluorescence microscopy. Mitochondrial membrane potential images were assessed with Image-iT TMRM Reagent (Thermo Fisher; I34361), a cell-permeant dye that accumulates in active mitochondria with intact membrane potential. The reagent was also used according to the manufacturer’s instructions. Briefly, the medium was removed from the cells and replaced with a staining solution prepared by diluting the reagent (1000× stock, 100 µM in dimethyl sulfoxide (DMSO)) 1:1000 in cell growth medium to a final concentration of 0.1 µM. After 30 min of incubation at 37 °C, the cells were washed with PBS prior to analysis. Hoechst was added to the cells (1 µg/mL) at the same time as the MitoTracker and Image-iT TMRM reagents were added to normalize the signals to the number of cells. The excitation/emission wavelengths were adjusted experimentally to avoid signal crossover. The following excitation/emission wavelengths were used: MitoTracker, 485/525; Image-iT TMRM, 538/584; and Hoechst, 350/461.

### Oxidative stress, cytotoxicity, genotoxicity, apoptosis, calcium levels, and plasma membrane staining

The cells were plated on Greiner CELLSTAR 96-well plates (Sigma Aldrich), and readings were obtained via a Tecan SPARK plate reader. Oxidative stress was assessed via the use of CellROX Green Reagent and CellROX Deep Red Reagent (Thermo Fisher Scientific, C10444 and C10422, respectively) according to the manufacturers’ instructions. These dyes indicate the presence of ROS by emitting bright fluorescence. The CellROX Deep Red dye localizes in the cytoplasm, whereas the CellROX Green reagent, once oxidized, binds to DNA, localizing primarily in the nucleus and mitochondria. The intracellular localization of ROS was confirmed by fluorescence microscopy. Cytotoxicity and genotoxicity were assessed via the HCS DNA Damage Kit (Thermo Fisher Scientific, H10292), which is composed of two separate reagents. Both reagents were used according to the manufacturer’s instructions. Cytotoxicity was evaluated via the use of Image-iT DEAD Green, an impermeable dye in healthy cells that becomes permeant upon loss of plasma membrane integrity, thereby labelling cells with membrane breaks, whereas DNA damage was detected via an antibody against phosphorylated histone H2AX (Ser139), a marker induced in response to double-strand break formation. Cell viability, cytotoxicity, and apoptosis were measured in the same well via the ApoTox-Glo Triplex Assay (Promega, G6320) according to the manufacturer’s instructions. Viability and cytotoxicity were assessed with a nonlytic reagent containing two peptide substrates: a cell-permeant fluorogenic substrate (GF-AFC) cleaved by live-cell proteases in intact cells (viability) and a cell-impermeant substrate (bis-AAF-R110) that detects protease activity released from cells with compromised membranes (cytotoxicity). The results are shown as a ratio of these measurements, showing a cell-number independent readout. Apoptosis was evaluated by a second reagent containing a luminogenic DEVD peptide substrate for caspase-3/7 and Ultra-Glo luciferase; caspase-3/7 activity releases luciferin, producing a luminescent signal proportional to apoptotic activity. Intracellular calcium levels were measured with Fluo-8 AM (AAT Bioquest, 21080) according to the manufacturer’s instructions. For plasma membrane staining, a fresh working solution of CellMask Deep Red plasma membrane stain (Thermo Fischer, C10046) was prepared in growth medium by diluting the supplied 1000× stock solution and incubating it for 5–10 min at 37 °C before fluorescence measurement. Hoechst was added to the cells (1 µg/mL) to normalize the signals to the number of cells. The excitation/emission wavelengths were adjusted experimentally to avoid signal crossover. The following excitation/emission wavelengths were used: Cell Rox Deep Red, 640/690; CellMask Deep Red, 649/666; genotoxicity (HCS Damage Kit), 540/590; Cell Rox Green, 485/550; cytotoxicity (HCS Damage Kit)/Fluo-8 AM, 485/530; cytotoxicity (ApoTox-Glo) 480/525; viability (ApoTox-Glo), 400/505; Hoechst, 350/461; and apoptosis (ApoTox-Glo), luminescence.

### Statistical analyses for non-RNA-seq data

For smaller group sizes (*n* < 30), statistical significance between samples was calculated via a two-sided and unpaired *t*-test for equal or unequal variances. The *F*-test was used as a prerequisite to determine whether variances were equal before selecting the *t*-test. The studies on mice involved comparing specific genotypes, therefore no randomization method and no blinding were used to assign animals to specific groups. The number of EDL muscles, EDL fibers, and EDL fibre-derived cultures obtained from mice is indicated in the figures. For functional assays, at least five biological replicates per group were typically used, as indicated in the figures. For immunoblot and siRNA efficacy studies, fewer samples were used due to the expected large effect sizes. For RT-qPCR data, one biological replicate was typically obtained from averaging three technical replicates. Technical replicates far from the mean values were considered pipetting errors and removed from the analyses. The *z*-test was performed if the number of samples was greater than 30 per group. The Shapiro–Wilk test was used to assess the parametric distribution of samples after excluding outliers. Calculations for *F*-tests, *t*-tests, and *z*-tests were performed in Excel. Results are presented as means with standard deviations, as indicated in the figure legends. Sample sizes (biological replicates) are denoted as n and are given in the figures. Statistical significance is indicated as ^*^P < 0.05; ^**^P < 0.01; and ^***^P < 0.001.

### RNA sequencing and data analyses

#### Sample preparation

HeLa and HSkM cells were transfected with siRNA and cultured in nine biological replicates for each siRNA. For RNA isolation, every three biological replicates were combined to create a single pooled replicate as one biological repeat. Total RNA was extracted via Total RNA Zol-Out™ and RNA Clean & Concentrator-5 with DNase I (Zymo Research, R1014). The quality of the total RNA was evaluated via a TapeStation 2200 with an RNA ScreenTape assay kit (Agilent Technologies). Only samples with an RNA integrity number (RIN) ≥ 8 were used for further analyses. Following rRNA depletion and cDNA library preparation, RNA-Seq libraries were sequenced on a NovaSeq platform (Illumina) to generate 150-bp paired-end reads (PE150).

#### rRNA depletion and cDNA library preparation

rRNA depletion was performed via the RiboCop rRNA Depletion Kit V2 (Lexogen, 183–184) according to the manufacturer’s protocol. The quality and quantity of depleted RNA were evaluated via the TapeStation 2200 with the High Sensitivity RNA ScreenTape assay kit (Agilent). cDNA libraries were prepared via the RNA-Seq V2 Library Prep Kit with UDIs (Lexogen, UDI12A_0001-0096) following the Long Insert Sizes (RTL) protocol. Libraries were amplified in 11 cycles. The concentration and size range of the cDNA libraries were determined via the TapeStation 2200 with the High Sensitivity DNA ScreenTape assay kit (Agilent). The resulting libraries were pooled, and the final concentration and size distribution were verified via the TapeStation 2200 (Agilent).

#### RNA-sequencing and sequencing data analyses

The RNA-Seq libraries were sequenced on a NovaSeq platform (Illumina) to generate 150-bp paired-end reads (PE150). Adaptor removal was performed via Cutadapt (https://cutadapt.readthedocs.io/en/stable/) followed by alignment to the reference genome via HISAT2 (https://daehwankimlab.github.io/hisat2/) [28]. The GRCh38.p14 reference genome was utilised (https://www.ncbi.nlm.nih.gov/datasets/genome/GCF_000001405.40/). The number of paired reads mapped to individual genes was subsequently quantified via HTseq (https://HTSeq.readthedocs.io/en/release_0.10.0/index.html), with strand specificity considered (--stranded=reverse). Gene annotations were assigned on the basis of a gene description file available from the DRYAD database (https://datadryad.org/stash) containing InterProScan results [29, 30].

The level of gene expression was calculated via the R package DESeq2. For gene expression analysis, a negative binomial distribution was applied, utilising the gene-specific mean and dispersion parameters. The changes in transcript counts were tested via the Wald method, which allowed us to determine the statistical significance of the expression changes in the analysed genes. The obtained *p*-values were corrected for multiple testing via the Benjamini‒Hochberg method (FDR ≤ 0.05). Only statistically significant genes with FDRs ≤ 0.05 were included in the comparative analyses of differentially expressed genes (DEGs). The results are presented as log2_Estimated_FoldChange (log2FC), which represents the logarithm base 2 of the fold change in transcript abundance between the experimental sample and the control sample.

KEGG (Kyoto Encyclopedia of Genes and Genomes) enrichment analysis was performed by retrieving gene identifiers matching the KEGG database. For this purpose, BLASTP was used to search peptide sequences from the *Homo sapiens* genome that corresponded to genes in the KEGG database (https://www.kegg.jp/kegg/kegg2.html) [31]. Only BLASTP results with identity scores above 90% were included in the KEGG analysis. The final results were processed in the R environment using the DESeq2 package [32]. Biological pathways from the KEGG database for statistically significant genes with EntrezId identifiers in the analysed comparisons were determined via the clusterProfiler package [33, 34]. This package has the advantage of using the latest version of the KEGG database. For KEGG analysis, only genes identified as significantly differentially expressed (FDR ≤ 0.05, p.adjust) were included. Visualisation of KEGG pathway data was performed via Pathview (https://bioconductor.org/packages/release/bioc/html/pathview.html) [35].

Gene Ontology (GO) enrichment analysis was conducted on significantly DEGs (FDRs ≤ 0.05) via the topGO package in R. Enrichment analysis was performed independently for the three GO categories: molecular function (MF), biological process (BP), and cellular component (CC). Two statistical methods available in topGO - classic and elim - were applied to calculate Fisher’s exact test *p*-values. The classic method performs standard GO term enrichment testing independent of the GO hierarchy, whereas the elim method prioritizes specific GO terms by accounting for hierarchical dependencies among terms, thereby reducing redundancy and focusing on biologically informative results (https://bioconductor.org/packages/release/bioc/html/topGO.html) [36]. The results were visualised as bubble plots, where the x-axis represents the gene ratio (proportion of DEGs per term), bubble size reflects the gene count per term, and colour intensity indicates -log₁₀(p-value), with red and green representing high and moderate statistical significance, respectively.

The same samples used for RNA-seq analysis (*DMD*ko+si*UTRN* HeLa and si*DMD*/*UTRN* HSkM, each relative to the corresponding siCtrl sample) were used for validation, covering 8 genes per cell type. RT-qPCR data were converted to log₂FC values, and Pearson’s correlation coefficient between the RNA-seq and qPCR results was calculated.

## Results

### Loss of dystrophin Dp427 and utrophin in skeletal muscles leads to proteomic changes and alterations in the organization of the mitochondrial network

To address the functions of DP427 in skeletal muscles, we used wild-type (wt) and severely affected DP427- and utrophin-deficient mice (*mdx* ^*4cv*^/*Utrn*^−/−^, dko mice). Dko mice represent the muscle wasting phenotype of DMD patients much better than mildly affected *mdx* mice because they are much smaller, their movements are greatly restricted, and they live for only up to 20 weeks [37]. This phenotypic severity is explained by the lack of compensatory synthesis of UP395, which can connect actin filaments to the extracellular matrix via the assembled UGC and partially prevents the contraction-induced degeneration of myofibres [19].

**Fig. 1:**
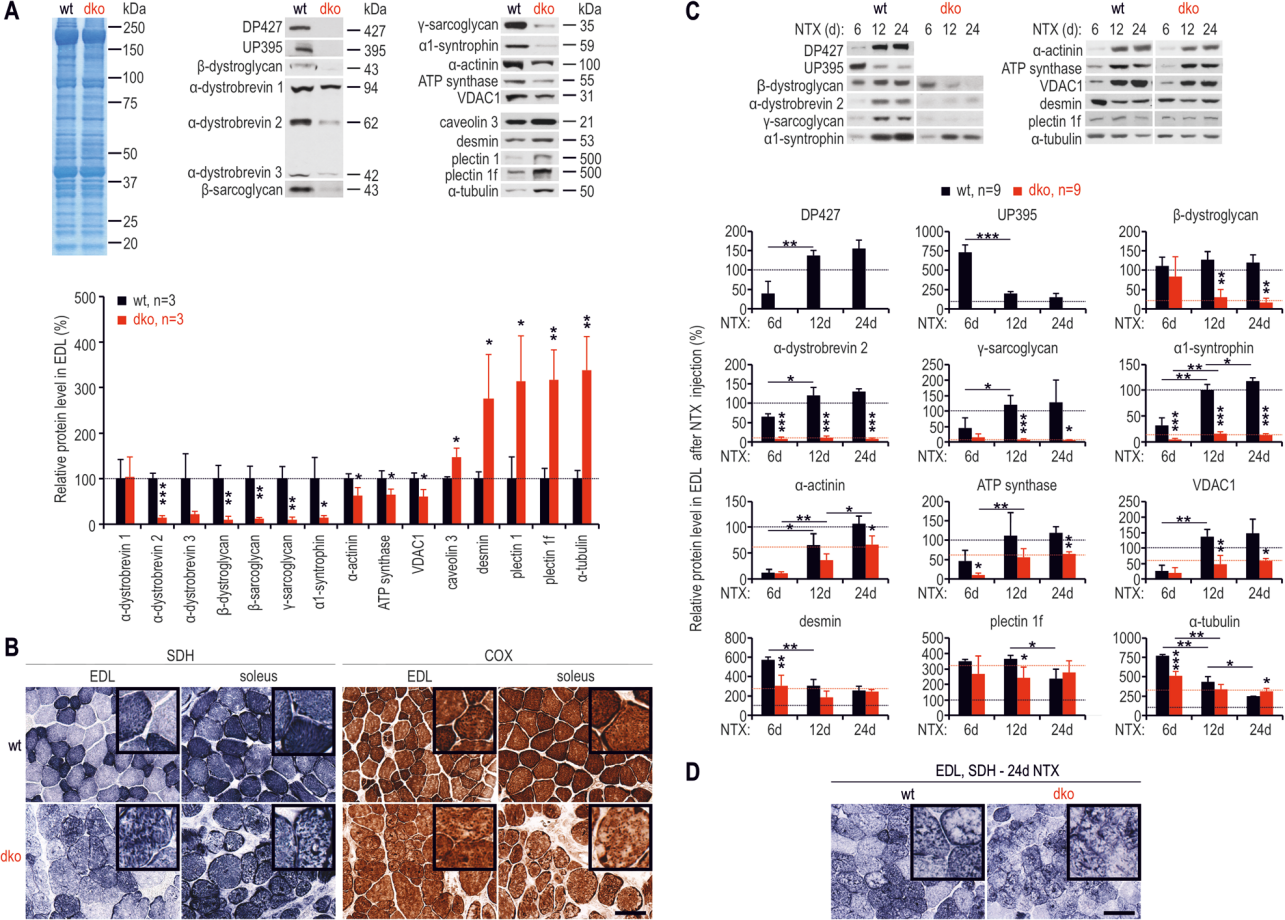
Loss of dystrophin DP427 and utrophin in skeletal muscle leads to proteomic changes and alterations in the organization of the mitochondrial network. Immunoblotting analysis of EDL muscles (**A**) and EDL muscles after NTX treatment (**C**) with densitometric evaluation of band intensities relative to wt levels. Coomassie-stained polyacrylamide gels were used as normalizing controls. The dotted black horizontal line in **A** shows protein levels in uninjected wt muscles, and the dotted black and red horizontal lines in **C** show protein levels in uninjected wt and dko EDL muscles. Frozen sections of EDL and soleus muscles from wt and dko mice stained for the mitochondrial enzymes SDH and COX (**B**) and of EDLs from wt and dko mice isolated 24 days post-NTX and stained for SDH (**D**). The insets show magnifications of representative section fragments. Bars, 50 μm. The results in **A** and **C** are shown as the mean values + SDs. *n*, number of biological replicates (EDL muscles). ^*^p < 0.05; ^**^p < 0.01; ^***^p < 0.001 (*t*-test).

Our comparative immunofluorescence microscopy and Western blot analyses of wt and dko *extensor digitorum longus* (EDL) muscles confirmed markedly reduced DGC/UGC protein contents in dko tissues (Figs. 1A and S1). Specifically, we detected reduced amounts of α-dystrobrevin 2 and 3, β-dystroglycan, β- and γ-sarcoglycan, and α1-syntrophin, along with the myofibre differentiation marker, α-actinin. In contrast, the level of predominantly neuromuscular junction (NMJ)-associated α-dystrobrevin 1 was unaltered, and elevated amounts of caveolin 3, and cytoskeletal proteins, such as desmin, plectin, α-tubulin and vimentin, were observed [38]. An anti-vimentin antibody labelled cells exclusively outside of myofibres, revealing many more nonmyogenic cells in dko EDLs, and desmin was detected in myofibres, with a particularly high signal in small-diameter regenerating fibres of dko muscles (Fig. S1A). In turn, anti-pan plectin and anti-α-tubulin antibodies marked both non-myogenic cells and muscle fibres, with a particularly strong signal in the dko samples. In agreement with the staining pattern of the pan-plectin antibody, the plectin isoform 1f signal was observed on the sarcolemma, primarily in regenerating dko fibres, and plectin 1 was found in nonmyogenic cells, and costameres and perinuclear regions of isolated fibres (Fig. S1A, B). Despite changes in protein content, the costameric localization of plectin and desmin remained unchanged (Fig. S1B).

In contrast to the intact organization of costameres in isolated EDL fibres, both the content and organization of the mitochondrial network were altered in dko muscles. In particular, the amounts of the mitochondrial marker proteins ATP synthase and VDAC1 were reduced in EDL protein lysates (Fig. 1A), and histochemical staining of succinate dehydrogenase (SDH) and cytochrome c oxidase (COX) in frozen EDL and soleus sections revealed decreased activity of mitochondria and disorganization of the mitochondrial network (Fig. 1B). Additionally, there was a clear distinction of fibre types in wt samples, whereas such a distinction was barely noticeable in dko muscles.

### NTX-induced regeneration reveals a correlative increase in DGC and mitochondrial proteins during muscle regeneration

We next injected notexin (NTX) into wt and dko EDLs to degenerate muscle fibres and induce their synchronous regeneration from activated muscle stem cells (MuSCs) to confirm that the observed proteomic changes did not stem solely from the presence of immature fibres (Fig. S2). Analyses revealed the complementary synthesis of UP395 and DP427 during the regeneration of wt EDLs (Figs. 1C and S3A). Specifically, high amounts of utrophin were observed on the sarcolemma at 6 days post NTX treatment, whereas low amounts of utrophin were limited to NMJs in the later stages of regeneration (Fig. S3A). On the other hand, DP427 was present at low levels on the sarcolemma at the earliest differentiation time point, and its amount was significantly greater at 12 and 24 days after NTX treatment (Figs. 1C and S3A). Interestingly, all tested DGC components, including α-dystrobrevin-2, γ-sarcoglycan and α1-syntrophin, but apart from β-dystroglycan, which was already high at the earliest time point tested, followed the dystrophin synthesis pattern (Figs. 1C and S3). During regeneration, the levels of the myofibre differentiation marker α-actinin, as well as mitochondrial marker proteins, also increased, whereas the amounts of desmin, plectin 1f and α-tubulin followed a reverse synthesis profile, in agreement with the results observed in the nontreated muscles (Figs. 1A and S1).

Analyses of dko muscles revealed β-dystroglycan levels comparable to those of the wt samples at 6 days post NTX and decreasing amounts at later differentiation stages, whereas the other DGC components were present at very low quantities at the earliest analysed time point (Figs. 1C and S3). Much lower levels of α-actinin and mitochondrial marker proteins were also detected in dko samples, especially on days 12 and 24 post NTX injection, whereas anti-α-tubulin, anti-desmin and anti-plectin 1f antibodies usually presented lower values in early regeneration stages and tended toward higher levels on the last analysed day. Additionally, 24 days after NTX injection, the mitochondrial network in wt samples exhibited a uniform localization pattern (with rare exceptions, as shown in the inset), whereas in dko muscles, the staining was weaker and disorganized (Fig. 1D).

### Dko EDL fibres show increased regeneration during early differentiation but reduced survival during later stages of differentiation

**Fig. 2:**
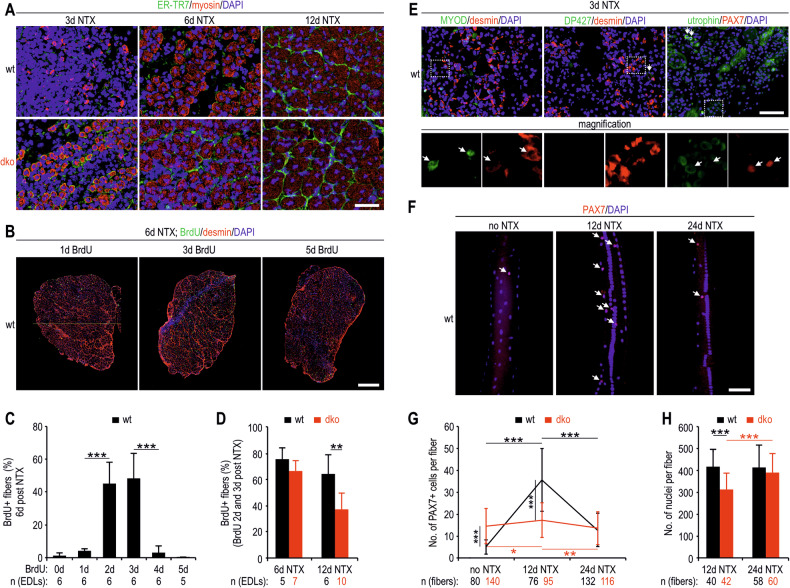
Dko EDL fibres show enhanced formation during early differentiation but high mortality rates in later differentiation stages. **A** Frozen sections of EDL muscles from wt and dko mice 3, 6 and 12 days after NTX injection immunostained for ER-TR7 (fibroblast marker), myosin and nuclei (DAPI). Representative images (**B**) and quantification of the percentage of fibres containing BrdU+ nuclei 6 days after NTX injection from wt EDL muscle sections immunostained for BrdU, desmin and DAPI (**C**). The mice were either not injected with BrdU or were injected subcutaneously with BrdU 1, 2, 3, 4, or 5 days after NTX treatment. **D** Quantification of the percentage of wt and dko EDL fibres containing BrdU+ nuclei at 6 and 12 days post-NTX injection. BrdU was injected twice, 2 and 3 days after NTX treatment. **E** Frozen sections of EDL muscles from wt mice 3 days post-NTX injection immunostained for myoblast determination protein 1 (MYOD), DP427, utrophin, desmin, and nuclei (DAPI). The insets show a lack of DP427 in desmin+ myoblasts and the presence of utrophin in proliferating PAX7+ cells. Representative images (**F**) and quantification of PAX7+ nuclei in fibres isolated from uninjected or NTX-treated wt and dko EDL muscles (**G**). **H** Evaluation of the number of nuclei in wt and dko fibres collected from muscles injected with NTX. The results in **C**, **D** and **H** are presented as the means + SD and those in **G** as the mean values ± SDs. *n*, number of biological replicates (EDL muscles in **C**, **D** and fibres from EDL muscles in **G**, **H**). Bars, 50 μm (**A**, **E**, **F**) and 300 μm (**B**). ^*^p < 0.05; ^**^p < 0.01; ^***^p < 0.001 (*t*-test in **C**, **D** and *z*-test in **G**, **H**).

Proteomic analyses of NTX-injected EDLs revealed that the loss of DP427 and utrophin led to faster generation of myofibres (significantly lower amounts of desmin and α-tubulin in dko samples than in control samples at 6 days post NTX injection) and either hindered regeneration in the later stages of differentiation or premature death and the generation of new myofibres (significantly lower α-actinin and higher α-tubulin levels at 24 days post NTX injection) (Fig. 1C). To assess the time required to generate muscle fibres, we prepared frozen cryosections from muscles harvested 3, 6 and 12 days after surgery and double immunolabelled them for myosin and the fibroblast marker ER-TR7. Indeed, the dko samples presented larger myosin-positive fibres than the wt samples did at the earliest time point analysed (Fig. 2A). To test how the lack of DP427 and utrophin affects fibres at later stages of differentiation, we first determined the timing of myoblast proliferation after NTX treatment. Immunofluorescence microscopy analysis revealed high numbers of BrdU-labelled nuclei when BrdU was injected 2 and 3 days after NTX treatment (Fig. 2B, C), indicating that cell proliferation is most robust at these time points. Wt and dko mice were then injected with BrdU twice, 2 and 3 days after NTX treatment, and the muscles were harvested on days 6 and 12 after NTX injection. While the number of nuclei in myofibres containing BrdU-labelled nuclei was comparable on day 6, approximately 50% fewer dko-labelled myofibres were observed 12 days after NTX treatment (Figs. 2D and S4), revealing their reduced survival.

We next examined the time points at which DP427 and utrophin begin to be synthesized following NTX injection and whether their synthesis affects myofibre regeneration from activated MuSCs. We did not observe DP427 in proliferating PAX7-positive cells, whereas utrophin was visible both in myoblasts and in newly formed myofibres (Fig. 2E). We then isolated single fibres from untreated and NTX-injected EDL muscles and immunolabelled them (Fig. 2F) to test whether the number of PAX7+ cells in the dko muscles could explain the increased myofibre formation in the dko EDLs. Consistent with our hypothesis, the analysis revealed a threefold greater number of PAX7+ cells on dko fibres than on wt fibres isolated from untreated muscles (Fig. 2G). Interestingly, the number of PAX7+ cells in wt EDLs increased markedly, remained elevated until 12 days post NTX injection, and then decreased to lower numbers (Figs. 2G and S5). In contrast, the number of these cells on the dko fibres remained approximately constant. PAX7+ cells on the dko muscles were able not only to generate new myofibres after NTX treatment but also to regenerate myofibres after subsequent degenerative processes, as the number of myofibre nuclei in the dko fibres increased to wt levels from days 12-24 after NTX treatment (Fig. 2H). Overall, these data indicate that the absence of DP427 and utrophin does not prevent the regeneration of myofibres from MuSCs.

### Fibre degeneration in dko muscles is caused by intrinsic cellular alterations independent of changes in NMJs and other cell types

**Fig. 3:**
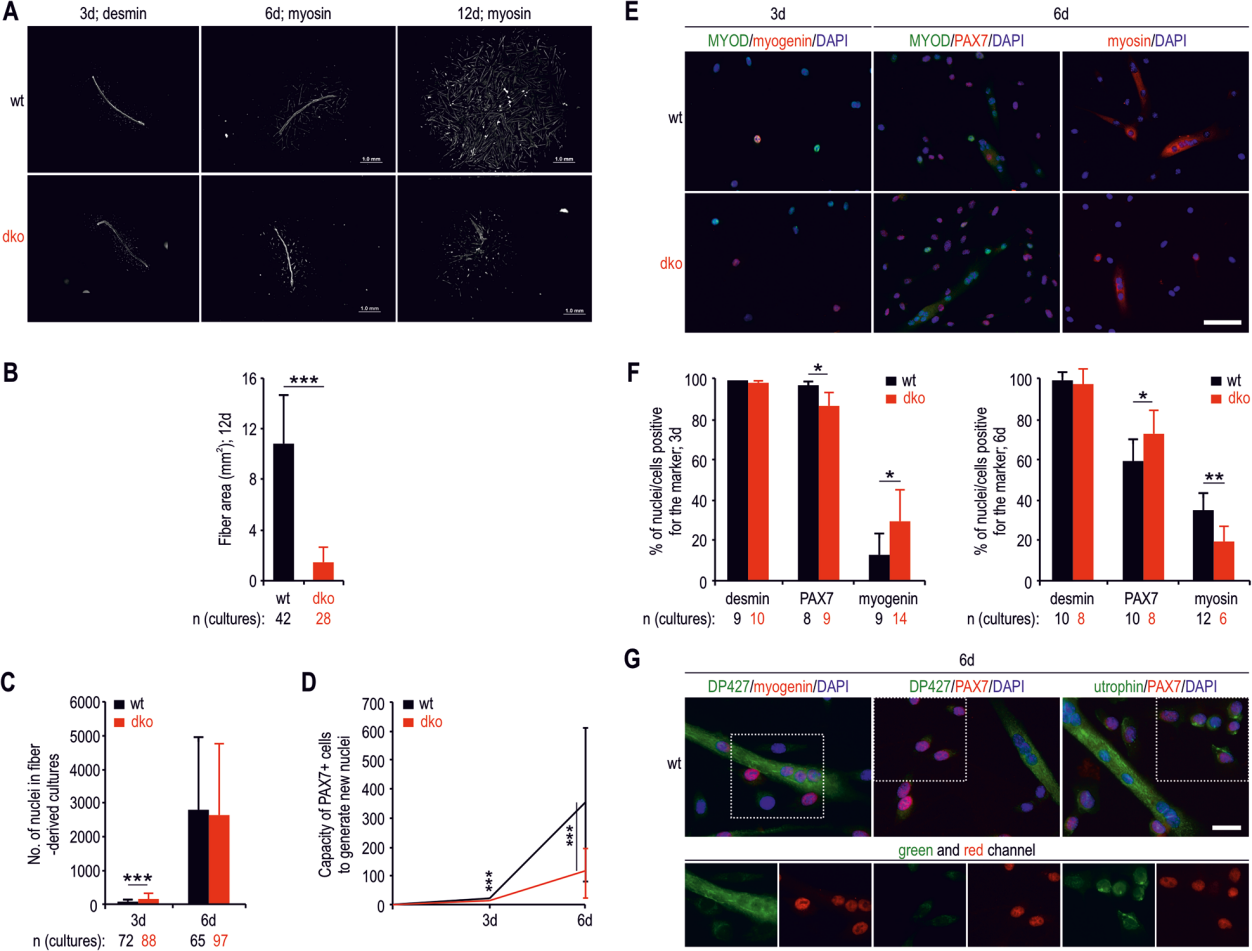
Dko fibre degeneration is caused by intrinsic cellular alterations independent of changes in NMJs and other cell types. Fibre-derived wt and dko cultures immunostained for either desmin (3 days post-fibre plating) or myosin (6 and 12 days post-fibre plating) (**A**) and the quantification of total myotube areas in wt and dko cultures 12 days post-fibre plating (**B**). Evaluation of the number of nuclei in fibre-derived cultures 3 and 6 days post-fibre plating (**C**) and normalization of the number of nuclei to the number of PAX7+ nuclei in uninjected EDL fibres (**D**; see also Fig. 2G). Images of 3- and 6-day-old fibre-derived wt and dko cultures immunolabelled for differentiation markers (**E**) and quantification of the percentage of nuclei/cells positive for desmin, PAX7, myogenin and myosin (**F**). **G** Fibre-derived wt cultures immunostained for DP427, myogenin, utrophin and PAX7 6 days post-fibre plating. The results in **B**, **C** and **F** are shown as the mean values + SDs and those in **D** as the mean values ± SDs. *n*, the number of biological replicates (cultures from single EDL fibres). Bars, 50 μm in **E** and 20 μm in **G**. ^*^p < 0.05; **p < 0.01; ^***^p < 0.001 (*z*-test in **B**, **C** and *t*-test in **F**).

Myofibre degeneration could be a consequence of intrinsic changes in myofibres or NMJs or general alterations in the muscle tissue environment and impaired interactions with other cell types. To test this hypothesis, we isolated single myofibres and allowed activated MuSCs to migrate from them, proliferate and generate new myotubes. As early as day 6 after plating, myosin-labelled dko cultures presented reduced areas (Fig. 3A). This difference was even more pronounced on day 12, when we also observed the death of dko myotubes (Fig. 3A, B). We also noted significantly greater numbers of nuclei in the dko cultures 3 days after plating, but on day 6, these numbers were similar in the wt and dko wells (Fig. 3C). After normalizing the number of nuclei to the number of PAX7+ cells on myofibres of untreated muscles, we observed lower numbers of nuclei in dko cultures (Fig. 3D), indicating their reduced ability to give rise to new nuclei.

We then immunolabelled wt and dko cultures at days 3 and 6 after fibre plating (Fig. 3E). Consistent with the data showing enhanced dko fibre formation at the early stage of differentiation (Fig. 2A), dko cultures also presented a greater percentage of myogenin+ nuclei on day 3, whereas on day 6, the opposite pattern was observed, with a greater percentage of PAX7+ cells and a lower percentage of nuclei within myosin+ cells/myotubes (Fig. 3F). Analyses also revealed the presence of utrophin in proliferating PAX7+ cells and myotubes, whereas significant amounts of DP427 were present only in myotubes (Fig. 3G). Together, these results demonstrate that greater numbers of PAX7+ cells in dko fibres determine enhanced fibre formation; however, these fibres die at later differentiation stages, as evidenced by the reduction in the number of BrdU-labelled nuclei (Fig. 2D) and the myosin staining of myotubes (Fig. 3A). Moreover, the data indicate that premature death of dko fibres is not a consequence of damaged NMJs and defective interactions with neurons or other cell types.

### siRNA-mediated downregulation of the dystrophin and utrophin genes leads to pathological abnormalities in myoblasts and muscle fibres

**Fig. 4:**
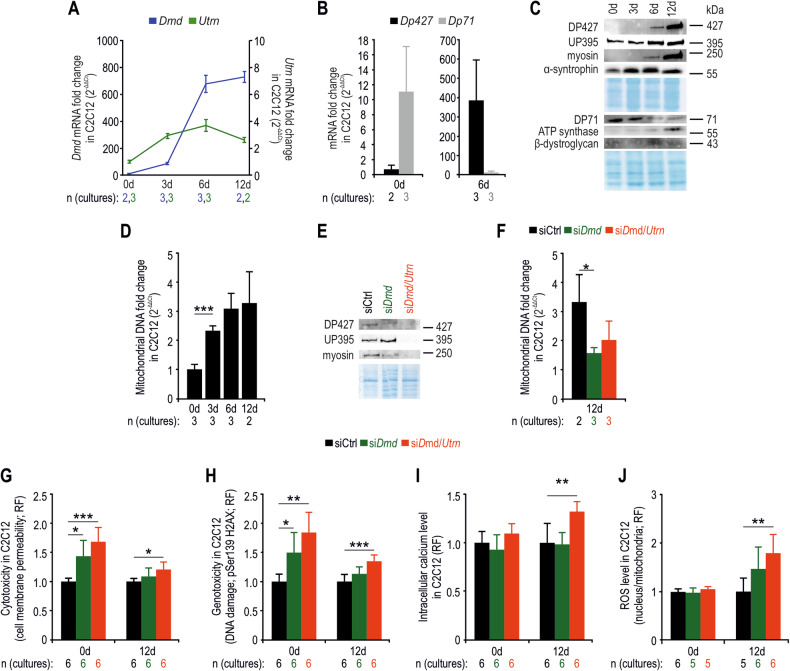
Proliferating and differentiated dystrophin and utrophin-deficient C2C12 cells show increased cyto- and genotoxicity. RT-qPCR analyses of *Dmd* and *Utrn* (**A**), and *Dp427* and *Dp71* (**B**) mRNAs in the C2C12 cell line at different time points during differentiation. Immunoblot analyses of C2C12 protein extracts from days 0, 3, 6 and 12 of differentiation (**C**) and from day 12 of differentiation after siRNA delivery (**E**). Coomassie-stained polyacrylamide gels were used to normalize the protein content. Quantification of mitochondrial levels in C2C12 cells based on qPCR results of mitochondrial (*Nd1*) and nuclear (*Hk2*) DNA markers during differentiation (**D**) and in differentiated cells following RNA interference (**F**). Relative fluorescence (RF) signals of cytotoxicity (cell membrane permeability) (**G**), genotoxicity (DNA damage) (**H**), intracellular calcium (**I**) and ROS (**J**) in dystrophin- and dystrophin/utrophin-deficient C2C12 cells. The results in **A** are shown as the mean values ± SDs and those in **B**, **D**, **F**–**J** as the mean values + SDs. *n*, number of biological replicates (cell cultures). ^*^p < 0.05; ^**^p < 0.01; ^***^p < 0.001 (*t*-test).

Because some of the observed changes in fibre-derived cultures could be due to prior alterations in the muscle tissue milieu, we decided to downregulate the *Dmd* and *Utrn* genes in a commonly used murine C2C12 muscle cell line (Figs. 4 and S6). Quantitative PCR analyses revealed very low dystrophin mRNA levels in proliferating cells (day 0) and their rapid increase during differentiation (from day 3 to day 12; Fig. 4A), along with the early and late differentiation markers myogenin (*MyoG*) and myosin (*Myh1*), respectively (Fig. S6A). On the other hand, utrophin mRNAs were at higher levels in the proliferation stage but increased to much lower levels during early differentiation and then declined on day 12. Subsequent analysis revealed that *Dp71* mRNAs were the most abundant dystrophin transcripts in proliferating cells in contrast to myotubes, where *Dp427* was predominantly transcribed (Fig. 4B). In agreement, the DP71 protein was the only dystrophin detected on day 0, but following differentiation initiation, this dystrophin was replaced by DP427 (Fig. 4C). Among the utrophin proteins, UP395 was detected at all stages of differentiation (Fig. 4C). In accordance with the proteomic data obtained after muscle degeneration with NTX (Fig. 1C), we also detected increasing levels of DGC components (α-syntrophin and β-dystroglycan) and the mitochondrial protein marker ATP synthase (Fig. 4C) as well as mitochondrial DNA (Fig. 4D). Immunofluorescence analysis with an antibody that does not distinguish dystrophin from utrophin (Fig. S7) also revealed the presence of dystrophin/utrophin at all stages of myotube formation (Fig. S6B).

After siRNA-mediated downregulation of the *Dmd* and *Utrn* genes in proliferating cells and myotubes (Figs. 4E and S6C), we observed several pathological changes (Fig. 4F–J). Proliferating dystrophin-deficient and dystrophin- and utrophin-deficient cells presented increased cyto- and genotoxicity (Fig. 4G, H). Similarly, differentiated myotubes lacking dystrophin and utrophin presented increased toxicity (Fig. 4G, H), increased intracellular calcium levels (Fig. 4I) and increased ROS levels (Figs. 4J, S6D and S8). Moreover, reduced myosin (Fig. 4E) and mitochondrial levels were observed (Fig. 4F), indicating impaired differentiation of C2C12 cells treated with siRNAs targeting *Dmd*, and *Dmd* and *Utrn* transcripts.

**Fig. 5:**
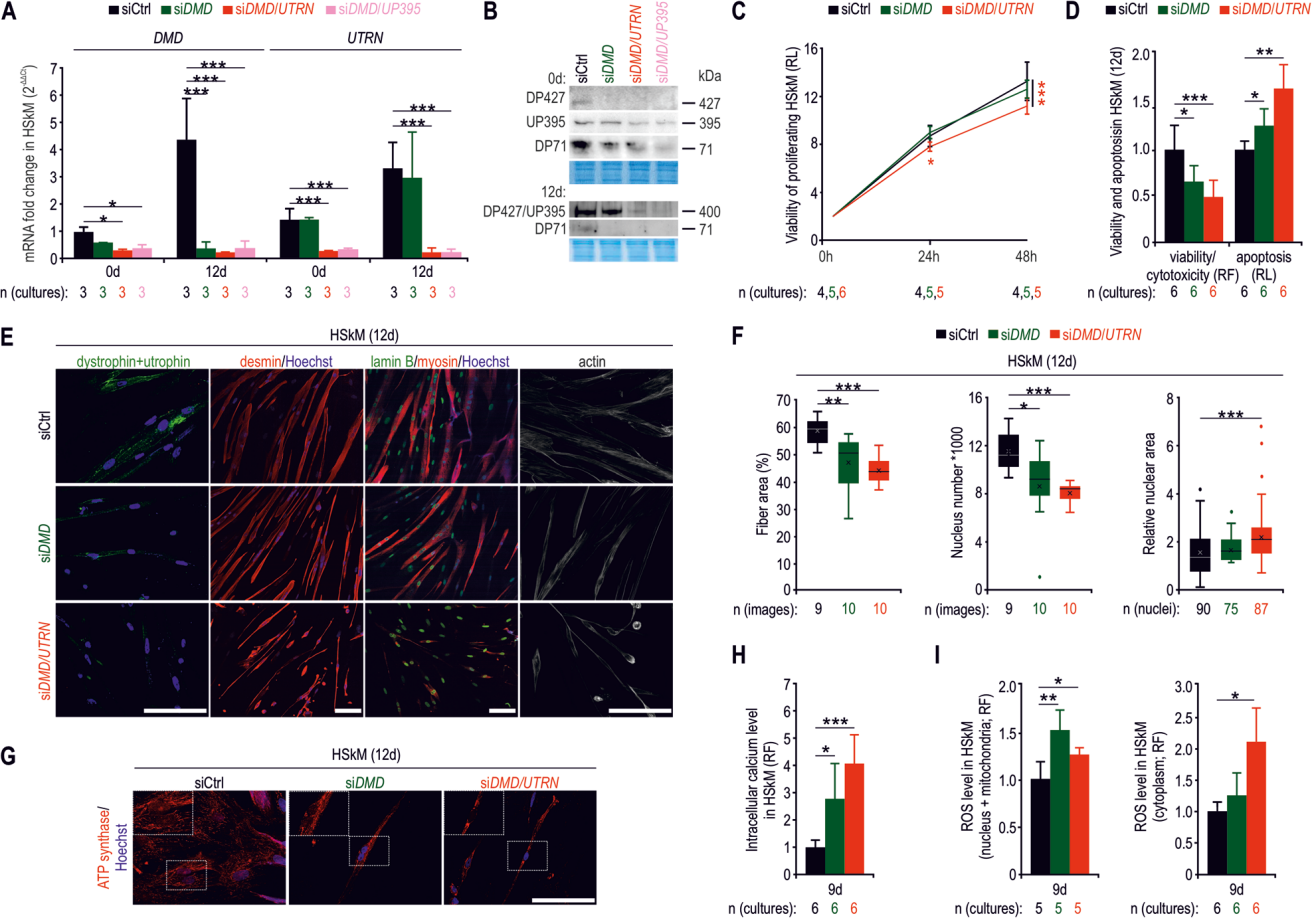
Downregulation of the dystrophin and dystrophin/utrophin genes in proliferating or differentiated HSkM cells leads to profound pathological changes. RT-qPCR (**A**) and immunoblotting (**B**) analyses of proliferating (0 d) and differentiated (12 d) HSkM cells after siRNA administration. Coomassie-stained polyacrylamide gels were used to normalize the protein content in **B**. **C** Assessment of the viability of proliferating HSkM cells for 48 h after downregulation of the *DMD* or *DMD*/*UTRN* genes. **D** Analysis of the viability and apoptosis of differentiated HSkM cells transfected with siRNA. Immunostaining of HSkM cells on day 12 of differentiation for dystrophin/utrophin, desmin, lamin B, myosin and actin (**E**) and ATP synthase (**G**). Nuclei were labelled with Hoechst (blue channel). The insets in **G** show the separate red channel with ATP synthase labeling. Bars, 100 μm. **F** Quantification of fibre plate coverage, nuclear density per targeted area, and relative nuclear area in HSkM myotubes following RNA interference. Relative fluorescence signals of intracellular calcium (**H**) and ROS (**I**) levels in siRNA-treated HSkM myotubes. In **A**, **D**, **H** and **I**, the data are shown as the mean values + SDs and in **C** and **F** as the mean values ± SDs. RL, relative luminescence; RF, relative fluorescence; *n*, number of biological replicates. ^*^p < 0.05; ^**^p < 0.01; ^***^p < 0.001 (*t*-test in **A**, **C**, **D**, **F**, **H**, **I** and *z*-test in **F** (nuclear area)).

To further assess whether the same phenotype could be observed in human myoblasts and myotubes, we delivered siRNAs to primary human skeletal muscle (HSkM) myoblasts and subjected them to proliferation or differentiation (Figs. 5 and S9A–C). SiRNA-mediated downregulation of the *DMD* and *UTRN* genes (Fig. 5A, B) reduced the content of DGC/UGC components (Fig. S9D) and decreased the viability of HSkM myoblasts (Fig. 5C) and myotubes (Fig. 5D). The culture plates containing myotubes lacking dystrophin and utrophin also presented reduced fibre areas, fewer nuclei, and increased nuclear areas (Figs. 5E, F and S10A). Although quantitative analyses revealed unaltered mitochondrial and mitochondrial DNA contents in dystrophin-deficient HSkM myotubes (Fig. S10B), ATP synthase-positive aggregates were observed, particularly in those treated with si*DMD*/*UTRN* (Fig. 5G). Furthermore, increased intracellular calcium and ROS levels were observed (Fig. 5H, I). These data indicate that contraction-induced changes in the sarcolemma are not a prerequisite for profound pathological alterations in myoblasts and myotubes lacking either dystrophin or dystrophin and utrophin.

### Utrophin UP395 partially compensates for the lack of DP71 in proliferating cells

**Fig. 6:**
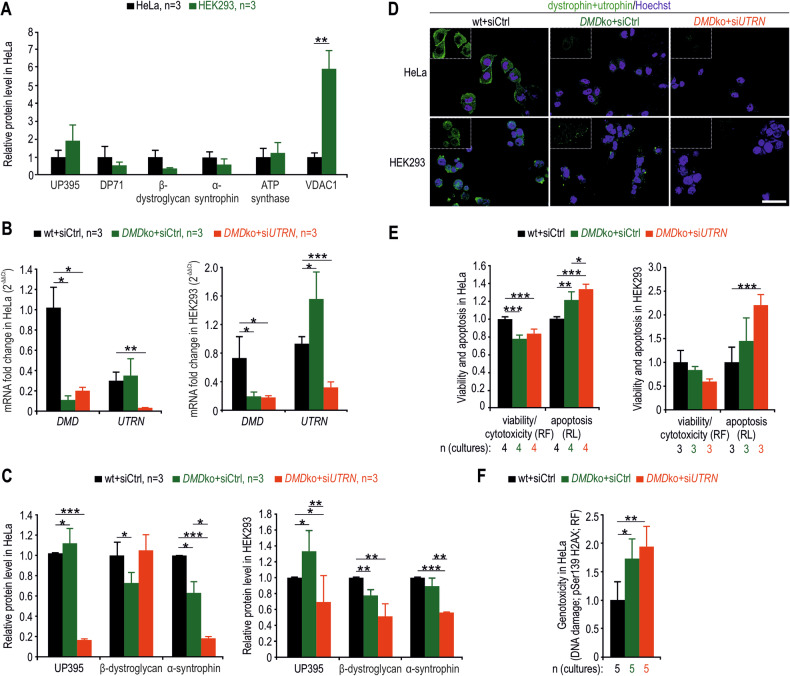
*DMD* ko HeLa and HEK293 cell lines show upregulation of the *UTRN* gene, a decrease in the level of DGC proteins, and reduced viability. **A** Quantitative evaluation of immunoblot band intensities from HeLa and HEK293 cell protein extracts, normalized to HeLa signals. Evaluation of dystrophin and utrophin levels in siRNA-treated wt and *DMD* ko HeLa and HEK293 cells via RT-qPCR (**B**), immunoblotting analysis (**C**), and immunofluorescence microscopy (**D**). Immunoblotting was also used to assess β-dystroglycan and α-syntrophin levels (**C**). Coomassie-stained polyacrylamide gels were used for protein normalization in **A** and **C**. The insets in **D** show the separate green channel for dystrophin/utrophin labelling (without the Hoechst-labelled nuclei). Bar in **D**, 50 μm. Analysis of viability and apoptosis (**E**) and DNA damage (**F**) in wt and *DMD* ko HeLa cells after siRNA treatment. The results in **A**–**C** and **E**, **F** are shown as the mean values + SDs. RF, relative fluorescence; RL, relative luminescence; *n*, number of biological replicates. ^*^p < 0.05; ^**^p < 0.01; ^***^p < 0.001 (*t*-test).

Infiltrating immune and intrinsic to the muscle proliferating cells are critical in tissue repair during regeneration. To address the importance of dystrophin proteins in proliferating cells, we used model HeLa and HEK293 cells. Both lacked DP427 and differed in DP71, UP395, DGC/UGC and mitochondrial protein content (Fig. 6A). After CRISPR/Cas9-mediated *DMD* gene deletion (Figs. S11 and S12), both cell types presented reduced DGC and increased UP395 protein levels (Fig. 6B–D), indicating a universal compensatory mechanism based on the upregulation of the *UTRN* gene in the absence of either DP427 or DP71. Indeed, DP71 deficiency reduced the survival of HeLa and HEK293 cells (Fig. 6E), which was associated with increased DNA damage (Fig. 6F), and the additional lack of UP395 markedly worsened the phenotype (Figs. 6E, F and S13).

**Fig. 7:**
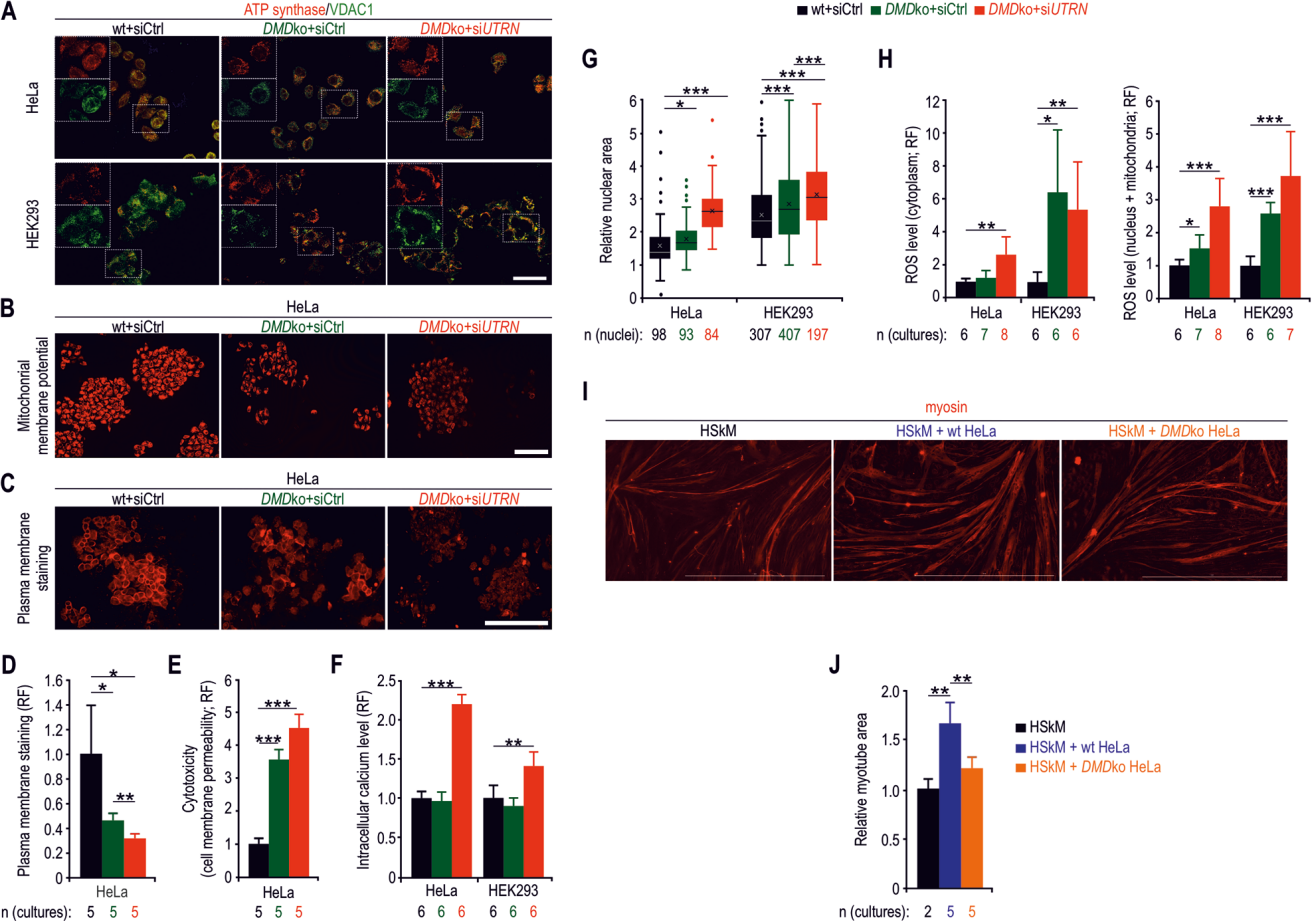
The presence of DP71 in proliferating cells maintains their proper function and facilitates myotube differentiation in cocultures. Fluorescence microscopy of siRNA-treated HeLa and HEK293 cells labelled with antibodies against the mitochondrial proteins, ATP synthase and VDAC1 (**A**) and the mitochondrial membrane potential indicator (**B**). The insets in **A** show the separate red channel with ATP synthase labelling and the green channel with VDAC labelling. Images of dystrophin- and utrophin-deficient HeLa cells labelled with the plasma membrane stain (**C**) and quantification of fluorescence signals via a Tecan SPARK plate reader (**D**). Relative fluorescence signals of cytotoxicity (cell membrane permeability) (**E**), intracellular calcium (**F**) and ROS (**H**) levels in wt and *DMD* ko HeLa cells after siRNA treatment. **G** Relative nuclear areas in siRNA-treated HeLa and HEK293 cells. Fluorescence microscopy of HSkM myotubes immunolabelled for myosin (**I**) and quantification of the signals from the images (**J**). HSkM cells were either differentiated alone or cocultured with HeLa cells (wt or *DMD* ko). In **D**–**F**, **H** and **J**, the data are shown as the mean values + SDs and in **G** as the mean values ± SDs. Bars, 50 μm in **A**, 100 μm in **B**, **C** and 1 mm in **I**. RF, relative fluorescence; n, number of biological replicates. ^*^p < 0.05; ^**^p < 0.01; ^***^p < 0.001 (*t*-test in **D**–**F**, **H** and **J**, and *z*-test in **G**).

To determine which cell structures and processes may be responsible for the reduced survival of DP71-deficient proliferating cells, we assessed the number, localization and function of mitochondria, the function of the plasma membrane and the size of the nucleus. As in HSkM dystrophin-deficient cells, overall mitochondrial levels were unchanged in *DMD* ko HeLa and HEK293 cells despite lower ATP synthase levels (Fig. S14A, B), but their localization changed from dispersed to more aggregated (Fig. 7A), and their membrane potential was reduced (Figs. 7B and S14C). Similarly, the plasma membrane was severely damaged, as indicated by reduced plasma membrane staining, increased plasma membrane permeability and increased intracellular calcium levels (Figs. 7C–F and S15). Furthermore, the nuclear areas of DP71-deficient cells were enlarged (Fig. 7G). These functional and structural changes were associated with elevated ROS levels in both the cytoplasm and mitochondria/nuclei (Fig. 7H). Finally, we evaluated the effect of the loss of DP71 in proliferating HeLa cells on myotube differentiation. Importantly, wt HeLa cells positively influenced the generation of myotubes, whereas no such effect was observed in cocultures of myoblasts and *DMD* ko HeLa cells (Fig. 7I, J). Taken together, these data show that the lack of DP71 in proliferating cells can lead to profound pathological changes in various cell structures and apoptosis and that this phenotype can be partially compensated by UP395. Moreover, our results indicate that the presence of DP71 in proliferating cells facilitates myotube differentiation.

### RNA sequencing of dystrophin- and utrophin-deficient HeLa cells and HSkM myotubes reveals profound transcriptomic changes related to phenotypic abnormalities

**Fig. 8:**
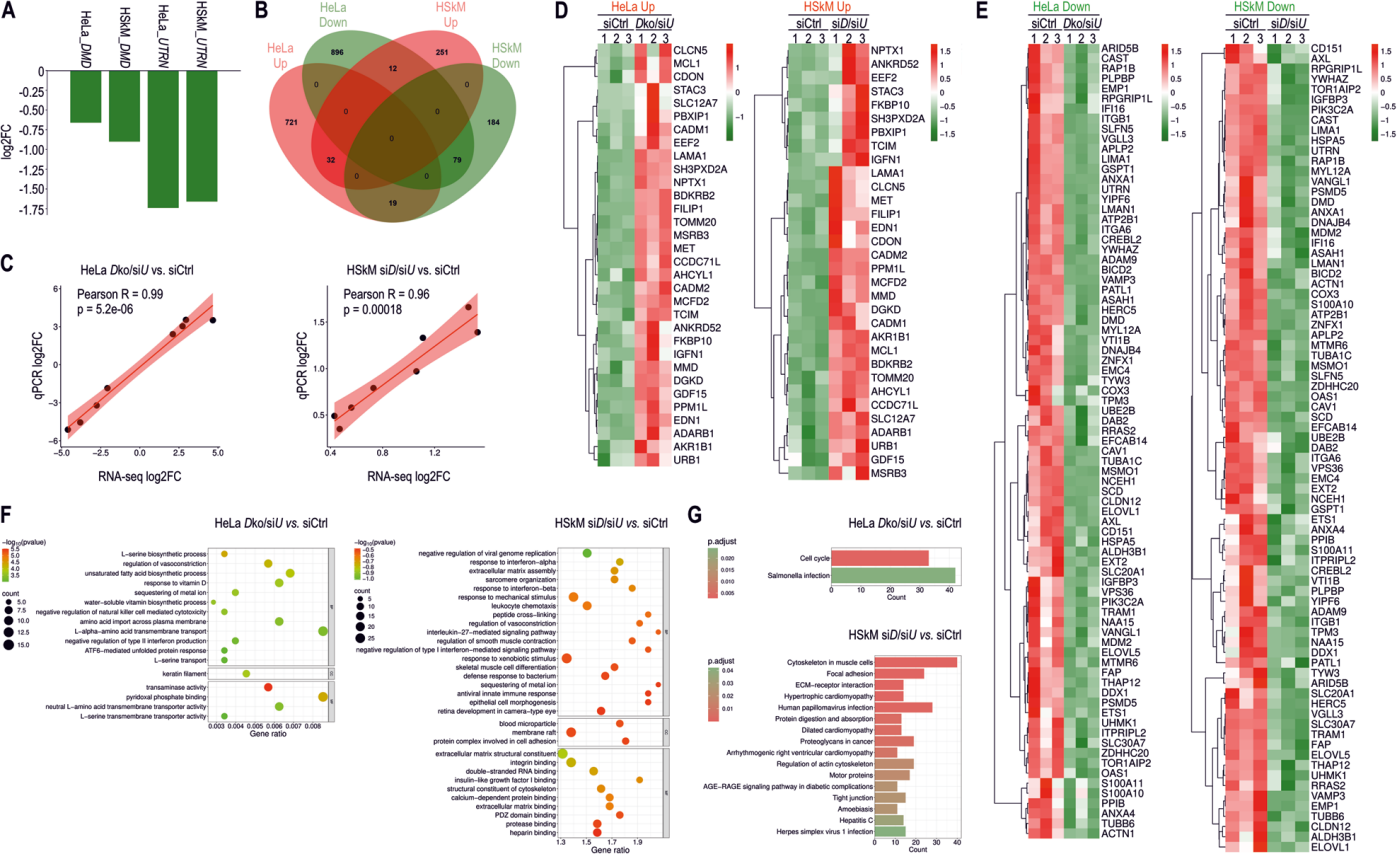
Dystrophin- and utrophin-deficient HeLa cells and HSkM myotubes show profound transcriptomic changes that correlate with phenotypic alterations. **A** Expression levels of the *DMD* and *UTRN* genes in *DMD*ko + si*UTRN* (*D*ko/si*U*) HeLa cells and si*DMD* + si*UTRN* (si*D*/si*U*) HSkM myotubes compared with control (siCtrl) samples, presented as log2FC values. **B** Venn diagram showing the number of up- and down-regulated genes in dystrophin- and utrophin-deficient HeLa cells and HSkM myotubes compared with control samples. Changes in expression fold changes were tested via the Wald-test. The obtained *p*-values obtained from the statistical tests were adjusted for multiple testing using the Benjamin-Hochberg method (FDR ≤ 0.05). **C** Pearson correlation analysis of gene expression changes for *D*ko/si*U* HeLa and si*D*/si*U* HSkM myotubes, comparing log2FC values obtained from RNA-seq with those measured by RT-qPCR relative to their respective controls. The correlation plot illustrates a strong concordance between the two methods, confirming the reliability of the RNA-seq-derived expression profiles. **D,** **E** Heatmaps of normalized expression values for genes whose expression was significantly upregulated (**D**) or downregulated (**E**) in *D*ko/si*U* HeLa cells and si*D*/si*U* HSkM myotubes. The columns represent the two experimental cell types, and the rows correspond to genes whose log2FC > 0.5 and adjusted *p*-value < 0.05 under both conditions. The colour gradient encodes relative expression levels, with higher red intensity indicating a higher expression level in one cell type relative to the other and higher green intensity indicating relatively lower expression. **F** Gene Ontology (GO) enrichment analysis of differentially expressed genes in *D*ko/si*U* HeLa cells and si*D*/si*U* HSkM myotubes compared with their respective controls. Each circle represents a GO term, and its size reflects the number of genes enriched in that category. The significance of enrichment is shown as the −log10(*p*-value), with GO terms of greater statistical significance (lower *q* values) depicted in red and those of lower significance (higher q-values) depicted in green. BP – biological process; CC – cellular component; MF – molecular function. **G** KEGG pathway enrichment analysis of differentially expressed genes in *D*ko/si*U* HeLa cells and si*D*/si*U* HSkM myotubes vs. their siCtrl samples. The bar plot shows significantly enriched pathways ranked by gene count (x‑axis). The colour gradient indicates the adjusted p‑value (adj.p), with red denoting highly significant pathways (p < 0.01) and green indicating less significant pathways (p ≈ 0.20). “Count” represents the number of differentially expressed genes from HeLa and HSkM cells assigned to each KEGG pathway, reflecting the extent of biological process involvement in response to dystrophin and utrophin loss.

We then asked whether we could observe transcriptomic changes corresponding with the pathological phenotype in dystrophin- and utrophin-deficient HeLa cells and HSkM myotubes (Fig. 8A). Differential gene expression (DEG) analysis revealed 772 up- and 987 downregulated genes in HeLa *DMD* ko cells following *siUTRN* treatment, whereas in HSkM cells treated with *siDMD/UTRN*, 295 up- and 282 downregulated genes were identified relative to the control (Figs. 8B, C and S16). Among these genes, those that were common to both cell types and presented either increased or decreased expression levels are shown in Fig. 8D, E. Genes with significantly increased expression in dystrophin- and utrophin-deficient HeLa cells and HSkM myotubes were associated with oxidative stress (*AKR1B1*, *MSRB3*), mitochondria (*TOMM20*, *MCL1*, *EEF2*), apoptosis (*MCL1*, *EDN1*, *GDF15*, *TCIM*), muscle regeneration and cell growth (*MET*, *IGFN1*, *CDON*, *SH3PXD2A*), fibrosis and tissue remodelling (*EDN1*, *FKBP10*, *LAMA1*), metabolism (*AKR1B1* – also involved in ROS detoxification, *DGKD*, *SLC12A7*), signalling (*STAC3*, *CADM1/CADM2*, *CLCN5*, *PPM1L*, *ANKRD52*), immune response and cell adhesion (*NPTX1*), protein synthesis (*URB1*), cell proliferation (*AHCYL1*), gene transcription (*PBXIP1*), and cytoskeleton organization (*CCDC71L*) (Fig. 8D). Similarly, genes with decreased expression in both cell lines were related to muscle regeneration and cell growth (*ITGA6*, *ITGB1*, AXL, ETS1), fibrosis and tissue remodelling *(FAP*, *ANXA1*, *CAV1*), metabolism (*SCD*, *MSMO1*, *ELOVL1*, *ELOVL5*), oxidative stress (*ALDH3B1*, *S100A10*, *S100A11*), mitochondria (*COX3*, *ATP2B1*), apoptosis (*MDM2*, *HSPA5*), cellular function and signalling (*RAP1B*, *PIK3C2A*, *RRAS2*), transport and cytoskeleton organization (*VAMP3*, *TUBB6*, *TUBA1C*, *ACTN1*), cell-cell interactions (*CD151*), tight junction formation (*CLDN12*), heparin biosynthesis and cell signalling (*EXT2*), and protein degradation (*UBE2B*) (Fig. 8E).

To gain insight into the biological processes affected by combined dystrophin and utrophin suppression, Gene Ontology (GO) enrichment analysis was performed separately for all DEGs identified in HeLa *DMD*ko+si*UTRN* cells and HSkM si*DMD*/*UTRN* myotubes (Fig. 8F). In HeLa cells, the enriched GO terms were dominated by categories related to type I interferon signalling, the antiviral innate immune response, complement activation, leukocyte chemotaxis and metal‑ion sequestration, together with processes linked to responses to xenobiotic and mechanical stimuli. In HSkM si*DMD*/*UTRN* myotubes, GO analysis instead highlighted terms associated with extracellular matrix organization and assembly, sarcomere organization, skeletal muscle cell differentiation, regulation of contraction, and structural or adhesive components of the cytoskeleton and ECM (including integrin binding and protein complexes involved in cell adhesion). Importantly, two of the identified enriched categories, regulation of vasoconstriction and sequestration of metal ions, were shared between the two cell models, whereas the other enriched categories were consistent with the functional annotation of genes identified as differentially expressed between the two models (Dataset S1).

Analysis of KEGG pathways in *DMD*ko+si*UTRN* HeLa cells revealed significant changes in the expression of genes related primarily to the cell cycle (Figs. 8G and S17), whereas in HSkM myotubes, the silencing of *DMD* and *UTRN* caused significant alterations in the expression of genes associated with several pathways, including those related to cytoskeleton organization, muscle contraction and motor proteins, receptor-extracellular matrix (ECM) and -cytokine interactions, and cardiomyopathy (Figs. 8G and S18–S25). Taken together, these transcriptomic changes correspond well to the phenotype observed in both dystrophin- and utrophin-deficient proliferating cells and myotubes.

## Discussion

To evaluate the role of different dystrophin isoforms and the compensatory role of utrophins in myoblasts and muscle fibres, we used the NTX-induced EDL muscle regeneration system, fibre-derived cell cultures, immortalized and primary cell lines, and RNA-sequencing analyses. We show that DP71 and DP427 play important roles in cell viability during proliferation and fibre differentiation, respectively. We further showed that utrophin can partially compensate not only for DP427 but also for DP71. These results (1) provide an explanation for the observed differences in disease severity among patients who differ in the presence of DP71 and (2) indicate that patients deficient in both DP71 and DP427 in skeletal muscle might require a different therapeutic approach than patients deficient in only DP427.

Individuals with distal mutations in exons 63–79 of the *DMD* gene, which abolish translation of DP71 in addition to DP427, DP260, DP140 and DP116, constitute approximately 10% of all DMD patients [4, 39]. Previous studies have shown that these patients have significantly lower stature and motor function than those with preserved DP71 translation [39, 40]. Studies in mice have only partially confirmed these results. On the one hand, dystrophin-null mice, which lack all the dystrophin isoforms, presented significantly more severe muscle disease than did *mdx* mice because they presented a decreased myofibre area, increased central nucleation, elevated fibrosis, fat accumulation, exacerbated calcification and altered infiltration patterns [41]. On the other hand, the grip strength and running time of the mice lacking DP427 and DP140 were similar to those of the mice lacking DP71, indicating that the absence of DP140, an isoform synthesized specifically in the brain and kidney, could be responsible for the observed phenotype [40, 42]. The discrepancies between the murine and human studies could be explained by differences in the genetic background of the mouse strains, the age of the analysed mice, or the species-dependent function of dystrophin in muscles and other tissues.

Because DP71 is ubiquitously synthesized throughout the body and constitutes the major isoform of dystrophin in the brain [2, 5, 43, 44], its absence in other tissues or cell types may indirectly affect muscle tissue performance. On the basis of experimental data, it has been speculated that the loss of DP71 or DP71/DP140 exacerbates muscle disease through alterations in the performance of lymphoblasts or brain cells, respectively [40, 41]. Our data on HEK293 and HeLa cells, as well as HeLa and HSkM cocultures, are consistent with this view; however, we also show that the lack of DP71 has a direct effect on muscle cells, as they show increased cyto- and genotoxicity and apoptosis. Previous data also indicated a role for DP71 in cell proliferation [7], with specific splice variants having opposing functions in the process [6]. While ectopic expression of DP71 in myotubes from *mdx* transgenic mice restored DGC, muscular dystrophy was not alleviated [9, 10]. It would be interesting to test whether the phenotype could be reduced by specific synthesis of DP71 in myoblasts/early myotubes deficient in all dystrophins.

PAX7 marks quiescent satellite cells, is maintained during proliferation, and is then lost during myogenic differentiation [45]. Our immunofluorescence microscopy analysis revealed threefold greater numbers of PAX7+ cells on DP427/utrophin-deficient myofibres than on wt myofibres; however, their myogenic potential was markedly lower than that of wt cells, as demonstrated in satellite cell-derived cultures. Their numbers also did not increase as significantly as those in wt muscles treated with NTX did. Similarly, previous studies reported increased numbers of PAX7+ cells in *mdx* mice and DMD patients and decreased myogenic potential of *mdx* and DMD myoblasts as well as CD133+ cells [46–51]. Since we did not observe DP427 in proliferating PAX7+ cells, it could be concluded that this phenotype is due to a secondary effect of the disease, as previously indicated [50]. However, DP427 was shown to be present in activated satellite cells, where its absence leads to defective asymmetric divisions and increased symmetric expansion [51]. Interestingly, we observed sevenfold and threefold greater numbers of PAX7+ cells on wt myofibres 12 and 24 days after NTX treatment, respectively. We hypothesize that this increase in PAX7+ cells after injury represents an intrinsic mechanism by which cells remain in an ‘alert state’ that allows for efficient and timely regeneration during the subsequent degenerative cycle. Since we observed an approximately 50% reduction in the number of BrdU+ nuclei in dko fibres 12 days after NTX treatment, we conclude that a large pool of PAX7+ cells in diseased muscles is in constant readiness to generate new fibres. Three days after NTX treatment, we observed faster dko fibre generation than wt fibres did, and a greater percentage of myogenin+ cells was observed in dko fibre-derived cultures at the earliest time point analysed, which supports this hypothesis. We also speculate that as the disease progresses, this protective mechanism ceases to function because of telomere shortening, as previously shown by Sacco et al. [52].

Our data indicate that the increased cyto- and genotoxicity, and apoptosis of DP71-deficient myoblasts, HEK, and HeLa cells result from membrane damage, increased intracellular calcium levels, mitochondrial aggregation, changes in the nuclear area, and elevated ROS. This phenotype may be a direct consequence of reduced DGC levels, as in DP427-deficient myotubes. Indeed, previous experimental data have shown that the absence of β-dystroglycan leads to an increased nuclear area in myoblasts [53, 54]. Although the senescence of β-dystroglycan-deficient myoblasts is controversial [53, 54], it should be noted that mice with conditional deletion of the dystroglycan (*Dag1*) gene in myotubes have a relatively mild phenotype, unlike chimeric ko mice, indicating the crucial role of DGC in the early stages of differentiation [55, 56]. Interestingly, we observed that β-dystroglycan is less dependent on DP71/DP427 levels than other DGC components are, and on the basis of these results, we conclude that the complex is assembled starting with dystroglycans, then dystrophins, and then other complex components, including sarcoglycans, syntrophins, and dystrobrevins.

Genotoxicity and structural changes in the nucleus could be directly linked to the presence of DGCs (or DG-like complexes) [54, 57]. However, we failed to detect significant levels of DP71 in this organelle. In contrast, we recorded its location in the cytoplasm and membrane. Indeed, previous results have shown that DP71 can form DGC at the membrane and that it can be found in the cytoplasm [9, 10, 57, 58]. The pathological phenotype was also much more pronounced in utrophin-deficient cells, indicating that, as in the case of DP427 [12, 19], UP395 can compensate for its absence. Some previous results have indicated that dystrophin and utrophin exclude their presence at the sarcolemma [59]. However, more recent data contradict this view [12, 60]. Our results also indicate that DP71 and UP395 can coexist in myoblasts and at early stages of muscle fibre differentiation.

Previous data linked myofibre death in DMD to contraction-induced muscle damage [61, 62], calcium influx through mechanosensitive channels [63], stretch-activated generation of ROS due to disorganized microtubules/NOX2 [63, 64], changes in signal transduction influenced in part by loss and mislocalization of sarcolemmal nNOS [2, 63, 65], and a persistent chronic immune response [66, 67]. Our results indicate that neither mechanical stress nor immune cell infiltration nor changes in NMJs, myotendinous junctions or other cell types are required for the pathological phenotype and death of myofibres [55, 68]. Furthermore, the observed increases in intracellular calcium, mitochondrial aggregation, and ROS production in proliferating cells and myotubes independent of dystrophin isoform loss may indicate that disease-related changes are only partially driven by nNOS- and microtubule-associated alterations because neither DP71 nor UP395 have nNOS binding sites, and DP427 and UP395 have different microtubule binding properties [8, 69]. Direct comparisons of wt and dko muscles also revealed no obvious changes in the plectin/desmin cytoskeleton when the regenerative state of muscle fibres was considered [17]. Our data indicate that loss of DP71/DP427 induces increased membrane permeability, influx of calcium ions, and mitochondrial aggregation and dysfunction, leading to increased cell death. Importantly, similar pathological changes were observed in nonmuscle dystrophin-deficient HEK293 and HeLa cells as well as in brain tissue [70].

In agreement with the observed phenotypic abnormalities, RNA-sequencing DEG analyses revealed profound transcriptomic changes in both dystrophin- and utrophin-deficient HeLa cells and HSkM myotubes related to oxidative stress, mitochondrial function, structural and signalling alterations and apoptosis. The analysis showing both increased and decreased gene expression suggests that cells attempt to compensate for the loss of dystrophin and utrophin, but this is accompanied by a cellular imbalance. The increased expression of some genes (e.g. *STAC3*, *GDF15*, *MCL1*) may reflect attempts to compensate for damage, such as increasing protection against oxidative stress, inhibiting apoptosis, or supporting muscle regeneration. Conversely, decreased expression of other genes (e.g. *ITGA6*, *ITGB1*, *SCD*) may result from disruptions in cell signalling, metabolism, or regeneration. This cellular response indicates that cells are trying to counteract the loss of dystrophin and utrophin but are unable to fully compensate, resulting in progressive pathology. *DMD*ko+si*UTRN* HeLa cells also revealed the downregulation of *PLK1*, *SKP2*, *MDM2*, and *APC/C*, which might impair mitotic progression and activate cell cycle arrest at the G1/S and G2/M phases, whereas the upregulation of *RAD21*, *PP2A*, *E2F*, and *MCM* may exacerbate replication stress and chromosomal instability. Consequently, these transcriptomic alterations might result in inhibited proliferation, accumulation of DNA damage, increased apoptosis, and errors in cell division. The loss of cytoskeletal proteins might also destabilize signalling between the cell membrane and the nucleus, further impairing repair and control mechanisms [71, 72].

Combined analysis of DEGs, GO terms, and KEGG pathways revealed that despite a number of similar gene expression and phenotypic changes, dystrophin and utrophin loss is generally interpreted differently in HeLa cells and differentiating HSkM myotubes, with a predominant effect on cell proliferation in HeLa cells compared with cytoskeletal reorganization in HSkM myotubes. In *DMD*ko+si*UTRN* HeLa cells, the coherent upregulation of interferon‑stimulated, complement and oxidative‑stress genes, together with enrichment for antiviral innate immune response, chemotaxis, metal‑ion sequestration and xenobiotic/stress response, points to the engagement of a chronic defence program that prioritizes survival and immune signalling over structural remodelling. In contrast, HSkM si*DMD*/*UTRN* myotubes downregulated many of these stress‑ and inflammation‑related genes and instead induced the expression of ECM, adhesion and differentiation markers, as reflected by GO enrichment for extracellular matrix organization, sarcomere assembly, skeletal muscle cell differentiation and cytoskeletal/adhesive functions. This pattern suggests that, in the myogenic context, the combined suppression of *DMD*/*UTRN* genes coincides with the resolution of early damage signals and the activation of a compensatory, partially functional structural maturation programme, rather than with the maintenance of a persistent stress state, as observed in HeLa cells. Taken together, the results of the phenotypic and RNA sequencing analyses point to both similar and distinct roles for DP71 and DP427 in cell proliferation and myotube differentiation, respectively.

While current therapeutic studies have focused on skeletal muscle therapy for DMD patients in the context of full-length dystrophin, DMD patients clearly require therapeutic intervention in other tissues, such as the heart and brain [2]. Nervous tissue can be difficult to treat owing to the large number of dystrophin isoforms and their splicing variants, which are present in a cell- and developmental time-dependent manner. Here, we show that the treatment of skeletal muscle may be more complex than previously thought and that 10% of DMD patients who also lack DP71 in addition to DP427 may require a specifically tailored combination therapeutic approach.

### Ethics approval and consent to participate

All methods were performed in accordance with the relevant guidelines and regulations. All animal experiments were performed in accordance with and approved by the Institutional Animal Care and Use Committee of the University of Washington under protocol 3333-01.

## Supplementary information

Supplementary Information

Dataset S1

Original Data

qPCR raw data

## Data Availability

The RNA-seq data from the HeLa cell lines are available at GEO accession GSE289357. The RNA-seq data from the HSkM cell lines are available at GEO accession GSE303672.
