# Supplementary material for: Dystrophins DP71 and DP427 determine cell viability during proliferation and myofibre differentiation
Source: Cell Death Dis. 2026 Apr 11;17(1):467. doi: 10.1038/s41419-026-08725-x (PMC13181019; doi:10.1038/s41419-026-08725-x)
Supplement: Supplementary file 1 — Supplementary Information [file 41419_2026_8725_MOESM1_ESM.docx]

**SUPPLEMENTARY INFORMATION**

**Dystrophins DP71 and DP427 determine cell viability during proliferation and myofibre differentiation**

**Running title:** Dystrophins in cell proliferation and differentiation

Sylwia Szwec^1^, Alicja Durska^1^, Paulina Kościelniak-Wawro^1^, Jeffrey S. Chamberlain^2,3,4,5^, Oleksandr Ostrovskyy^1^, Karolina Dominiak^1^, Solmaz Karimi^1^, Patryk Konieczny^1*^

^1^ Institute of Human Biology and Evolution, Faculty of Biology, Adam Mickiewicz University, ul. Uniwersytetu Poznańskiego 6, 61-614, Poznań, Poland.

^2^ Department of Neurology, University of Washington School of Medicine, Seattle, WA, 98109-8055, USA.

^3^ Senator Paul D. Wellstone Muscular Dystrophy Specialized Research Center, University of Washington School of Medicine, Seattle, WA, 98109-8055, USA.

^4^ Department of Biochemistry, University of Washington School of Medicine, Seattle, WA, 98109-8055, USA.

^5^ Department of Medicine, University of Washington School of Medicine, Seattle, WA, 98109-8055, USA.

* Correspondence: [patryk.konieczny@amu.edu.pl](mailto:patryk.konieczny@amu.edu.pl)

**Table S1: A list of primers, sgRNA and siRNAs used in the study.**

| **Target** | **Species** | **Forward** | **Reverse** |
| --- | --- | --- | --- |
| *RPLP0* | Human | CGTCCTCGTGGAAGTGACAT | CTTGGAGCCCACATTGTCTG |
| *DMD* | Human | GGACCAGCACAACCTCAAGCA | TCCTCCCTGTTCGTCCCGTATC |
| *UTRN* | Human | AAACTCCTCAGGCAGCACAA | GAGGCATCTGGATCAAGCGA |
| *DP427* | Human | CTGCACTAGGCTGAATGGGAA | CTCCGCCAGGAATGTTTTCAG |
| *DP71* | Human | GCCATGAGGGAACAGCTCAA | TTGCTTGAGGTTGTGCTGGT |
| *PAX7* | Human | CACTGTGCCCTCAGGTTTAGT | GTTCCGACTCCACATCCGAG |
| *MYOD* | Human | CGCAACGCCATCCGCTAT | TGTAGTCCATCATGCCGTCG |
| *MYOG* | Human | TGCCATCCAGTACATCGAGC | CAGATGATCCCCTGGGTTGG |
| *MYH1* | Human | AGGTCGCATCTCTACGCCA | CGCTCCCTTTCAGACTTTCG |
| *LAMA1* | Human | CACATCAGCACCAATGCCAC | ATTGGATGGCGTTCTCTGGG |
| *FAP* | Human | CATCTGGAAAAATGAAGACTTGGGT | CCAATAAGGCAAGCACAGCA |
| *SYNE1* | Human | GGTCCCGGTATAAAGGCTCG | CCCAGCCGCCCTCCT |
| *TGM2* | Human | ACAGGAGACCAAGAGACCAGA | TCTAAGACCAGCTCCTCGGC |
| *LTBP1* | Human | TGCACCCCCTGAAAAGGAAA | CAGGCACAGGAGGTGATGTT |
| *ABCB1* | Human | TGGCTACATGAGAGCGGAGG | TCCGTTGCACCTCTCTGGTC |
| *STAC3* | Human | TTTTTCCCCAGCACCACCAT | GTCCTCAAAGGGGAGGGTTC |
| *MYOM3* | Human | ACCCTGGATGAGATCTGGCT | GCATCCTCAAAAGCTTGCCC |
| *FOXO1* | Human | GAGGGTTAGTGAGCAGGTTACA | ACTGCTTCTCTCAGTTCCTGC |
| *DRAM1* | Human | TCAACCCCTTCCTCCCGTA | TCGTGGCTGCACCAAGAAA |
| *RYR1* | Human | GGGTGAAGTTCCTGGTCTCAG | TCTCATCCTCATCGCCCTCT |
| *FOS* | Human | GGGGCAAGGTGGAACAGTTA | AGTTGGTCTGTCTCCGCTTG |
| *mtDNA_*  *ND1* | Human | TACGGGCTACTACAACCCTTC | ATGGTAGATGTGGCGGGTTT |
| *gDNA_*  *SERPINA* | Human | CAGTGAATAAATGAGGCGTACATCC | GACTGTTTCTCATGCCTCTGGAAAG |
| *CRISP_*  *DMD* | Human | GAGAGTGTGGTTCACGTTTGG | AGGGCCCTGTTGTAATGCTAA |
| *Rplp0* | Mouse | GCTTTGGGCATCACCACGAA | GTTGCGGACACCCTCCAGAA |
| *Dmd* | Mouse | GCTTCCTTTGGGGGCAGTAA | CACTTGGCTTGATGCTTGGC |
| *Utrn* | Mouse | CTGACTCTGACTCCCGCATC | GTCCGTGCTAGTGTCGTGAG |
| *Dp427* | Mouse | CTCCACCCTCAGCACAAGAG | TCATGCCAACATGCCCAAAC |
| *Dp71* | Mouse | ATGAGGGAACAGCTCAAAGG | TGCAGCTGACAGGCTCAAGA |
| *Pax7* | Mouse | CTGCTGAAGGACGGTCACTG | ATCCAGACGGTTCCCTTTGT |
| *Myod* | Mouse | GCTACGACACCGCCTACTAC | GGTGGTGCATCTGCCAAAAG |
| *Myog* | Mouse | AATGCACTGGAGTTCGGTCC | CACCCAGCCTGACAGACAAT |
| *Myh1* | Mouse | TCCCTAAAGGCAGGCTCTCTC | AACACCGATGACTTGGCGT |
| *mtDNA_Nd1* | Mouse | CTAGCAGAAACAAACCGGGC | CCGGCTGCGTATTCTACGTT |
| *gDNA_*  *Hk2* | Mouse | GCCAGCCTCTCCTGATTTTAGTGT | GGGAACACAAAAGACCTCTTCTGG |

| **Name** | **Species** | **Sequence** |
| --- | --- | --- |
| **sgRNA** | | |
| *DMD* ex65 | Human | GACCACUAUUUAUGACCGCC |
| **siRNA** | | |
| *DMD*/*Dmd* (s501299) | Human, mouse | AAACAAAUUUCGAACCAAAtt |
| *UTRN* (s553059) | Human | GGAAUAUUGUAUACCUACAtt |
| *Utrn* | Mouse | CGAAUGAAGUUUUCAAGCAtt |

**Table S2: A list of antibodies used in the study.**

| **Antibody** | **Species** | **Concentration** | | **Manufacturer** |
| --- | --- | --- | --- | --- |
|  |  | **WB** | **IF** |  |
| α-Actinin | Mouse | 1:1000 |  | Sigma (A7811) |
| ATP synthase α (7H10) | Mouse | 1:500 (muscle) |  | Invitrogen (A21350) |
| ATP Synthase β (3D5AB1) | Mouse | 1:1000 | 1:200 | Thermo Fisher (A21351) |
| BrdU (BU 1/75) | Rat |  | 1:50 | Novus Biologicals (NB500-169) |
| Caveolin 3 | Rabbit | 1:2000 | 1:500 | Abcam, ab2912 |
| Desmin (Clone D33) | Mouse | 1:750 | 1:100 | Agilent Dako (M076029) |
| Pan α-Dystrobrevin | Mouse | 1:1000 | 1:100 | BD Tranduction Laboratories (610766) |
| β-Dystroglycan (43DAG1/8D5) | Mouse | 1:1000 | 1:10-1:50 | Abcam (ab49515), Novocastra (NCL-b-DG) |
| Dystrophin (MANDRA1) | Mouse | 1:2000 | 1:100 | Sigma–Aldrich (ab7164);  Santa Cruz Biotechnology (sc-73592) |
| Dystrophin | Rabbit | 1:1000 | 1:50 | Abcam (ab15277) |
| Dystrophin | Rabbit | 1:2000 | 1:200 | Proteintech (12715-1-AP) |
| Dytrophin 303-3  (N-terminus) | Rabbit |  | 1:600 | J. S. Chamberlain |
| ERTR7 | Rat |  | 1:300 | Acris |
| Lamin B1 | Rabbit |  | 1:500 | Abcam (ab16048) |
| MyoD (M-318) | Rabbit |  | 1:100 | Santa Cruz (sc-760) |
| Myogenin (F5D) | Mouse |  | 1:20 | DSHB |
| Myosin (B-5) | Mouse | 1:1000 | 1:50 | Santa Cruz Biotechnology (sc-376157) |
| Myosin (MF-20) | Mouse |  | 1:20 | DSHB |
| Anti-Pax7  (concentrate or supernatant) | Mouse |  | 1:10 (conc.) or undiluted (supern.) | DSHB |
| Phalloidin-iFluor 647 reagent |  |  | 1:1000 | Abcam (ab176759) |
| Plectin | Rabbit | 1:1000 |  | Abcam (ab83497) |
| Plectin #46 | Rabbit |  | 1:100 | provided by G. Wiche |
| Plectin 1 | Rabbit | 1:2000 | 1:500 | provided by G. Wiche |
| Plectin 1f | Rabbit | 1:200 | 1:50 | Provided by G. Wiche |
| β-Sarcoglycan (BSarc/5B1) | Mouse | 1:200 |  | Novocastra |
| γ-sarcoglycan (35DAG/21B5) | Mouse | 1:500 | 1:100 | Novocastra |
| α1-syntrophin (1351) | Mouse |  | 1:1000 | provided by S. Froehner |
| α1-syntrophin (SYN17) | Rabbit | 1:2000 |  | provided by S. Froehner |
| α-Tubulin | Mouse | 1:500 |  | Sigma (T6074) |
| α-Tubulin (DM1A) | Mouse | 1:2000 | 1:500 | Abcam (ab7291) |
| Utrophin (MANCHO3) | Mouse | 1:200 | 1:100 | Developmental Studies Hybridoma Bank (8A4) |
| Utrophin (MANCHO7) | Mouse | 1:500 | 1:50 | Santa Cruz Biotechnology (sc-81557) |
| Utrophin (UTR316) | Rabbit | 1:1000 | 1:500 | provided by S. Froehner |
| VDAC1/Porin | Rabbit | 1:500 (muscle) |  | Abcam (ab15895) |
| VDAC1/Porin | Rabbit | 1:1000 | 1:100 | Sigma-Aldrich (SAB5700655) |
| Vimentin | Mouse |  | 1:500 | Abcam (ab20346) |
| Alexa 488 anti-rabbit | Goat |  | 1:1000 | Molecular Probes |
| Alexa 488 anti-rabbit IgG H&L | Donkey |  | 1:400 | Abcam (ab150073) |
| Alexa 488 anti-rat | Goat |  | 1:1000 | Molecular Probes |
| Alexa 594 anti-mouse | Goat |  | 1:1000 | Molecular Probes |
| Alexa 488, streptavidin |  |  | 1:1000 | Molecular Probes |
| Alexa 594, streptavidin |  |  | 1:500 | Molecular Probes |
| Cy5 anti–rabbit | Goat |  | 1:1000 | Jackson Immuno Research |
| Rhodamine (TRITC) anti-mouse IgG (H+L) | Donkey |  | 1:400 | Jackson Immuno Research |
| anti-mouse IgG (Fab specific)–peroxidase antibody | Goat | 1:10 000 |  | Sigma–Aldrich (A9917) |
| anti-rabbit IgG | Goat | 1:10 000 |  | Abcam (ab97051) |
| anti–mouse HRP-conjugated IgGs | Donkey | 1:20 000 |  | Jackson Immuno Research |
| anti–rabbit HRP-conjugated IgGs | Donkey | 1:20 000 |  | Jackson Immuno Research, |

**
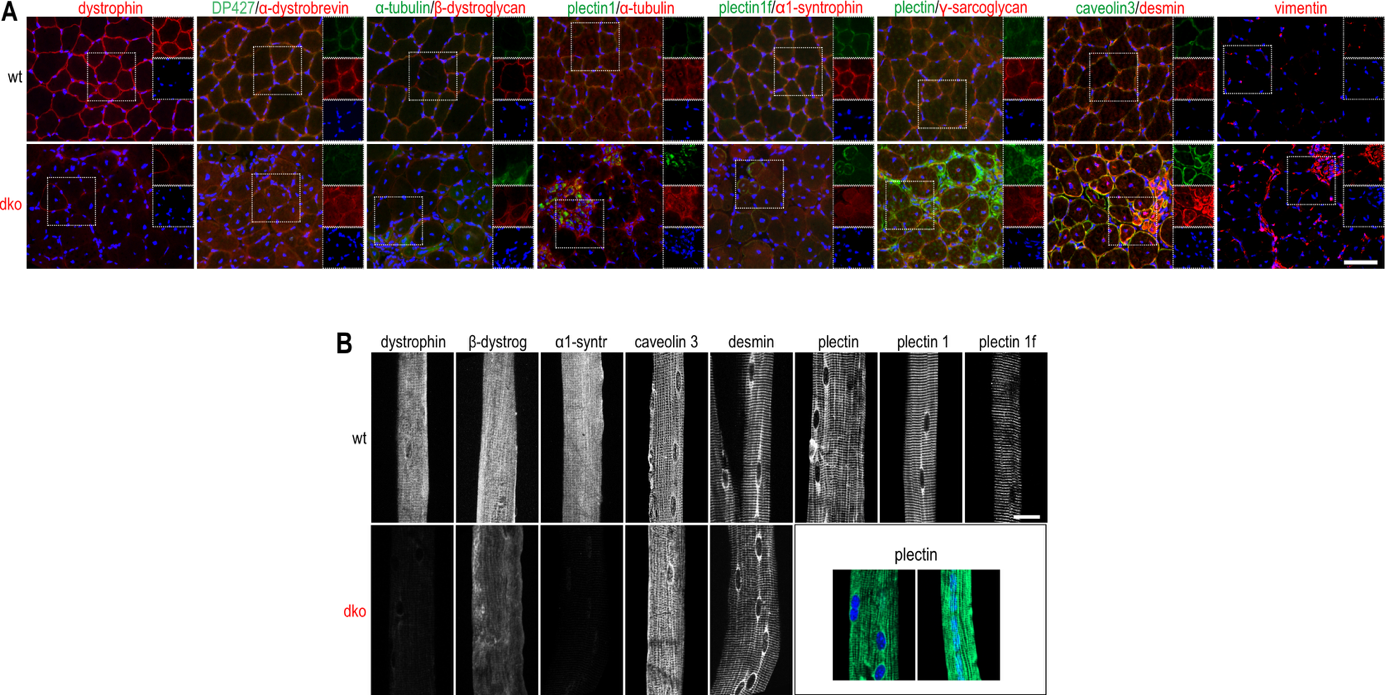
**

**Figure S1: Loss of dystrophin DP427 and utrophin in skeletal muscle leads to proteomic changes.**

**A** Frozen sections of EDL muscles from wt and dko mice immunostained for costameric and cytoskeletal proteins and nuclei (blue channel, DAPI). The insets show separate red, green and blue channels. Bar, 50 μm. **B** Single fibres from wt and dko EDL muscles immunostained for costameric and cytoskeletal proteins. The inset shows two dko fibres differing in their regenerative state, immunolabelled with anti-plectin antibody and DAPI. Bars, 50 μm (**A**) and 20 μm (**B**).

**
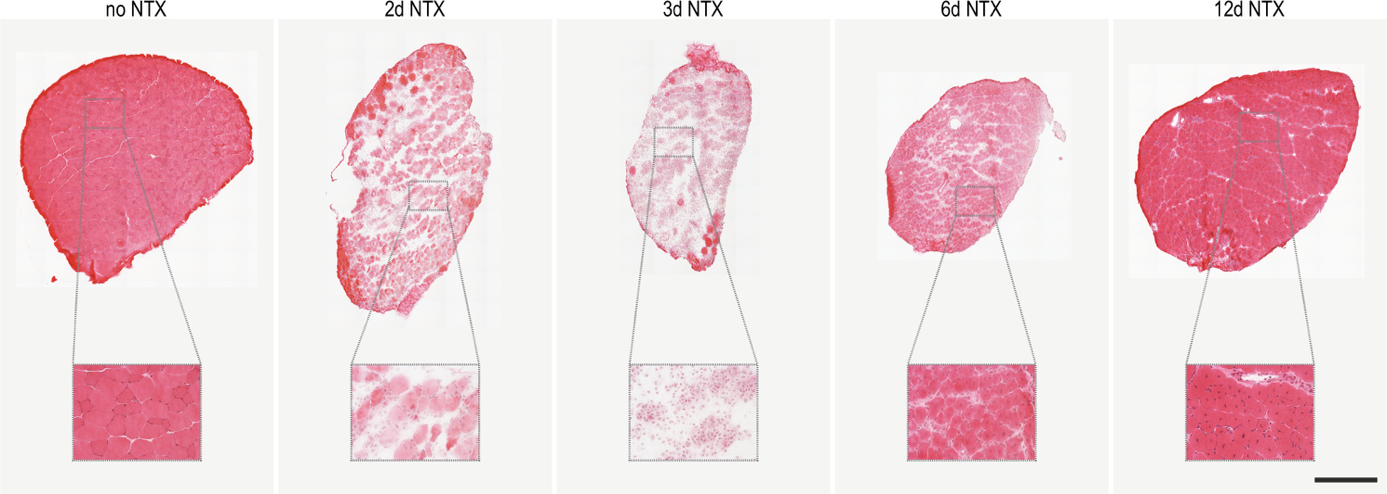
**

**Figure S2: Notexin (NTX) induces effective degeneration of muscle fibres and their subsequent regeneration from activated muscle stem cells.**

Composite images of sections from either uninjected (no NTX) or NTX-treated EDL muscles. Wt muscles were collected at various timepoints after NTX injection to assess the efficiency of NTX-induced muscle damage and subsequent regeneration and maturation of myofibres. Two days after NTX injection, massive myofibre death and infiltration of immune system cells were evident. By day 3, most of the dead fibres had been removed, and the muscles were filled with mononuclear cells. Six days post-NTX, *de novo*-formed fibres were apparent, which then increased in size 12 days post-NTX. Bar, 500 μm.

**
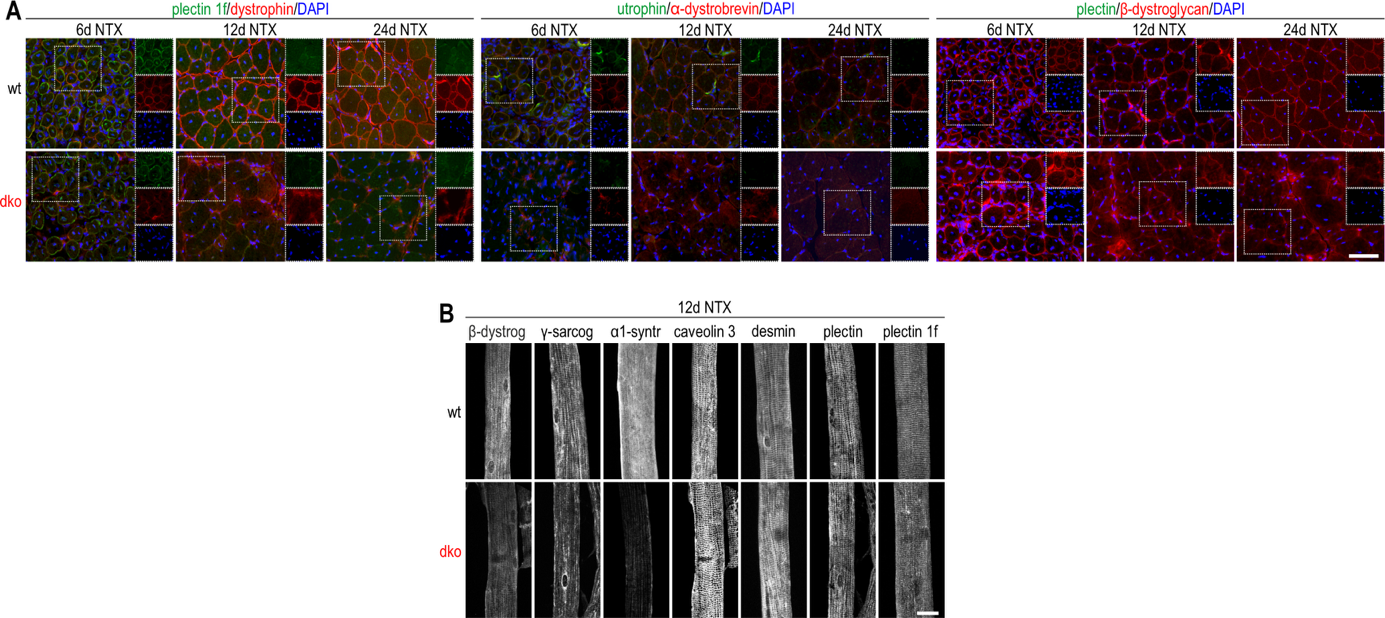
**

**Figure S3: NTX-induced regeneration reveals complementary expression of the *DMD* and *UTRN* genes and an increase in DGC proteins during fibre differentiation.**

**A** Frozen sections of EDL muscles isolated from wt and dko mice at 6, 12 and 24 days after NTX injection were immunostained for costameric and cytoskeletal proteins and nuclei (DAPI). The insets show separate red, green and blue channels. **B** Single fibres from wt and dko EDL muscles harvested 12 days after NTX treatment were immunostained for costameric and cytoskeletal proteins. Bars, 50 μm (**A**) and 20 μm (**B**).

**
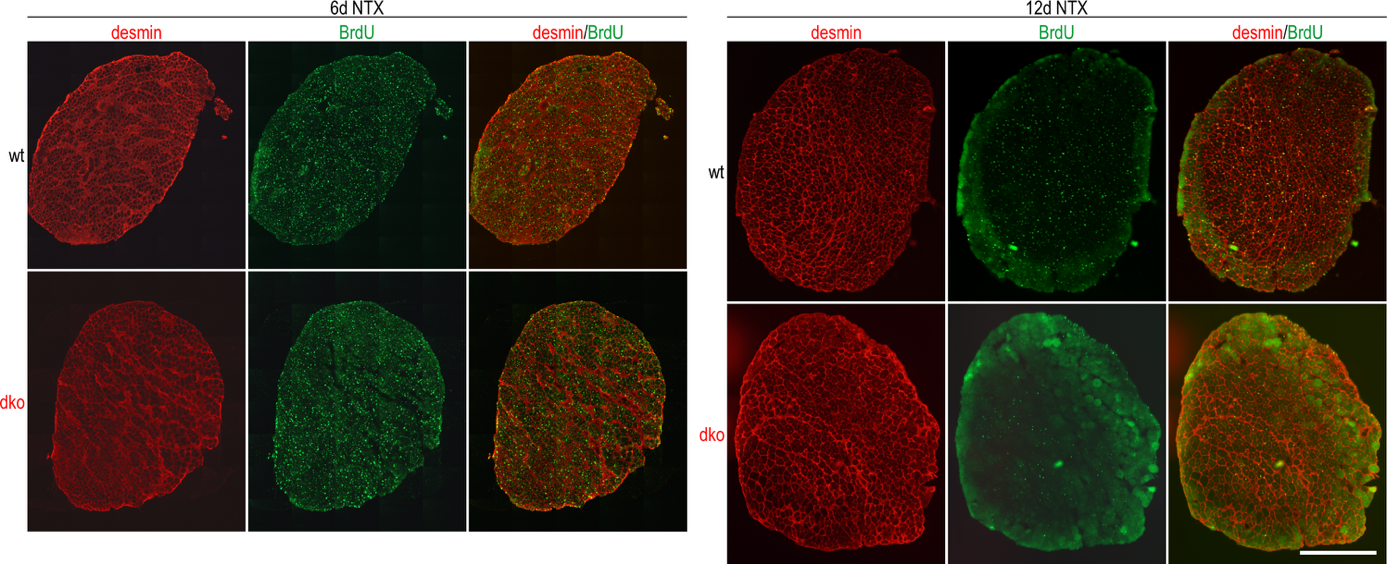
**

**Figure S4: BrdU injections into wt and dko mice after NTX treatment reveal lower lifespans of dko myofibres.**

BrdU was injected subcutaneously 2 and 3 days after EDL NTX injection. The muscles were isolated 6 and 12 days after NTX administration. Bar, 500 μm.

**
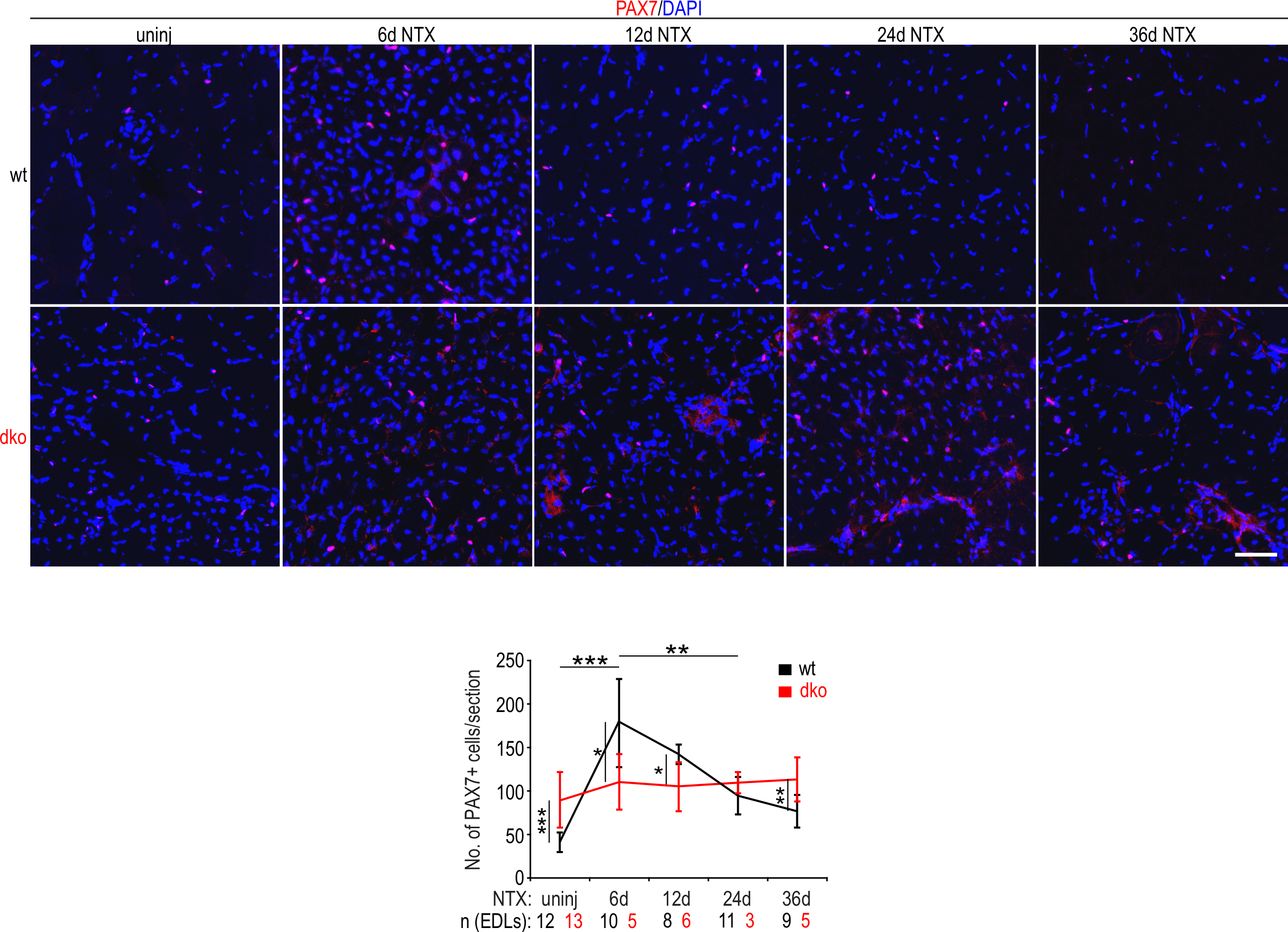
**

**Figure S5: NTX treatment reveals the high myogenic potential of wt EDL muscles.**

Frozen sections from wt and dko EDL muscles immunolabelled for PAX7 and nuclei (DAPI) and quantification of the numbers of PAX7+ nuclei in uninjected and NTX-treated wt and dko EDL muscle sections. The results are shown as the mean values ± SDs. n, number of biological replicates (EDL muscles). n, number of biological replicates. Bar, 50 μm. *, p < 0.05; **, p < 0.01; ***, p < 0.001 (t-test).

**
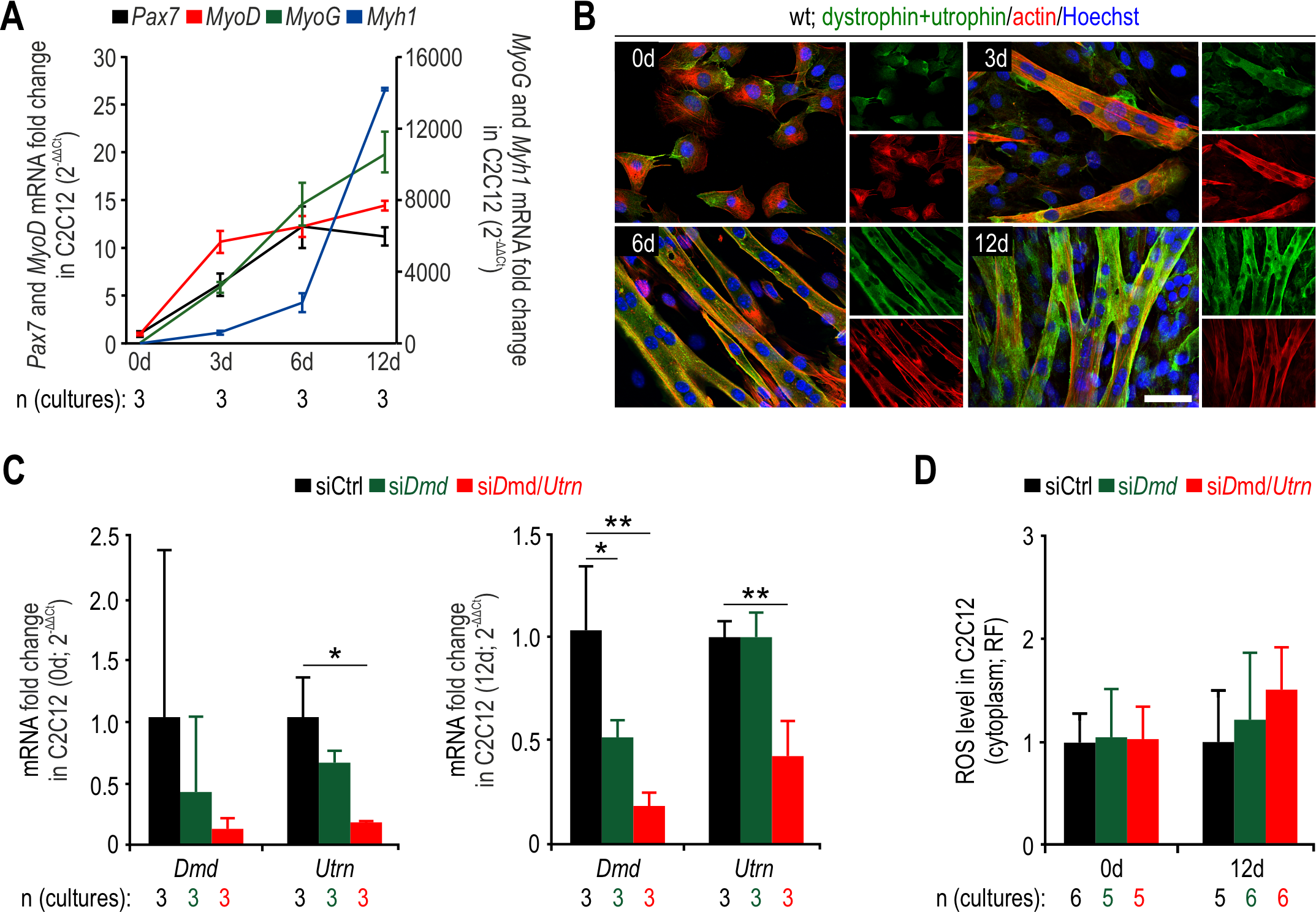
**

**Figure S6: Dystrophin and utrophin are synthesized at various stages of myogenic differentiation.**

**A** RT‒qPCR analyses of myofibre differentiation markers (*Pax7, MyoD, MyoG*, and *Myh1*) in the C2C12 cell line at different time points during differentiation. **B** Images of C2C12 myoblasts and myotubes immunolabelled for dystrophin and utrophin (Proteintech antibody, see also Fig. S7), actin and nuclei (Hoechst). Bar, 50 μm. **C** RT‒qPCR analysis of *Dmd* and *Utrn* knockdown efficiency via siRNA in proliferating (0d) and differentiated (12d) C2C12 cells. **D** ROS production in dystrophin-, and dystrophin- and utrophin-deficient C2C12 cells. The results in **A** are shown as the mean values ± SDs and those in **C**, **D** as the mean values + SDs. Bar in **B**, 50 μm. RF, relative fluorescence; n, number of biological replicates. *, p < 0.05; **, p < 0.01 (t-test).

**
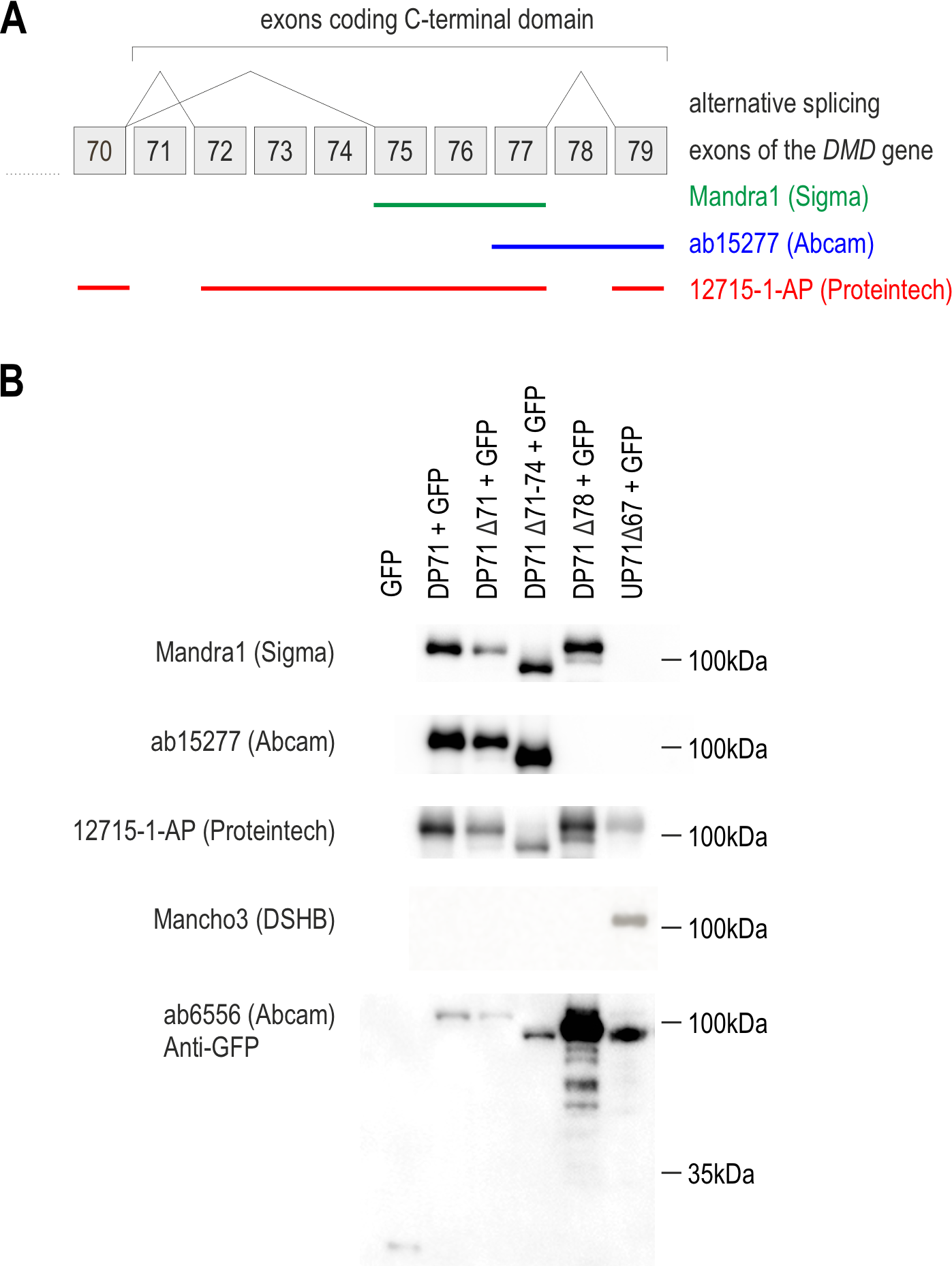
**

**Figure S7: Comparison of the specificity of commercially available antibodies against dystrophin and utrophin.**

**A** Dystrophin protein regions used as immunogens to obtain commercially available dystrophin antibodies. **B** A collection of vectors encoding GFP and GFP fusion proteins with different splicing variants of the dystrophin isoform DP71 or the utrophin UP71 were used to transfect the *DMD* ko HEK293 cell line and prepare protein extracts for testing the specificity of various commercially available antibodies against dystrophin and utrophin via Western blot analysis. Notably, that whereas the dystrophin antibody 12715-1-AP (Proteintech) also recognizes utrophin, it detects all dystrophin isoforms and is most reliable for cell immunofluorescence and immunoblotting studies.

**
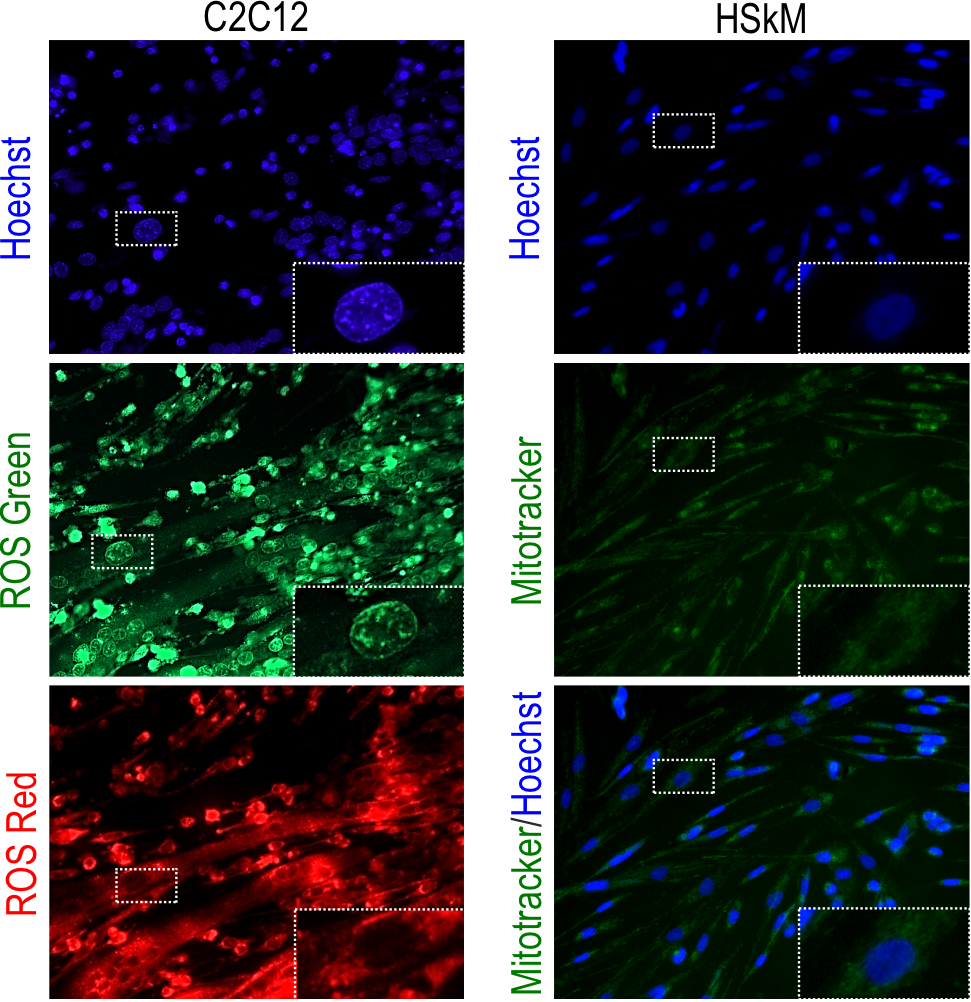
**

**Figure S8: Microscopic validation of reagents for TECAN-based quantification of ROS production and mitochondrial content.**

**
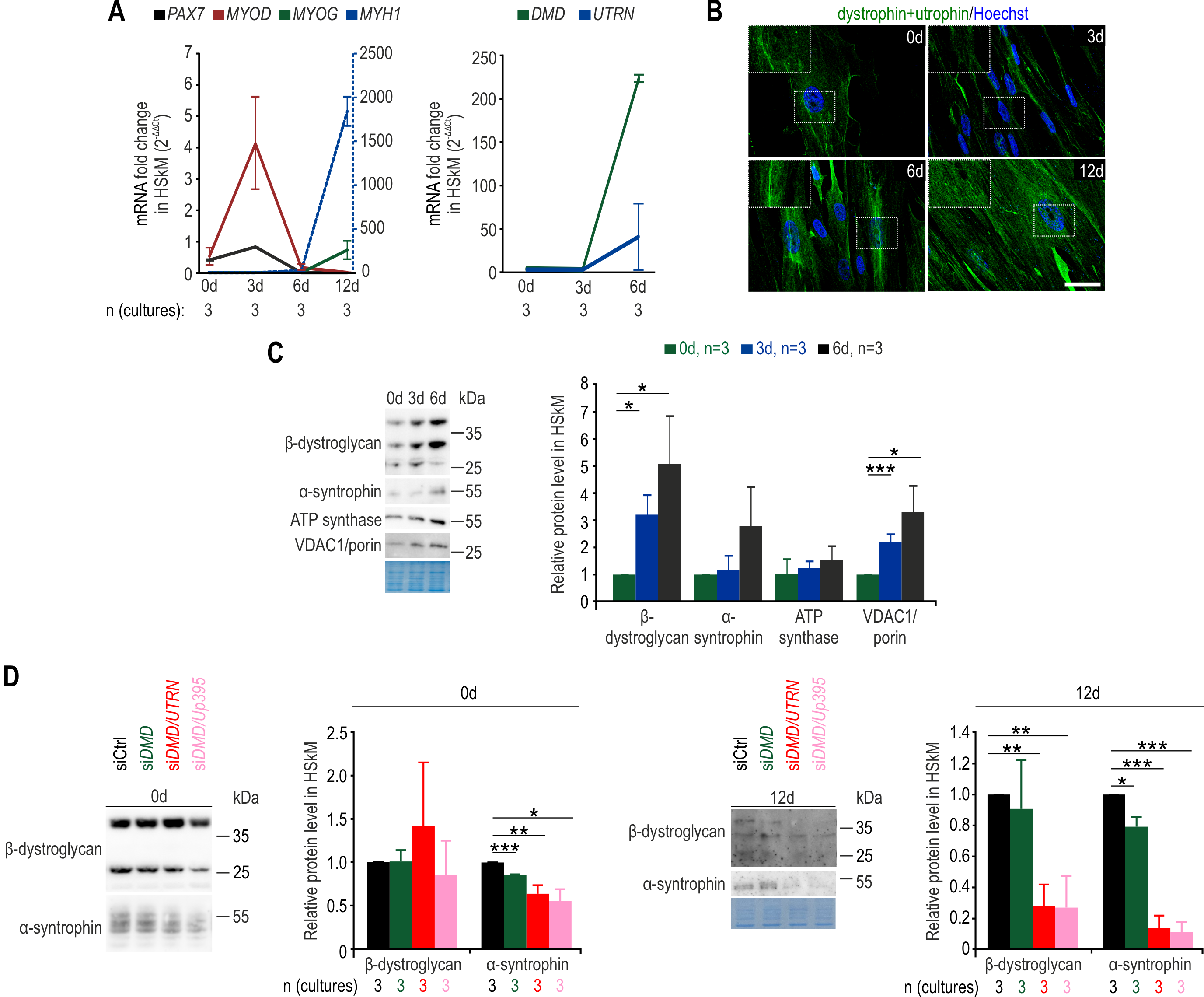
**

**Figure S9: Downregulation of *DMD* and *UTRN* genes in proliferating and differentiated HSkM cells leads to a reduction in DGC proteins.**

**A** RT‒qPCR analysis of myofibre differentiation markers (*PAX7, MYOD, MYOG*, and *MYH1*) along with the *DMD* and *UTRN* mRNAs in the HSkM cell line at different time points during differentiation. **B** Immunostaining for dystrophin/utrophin (Proteintech antibody, see also Fig. S7) and nuclei (blue channel; Hoechst) in HSkM cells at 0, 3, 6 and 12 days of differentiation. The insets show separate green channels for dystrophin/utrophin labelling. **C** Immunoblotting analysis of HSkM protein extracts from days 0, 3 and 6 of differentiation, with densitometric evaluation of the band intensities relative to the HSkM levels on day 0. Coomassie-stained polyacrylamide gels were used for normalization. **D** Immunoblot analysis of β-dystroglycan and α-syntrophin levels in HSkM myoblasts and myotubes after siRNA transfection, with densitometric evaluation of band intensities relative to wt levels. Coomassie-stained polyacrylamide gels (for d0, see Fig. 5B) were used for normalization. Bar in **B**, 100 μm. The results in **A** are shown as the mean values ± SDs and those in **C**, **D** as the mean values + SDs. n, number of biological replicates. *, p < 0.05; **, p < 0.01; ***, p < 0.001 (t-test).

**
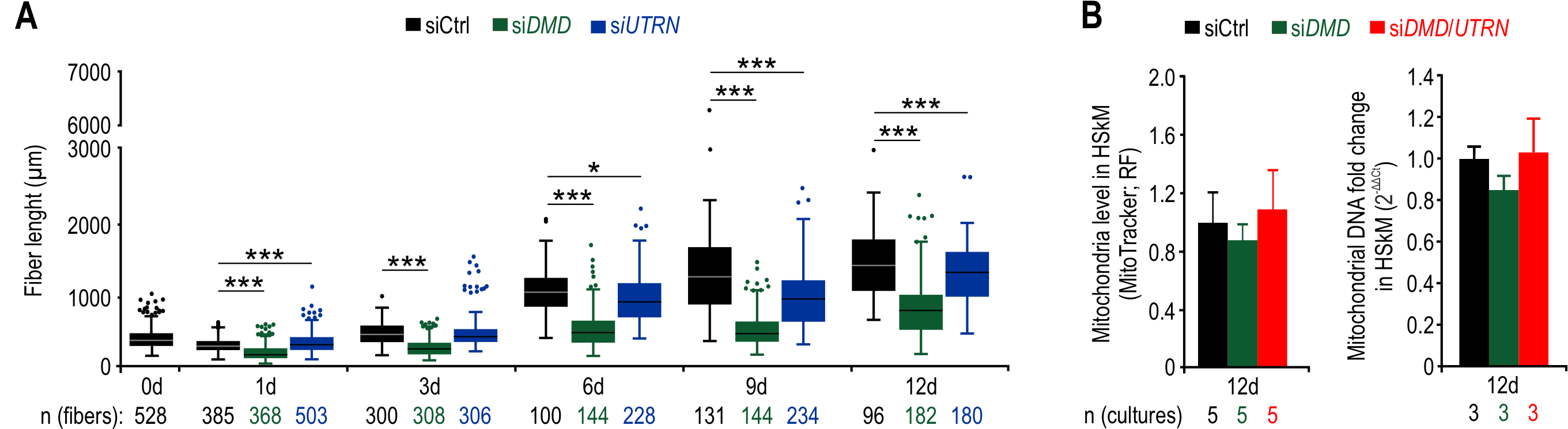
**

**Figure S10: Dystrophin and utrophin deficiency affects fibre length in HSkM myotubes.**

**A** Quantification of HSkM fibre lengths during differentiation after *DMD* and *UTRN* gene expression was silenced. **B** Analysis of mitochondrial contents on day 12 of HSkM differentiation after siRNA transfection, based on the MitoTracker levels and the mtDNA/gDNA ratio. The results in **A** are shown as the mean values ± SDs and in **B** as the mean values + SDs. RF, relative fluorescence; n, number of biological replicates. *, p < 0.05; ***, p < 0.001 (z-test in **A** and t-test in **B**).

**
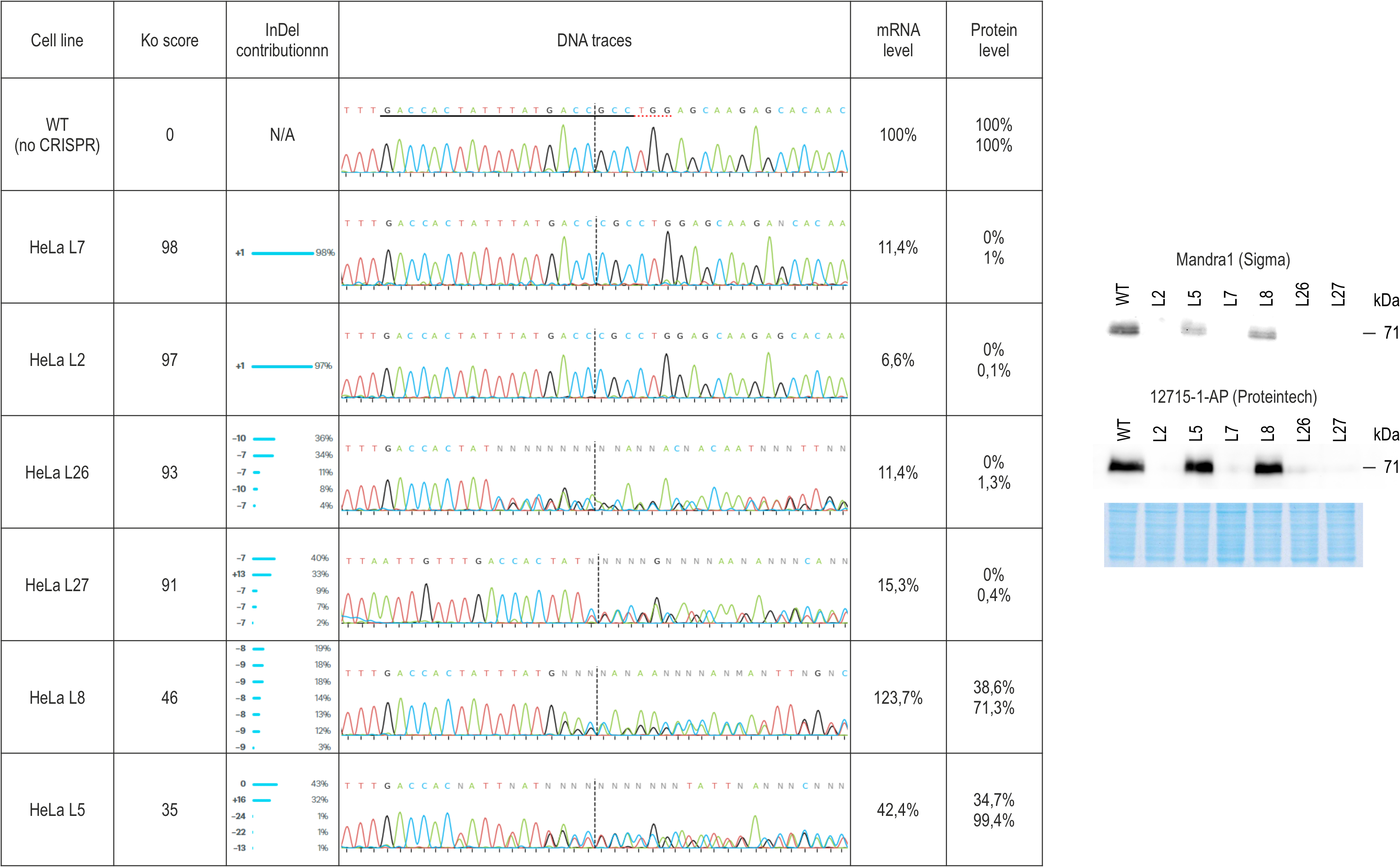
**

**Figure S11: Characterization of CRISPR/Cas9 *DMD* ko HeLa cell lines.**

Knockout (Ko) scores, InDel distributions and DNA traces were obtained after Sanger sequencing and data analyses with the Synthego ICE Analysis Tool. Transcript (mRNA) levels were determined via RT‒qPCRs. Protein levels were calculated via immunoblot analysis with Mandra 1 and 12715-1-AP Proteintech antibodies. The coomassie-stained polyacrylamide gel fragment shows equal sample loading. The order of the *DMD* ko cell HeLa lines is based on the ko score.

**
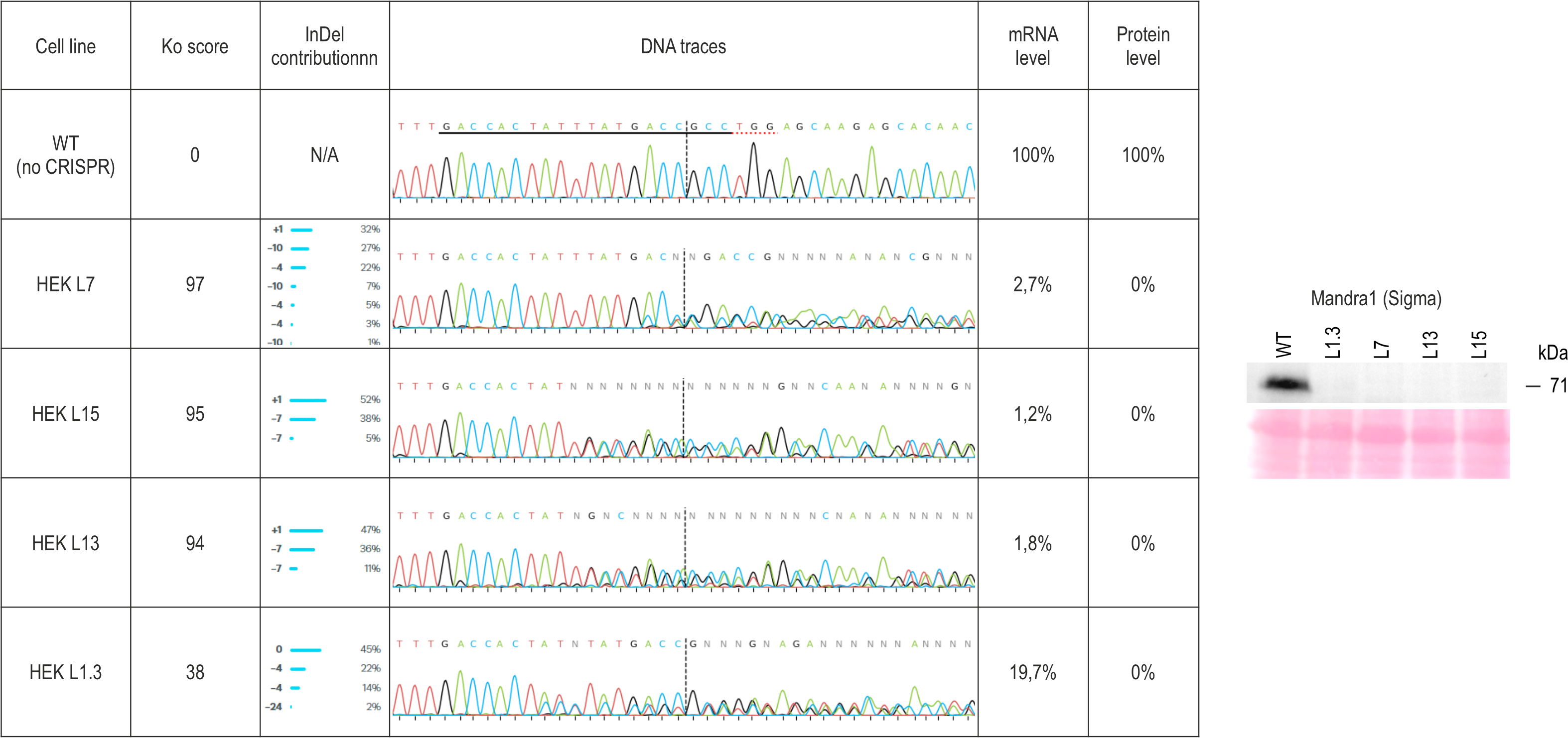
**

**Figure S12: Characterization of the CRISPR/Cas9 *DMD* ko HEK293 cell line.**

Ko scores, InDel distributions and DNA traces were obtained after Sanger sequencing and data analyses with the Synthego ICE Analysis Tool. Transcript (mRNA) levels were were determined via RT‒qPCRs. Protein levels were calculated via immunoblot analysis with Mandra 1 antibody. The Ponceau-labelled membrane fragment shows equal sample loading. The order of *DMD* ko cell HEK293 lines is based on the ko score.

**
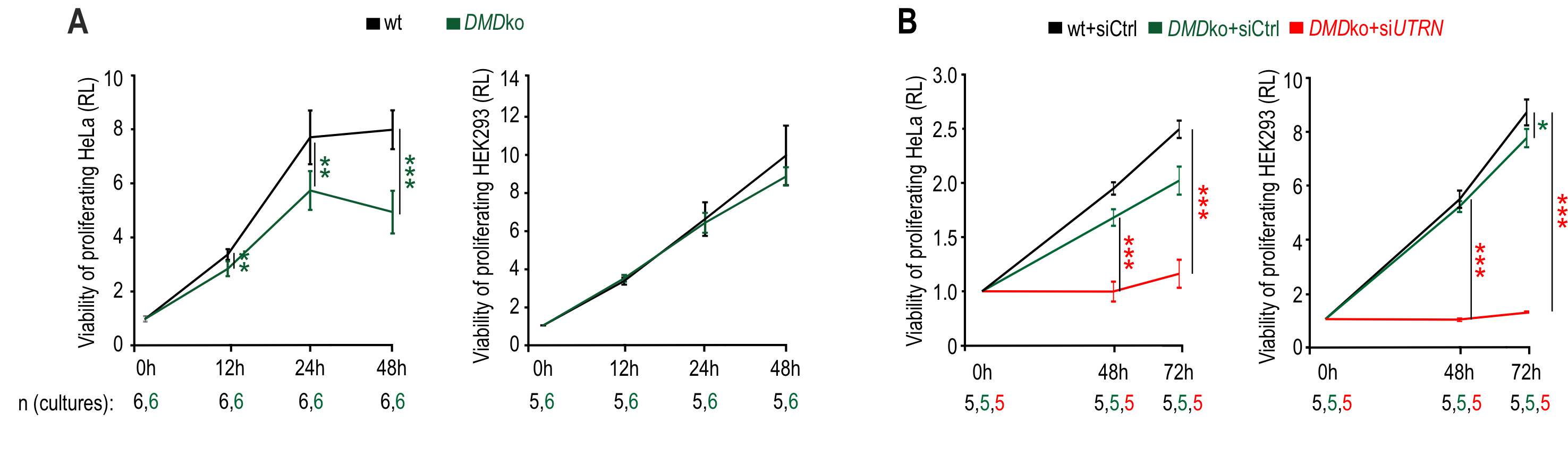
**

**Figure S13: Utrophin deficiency in *DMD* ko HeLa and HEK293 cells suppresses cell proliferation.**

**A**, **B** Assessment of the viability of proliferating wt and *DMD* ko HeLa and HEK293 cells for 48 hours (**A**) and wt and *DMD* ko HeLa and HEK293 cells for 72 hours after siRNA treatment (**B**). The results are shown as the mean values ± SDs. RL, relative luminescence; n, number of biological replicates. *, p < 0.05; **, p < 0.01; ***, p < 0.001 (t-test).

**
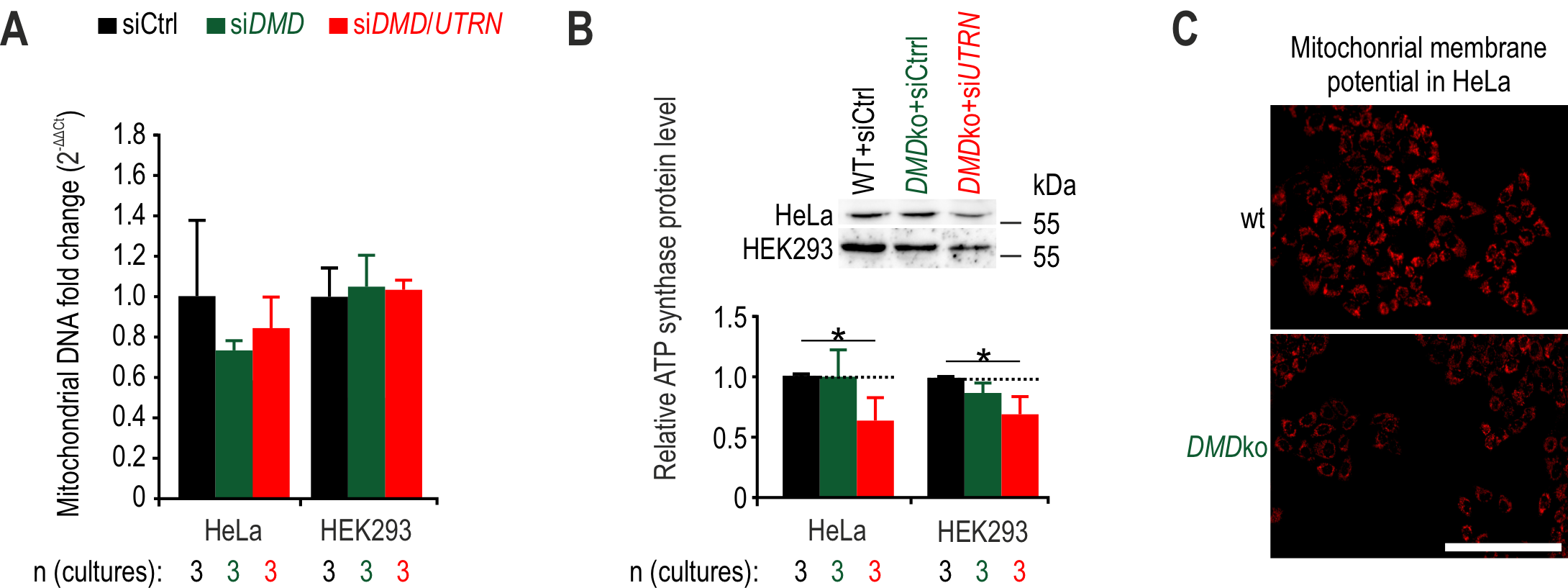
**

**Figure S14: *DMD* ko HeLa cells show reduced plasma membrane potential.**

**A** Mitochondrial DNA analysis by qPCR in wt HeLa and HEK293 cells after *DMD* or *DMD*/*UTRN* downregulation. **B** Immunoblot analysis of protein extracts from wt and *DMD* ko HeLa and HEK293 cells after siRNA transfection, with densitometric evaluation of band intensities normalized to wt levels. Coomassie-stained polyacrylamide gels from Fig. S13B were used for normalization. **C** Images of wt and *DMD* ko HeLa cells stained for mitochondrial membrane potential. Bar, 100 μm. The results are shown as the mean values + SDs. n, the number of biological replicates. *, p < 0.05 (t-test).

**
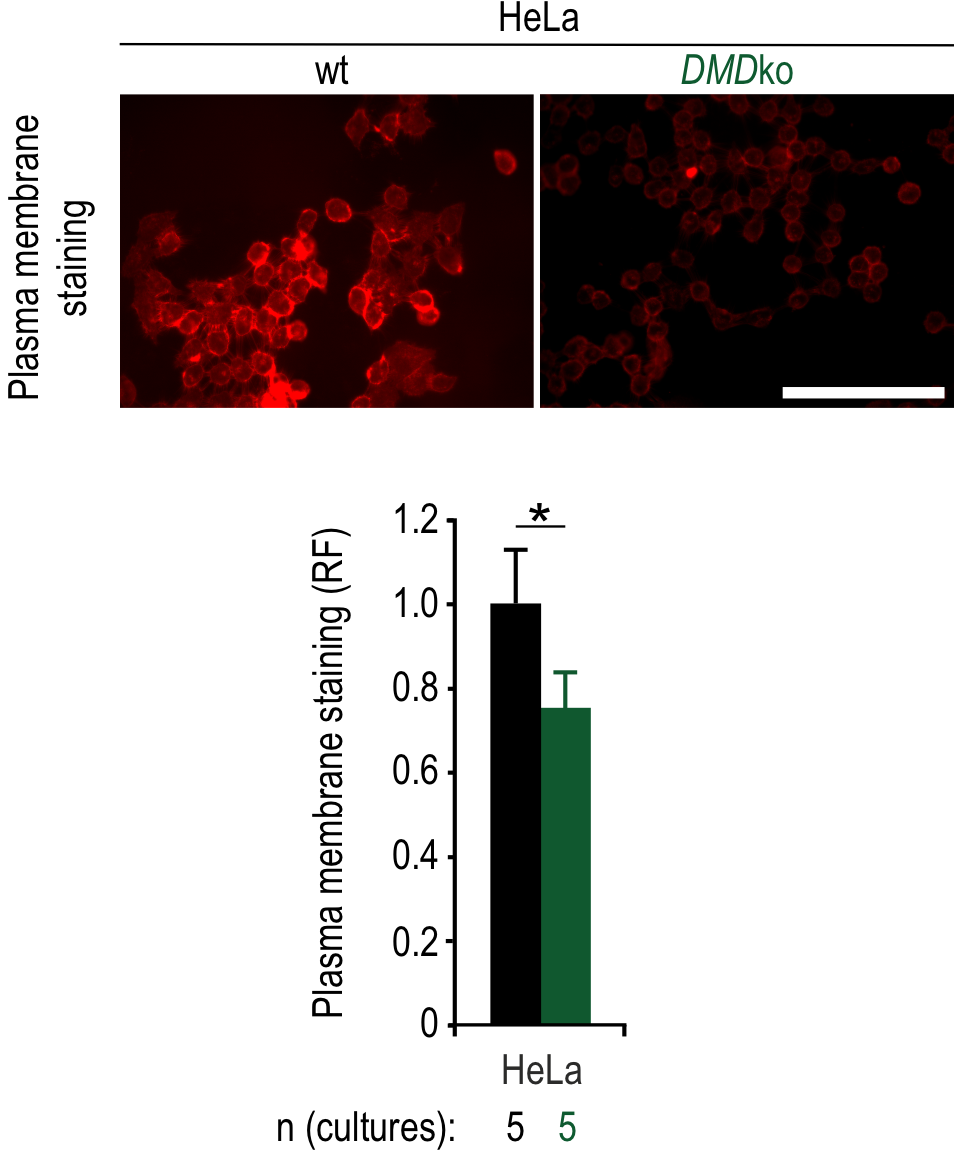
**

**Figure S15: *DMD* ko HeLa cells show reduced plasma membrane staining.**

Images of wt and *DMD* ko HeLa cells stained for the plasma membrane and quantification of the plasma membrane staining via a Tecan SPARK plate reader. Bar, 100 μm. The results are shown as the mean values + SDs. RF, relative fluorescence; n, number of biological replicates. *, p < 0.05 (t-test).

**
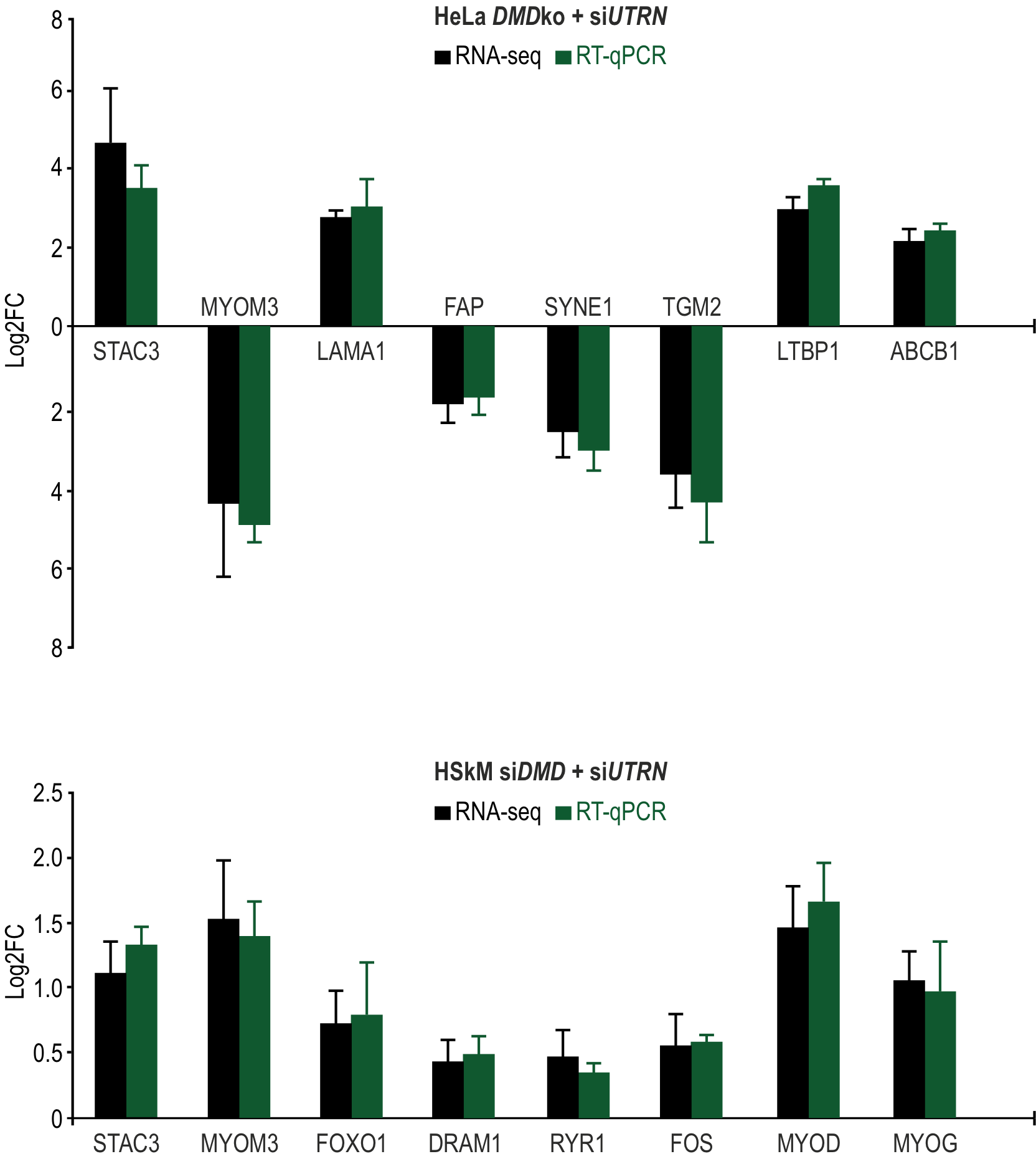
**

**Figure S16: Validation of RNA sequencing results.**

Gene expression changes in *DMD*ko + si*UTRN* HeLa cells and si*DMD* + si*UTRN* HSkM myotubes, comparing log2FC values obtained from RNA sequencing with those measured by RT‒qPCR relative to their respective controls. The results are shown as the mean values + SDs.

**
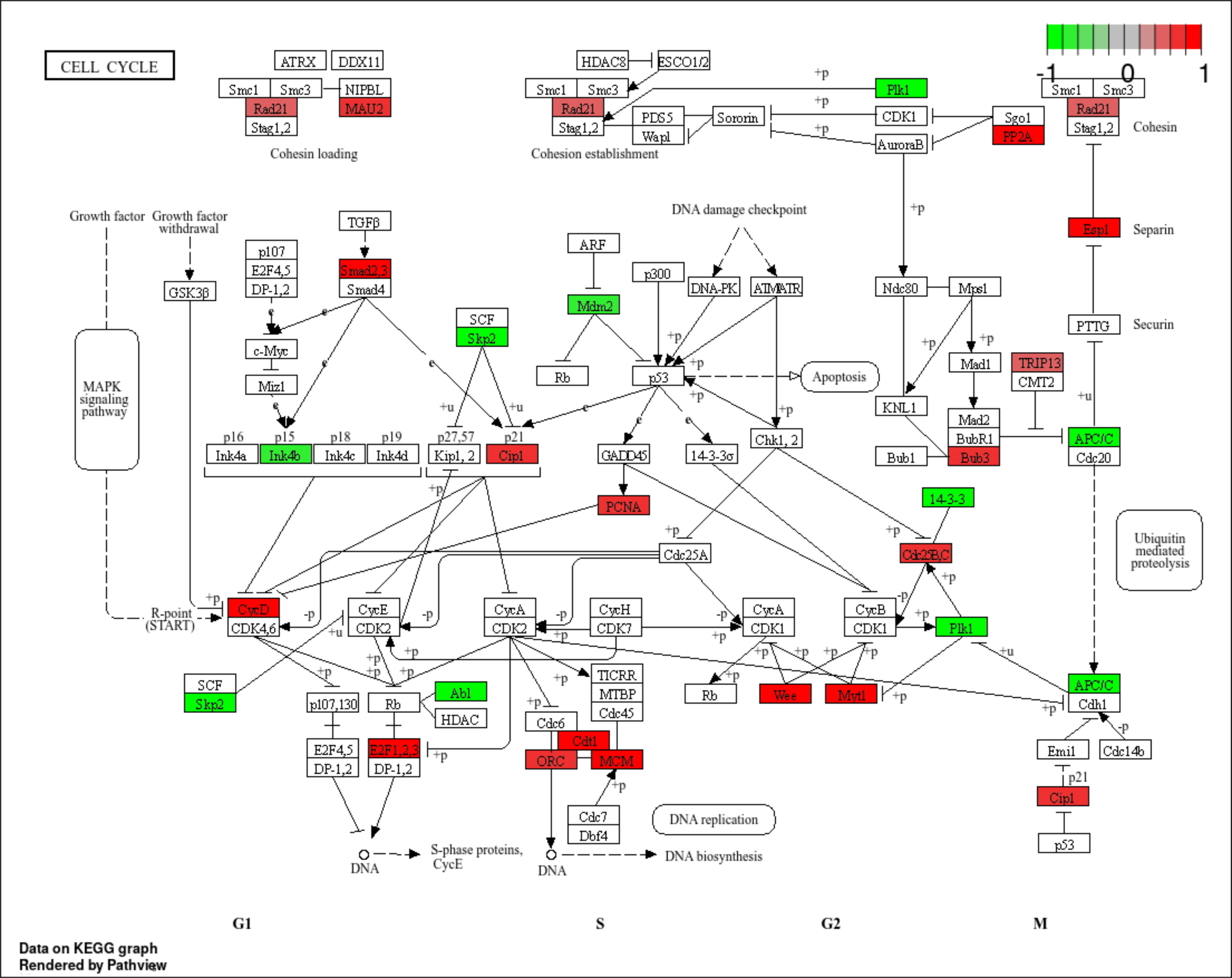
**

**Figure S17: Schematic of the cell cycle metabolic pathway in dystrophin- and utrophin-deficient HeLa cells.**

The image illustrates the cell cycle metabolic pathway identified in the KEGG analysis. The schematic highlights key stages of the cell cycle, including the G1, S, G2, and M phases, as well as major proteins and protein complexes involved in regulating these processes, such as cyclins (*Cyc*), cyclin-dependent kinases (*CDK*), checkpoint proteins, and DNA repair proteins. Green indicates genes with decreased expression, whereas red indicates genes with increased expression.

**
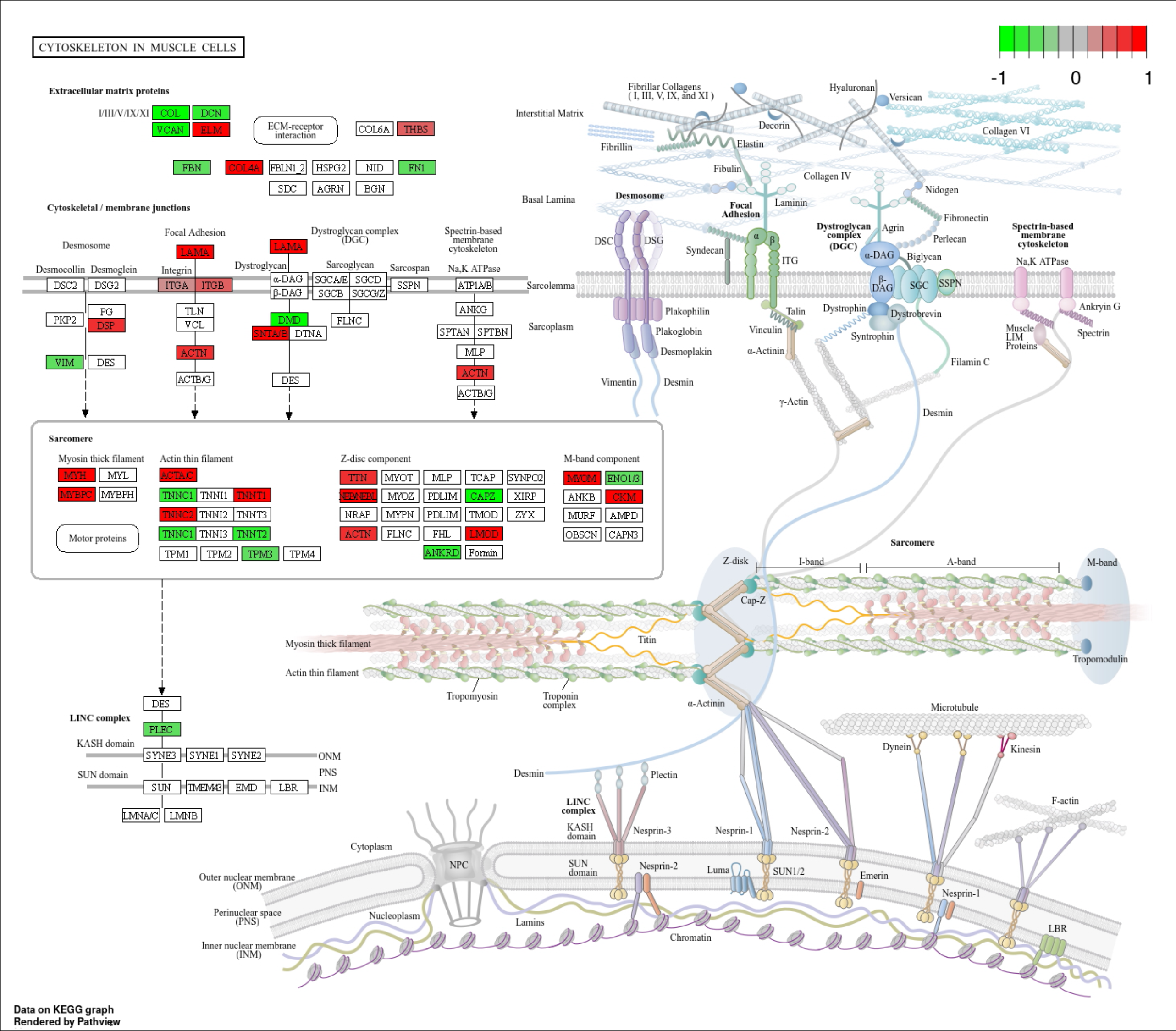
**

**Figure S18: Schematic of the cytoskeleton-related metabolic pathway in HSkM cells after the *DMD* and *UTRN* genes were silenced.**

The image illustrates the cytoskeleton-related metabolic pathway identified in the KEGG analysis (hsa04820). The schematic highlights key components of the cytoskeleton and extracellular matrix (ECM) interactions, including proteins such as collagens, dystroglycan complex, integrins, spectrin-based membrane skeleton, and sarcomere proteins. Green indicates genes with decreased expression, whereas red indicates genes with increased expression. Changes in the expression of cytoskeleton-related genes may lead to disruptions in muscle cell structure, stability, and function, which are significant in the context of DMD. The silencing of *DMD* and *UTRN* likely affects the integrity of the cytoskeleton and its interactions with the ECM, leading to increased susceptibility to mechanical damage and impaired muscle regeneration.

**
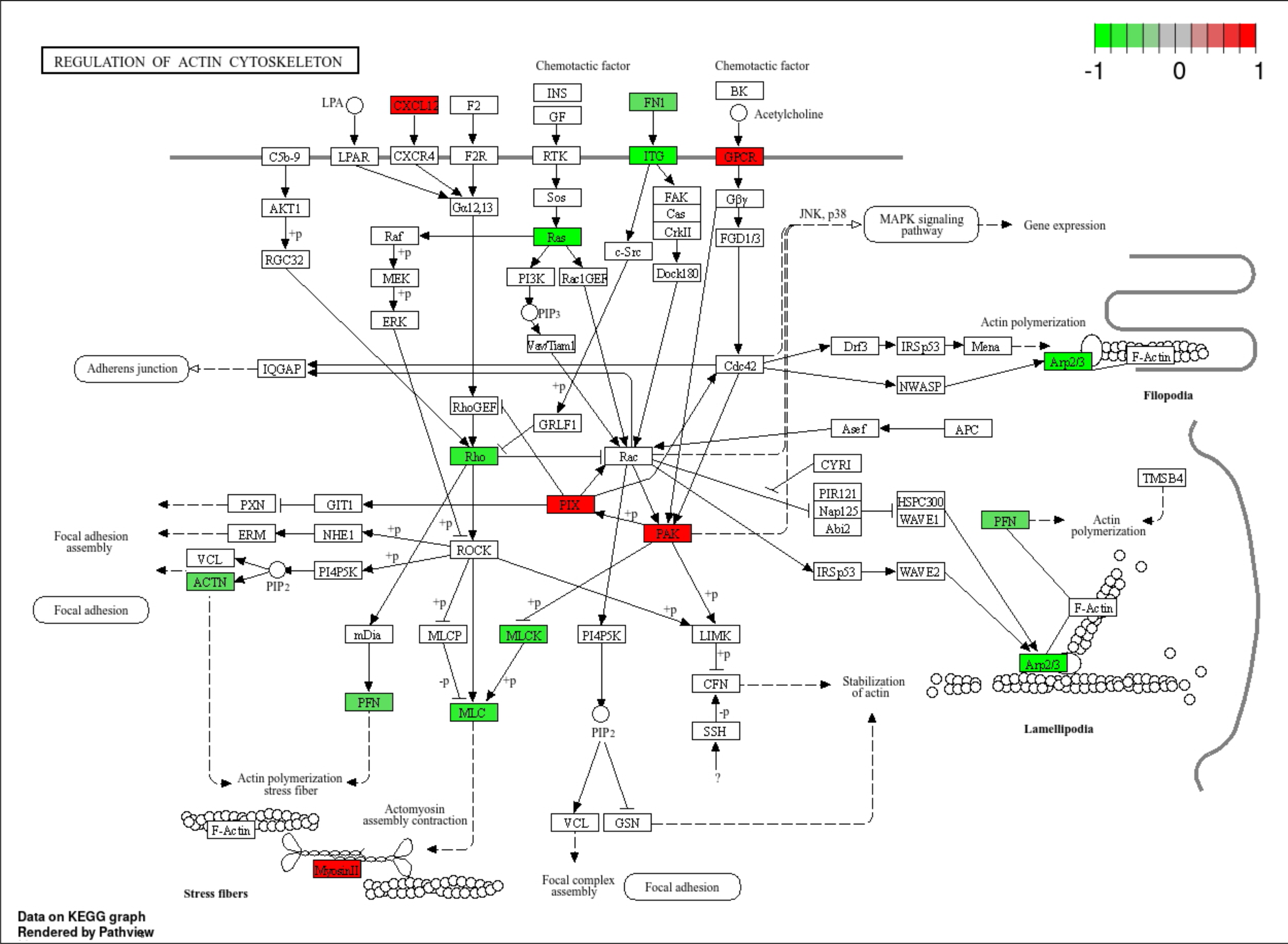
**

**Figure S19: Schematic of the regulation of the actin cytoskeleton pathway in HSkM cells after the *DMD* and *UTRN* genes were silenced.**

The image illustrates the regulation of actin cytoskeleton pathway identified in the KEGG analysis (hsa04810). Key components such as chemotactic factors, integrins, signalling molecules, and cytoskeletal proteins are highlighted. Green indicates genes with decreased expression, including *fibronectin* (*FN1*), *integrins* (*ITG*), *Ras*, *Rho*, *actinin* (*ACTN*), *PFN*, *MLC*, *MLCK*, and *Arp2/3*, suggesting a disruption in actin cytoskeleton organization, cell adhesion, and contractility. Red indicates genes with increased expression, such as *CXCL12*, *GPCR*, *PIX*, *PAK*, and *Myosin II*, which may reflect compensatory mechanisms aimed at enhancing cytoskeletal dynamics, cell signalling, and contractility.

**
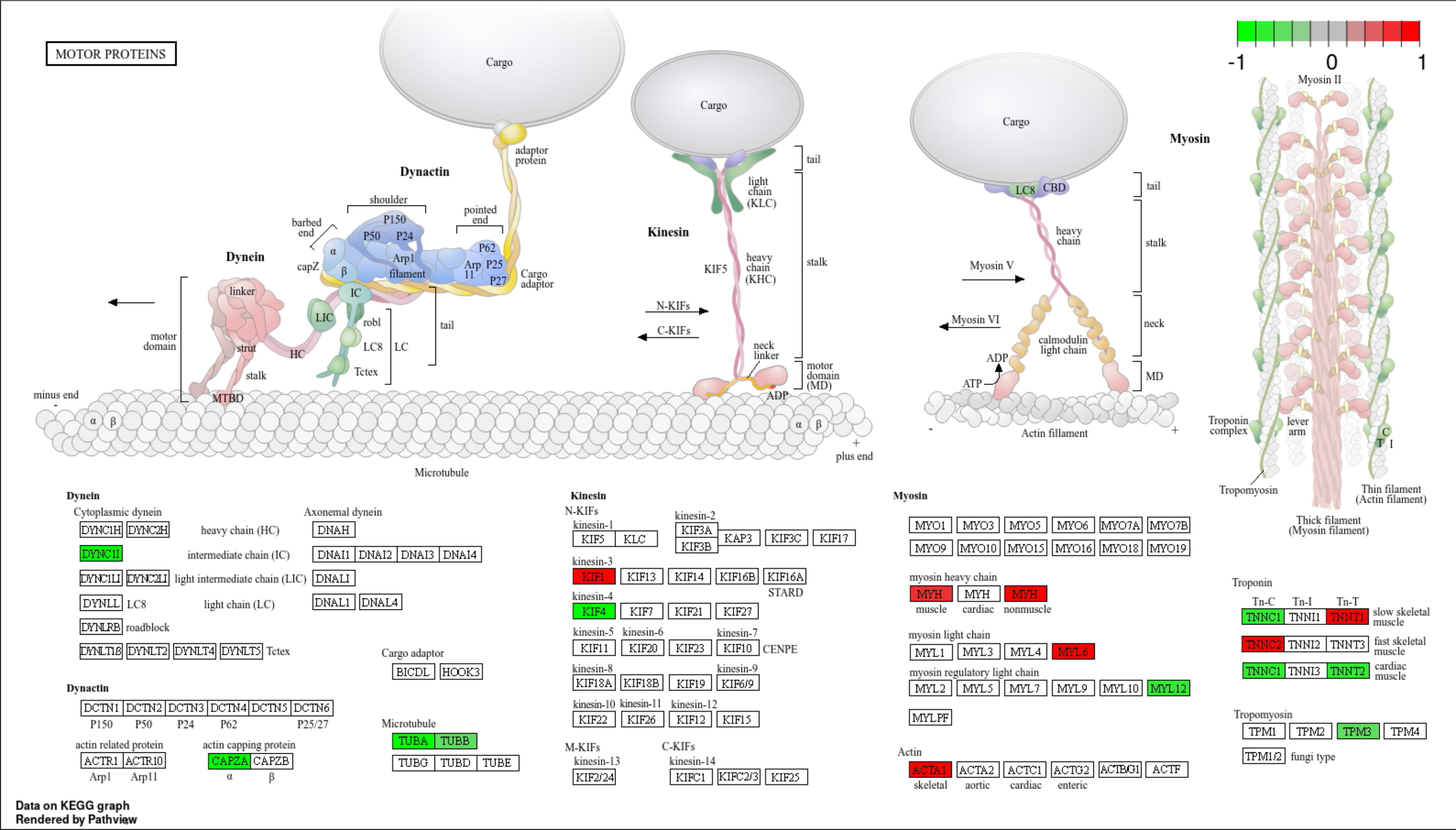
**

**Figure S20: Schematic of the motor protein pathway in HSkM cells after the *DMD* and *UTRN* genes were silenced.**

The image illustrates the motor protein pathway identified in the KEGG analysis (hsa04814). Key components such as myosin, kinesin, dynein, and associated regulatory proteins are highlighted. Green indicates genes with decreased expression, including those related to cytoskeletal dynamics, microtubule organization, and muscle contraction, suggesting a disruption in the structural and functional integrity of motor proteins. Red indicates genes with increased expression, such as those linked to motor protein activity and muscle contraction, which may reflect compensatory mechanisms aimed at enhancing cytoskeletal dynamics and contractility.

**
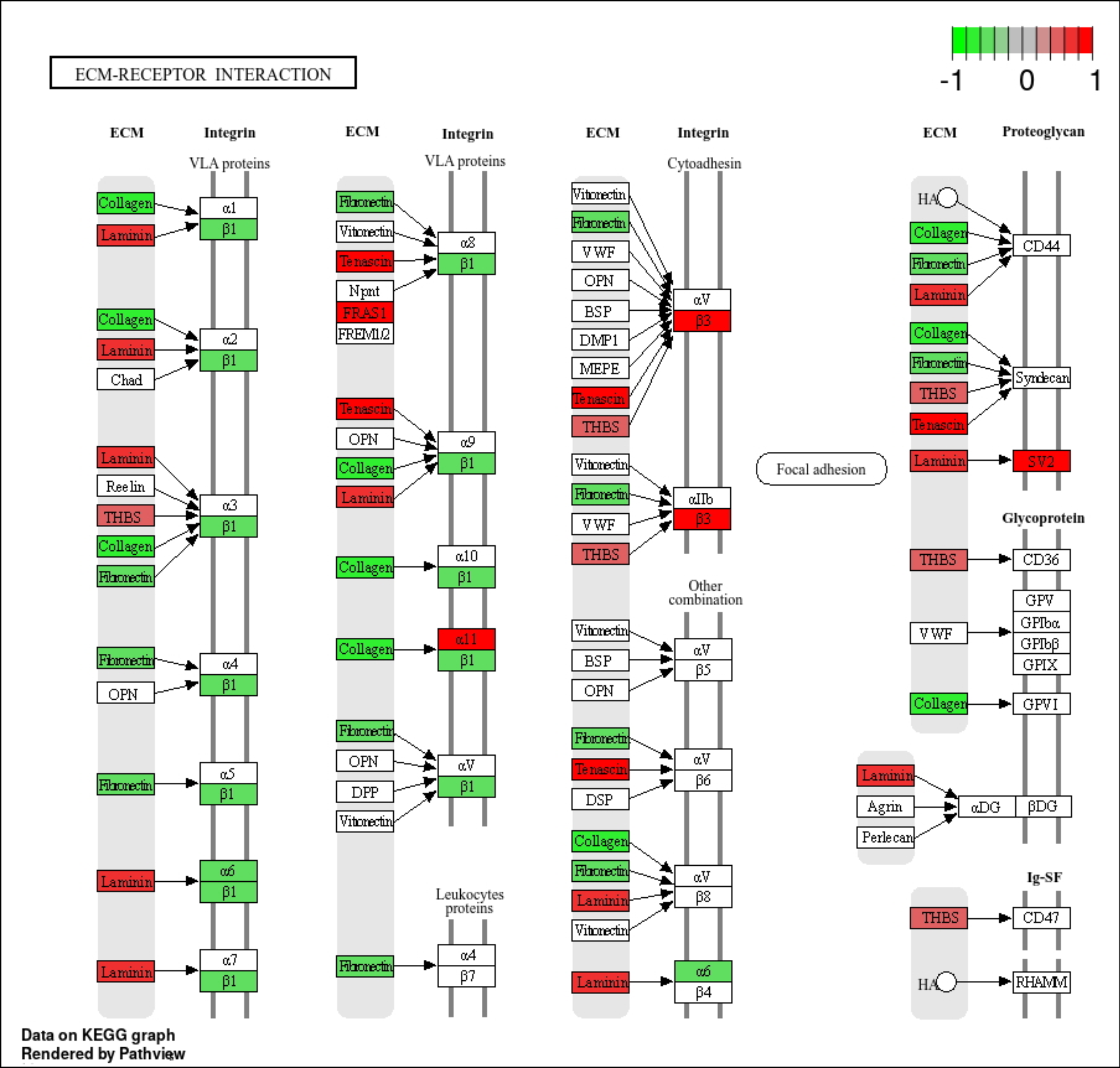
**

**Figure S21: Schematic of the ECM‒receptor interaction pathway in HSkM cells after the *DMD* and *UTRN* genes were silenced.**

The image illustrates the ECM‒receptor interaction pathway identified in the KEGG analysis (hsa04512). Key components such as integrins, collagens, laminins, and other extracellular matrix (ECM) proteins are highlighted. Green indicates genes with decreased expression, including collagen, iITGB1, ITGA6, and fibronectin, suggesting a disruption in ECM integrity and cell-matrix adhesion. Red indicates genes with increased expression, such as laminin, *THBS*, tenascin, integrin (*ITGA11*, *ITGB3*), and *SV2*, which may reflect compensatory mechanisms aimed at reinforcing ECM stability and enhancing cell adhesion and signalling pathways. Silencing of *DMD* and *UTRN* leads to decreased expression of key ECM and integrin genes, weakening the connection between the cytoskeleton and the ECM and increasing susceptibility to mechanical damage. Conversely, increased expression of certain genes may represent an adaptive response to stabilize the ECM and mitigate structural instability.

**
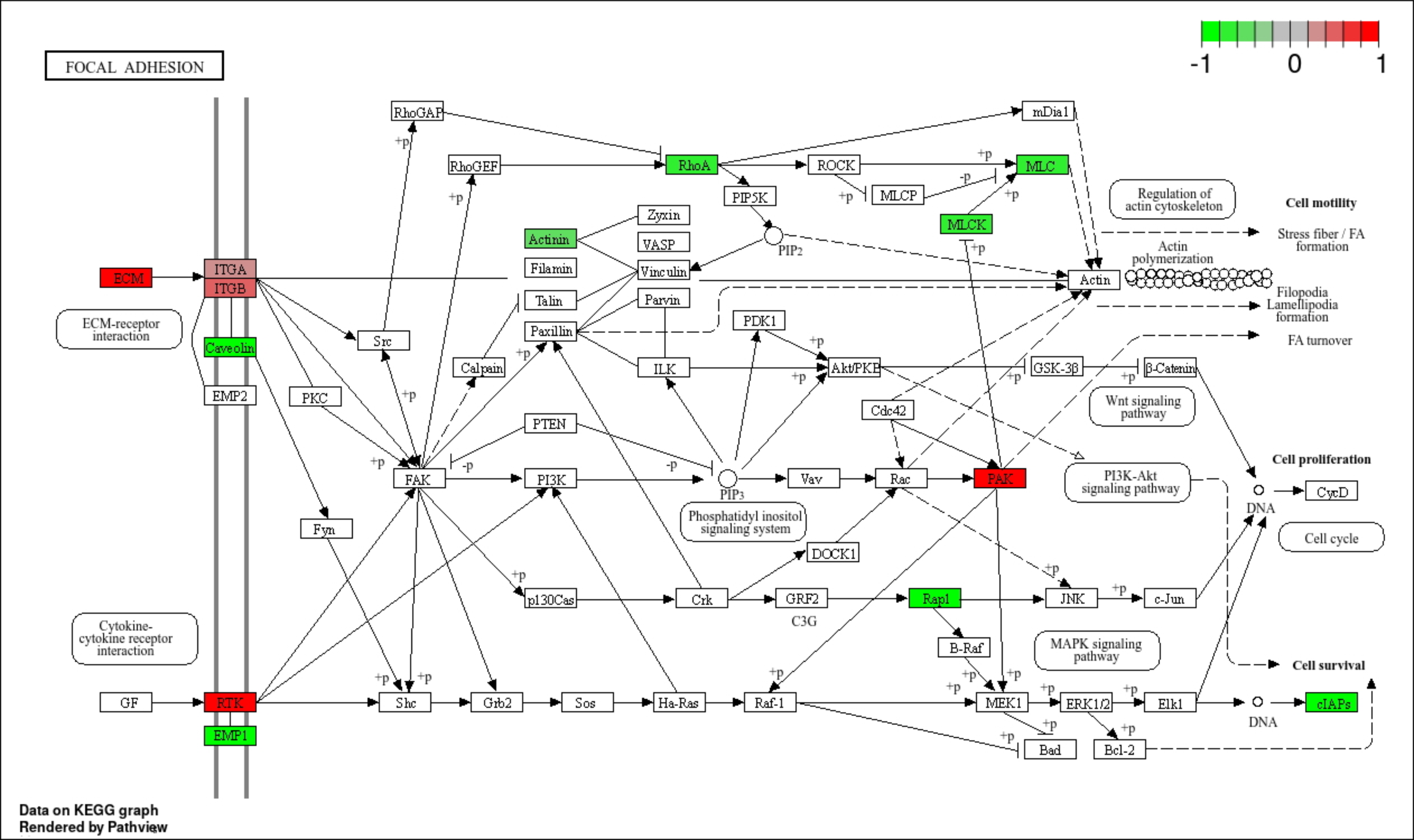
**

**Figure S22: Schematic of the focal adhesion pathway in HSkM cells after the *DMD* and *UTRN* genes were silenced.**

The image illustrates the focal adhesion pathway identified in the KEGG analysis (hsa04510). Key components such as integrins, cytoskeletal proteins, and signalling molecules are highlighted. Green indicates genes with decreased expression, including Caveolin, Actinin, *RhoA*, *MLC*, *MLCK*, *Rap1*, *EMP1*, and *cIAPs*, suggesting a disruption in cytoskeletal organization, cell contractility, and signal transduction. Red indicates genes with increased expression, such as ECM components, *ITGA*, *ITGB*, *RTK*, and *PAK*, which may reflect compensatory mechanisms aimed at enhancing cell-matrix adhesion, signal transduction, and cytoskeletal reorganization.

**
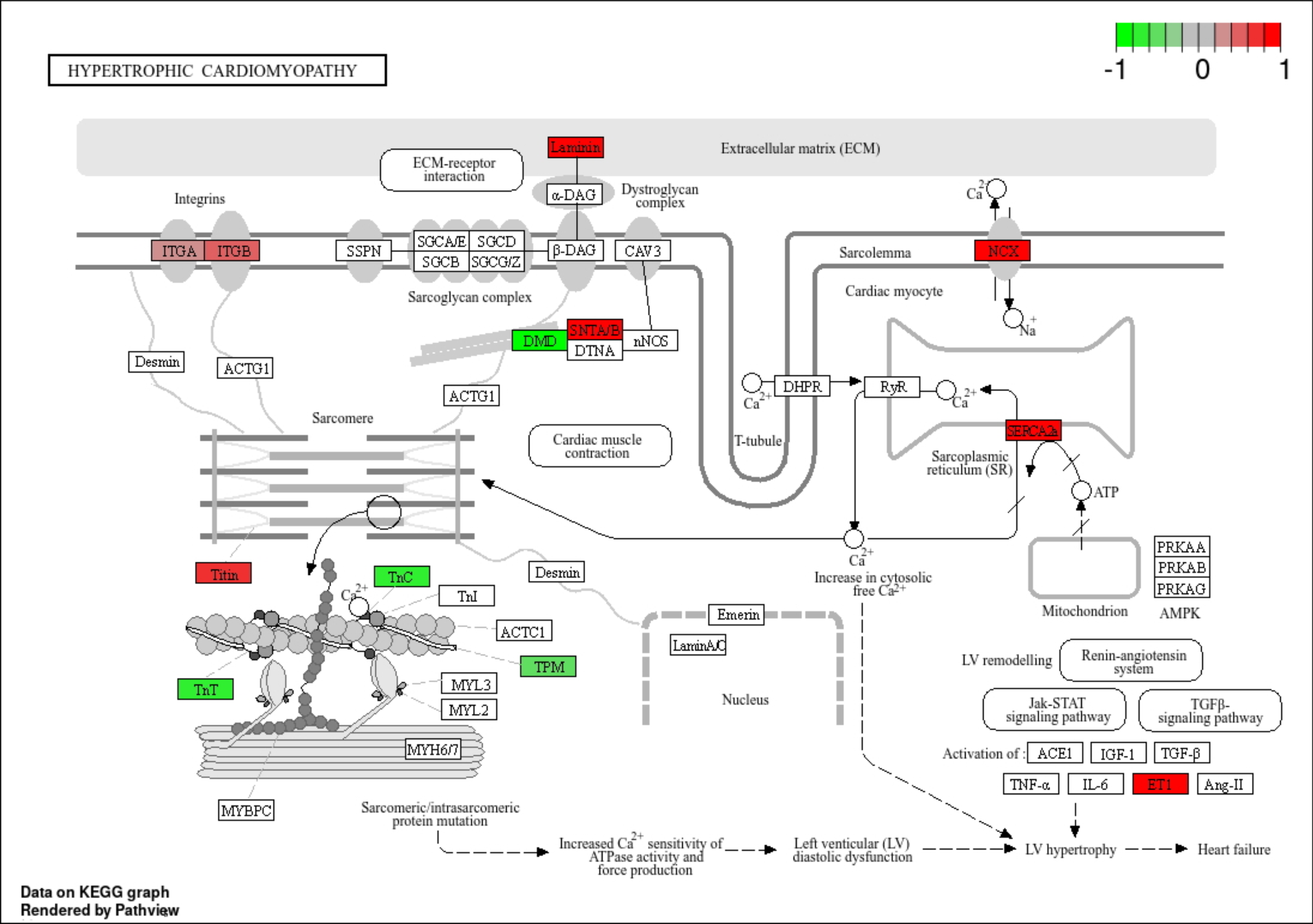
**

**Figure S23: Schematic of the hypertrophic cardiomyopathy pathway in HSkM cells after the *DMD* and *UTRN* genes were silenced.**

The image illustrates the hypertrophic cardiomyopathy pathway identified in the KEGG analysis (hsa05410). Green indicates genes with decreased expression, whereas red indicates genes with increased expression.

**
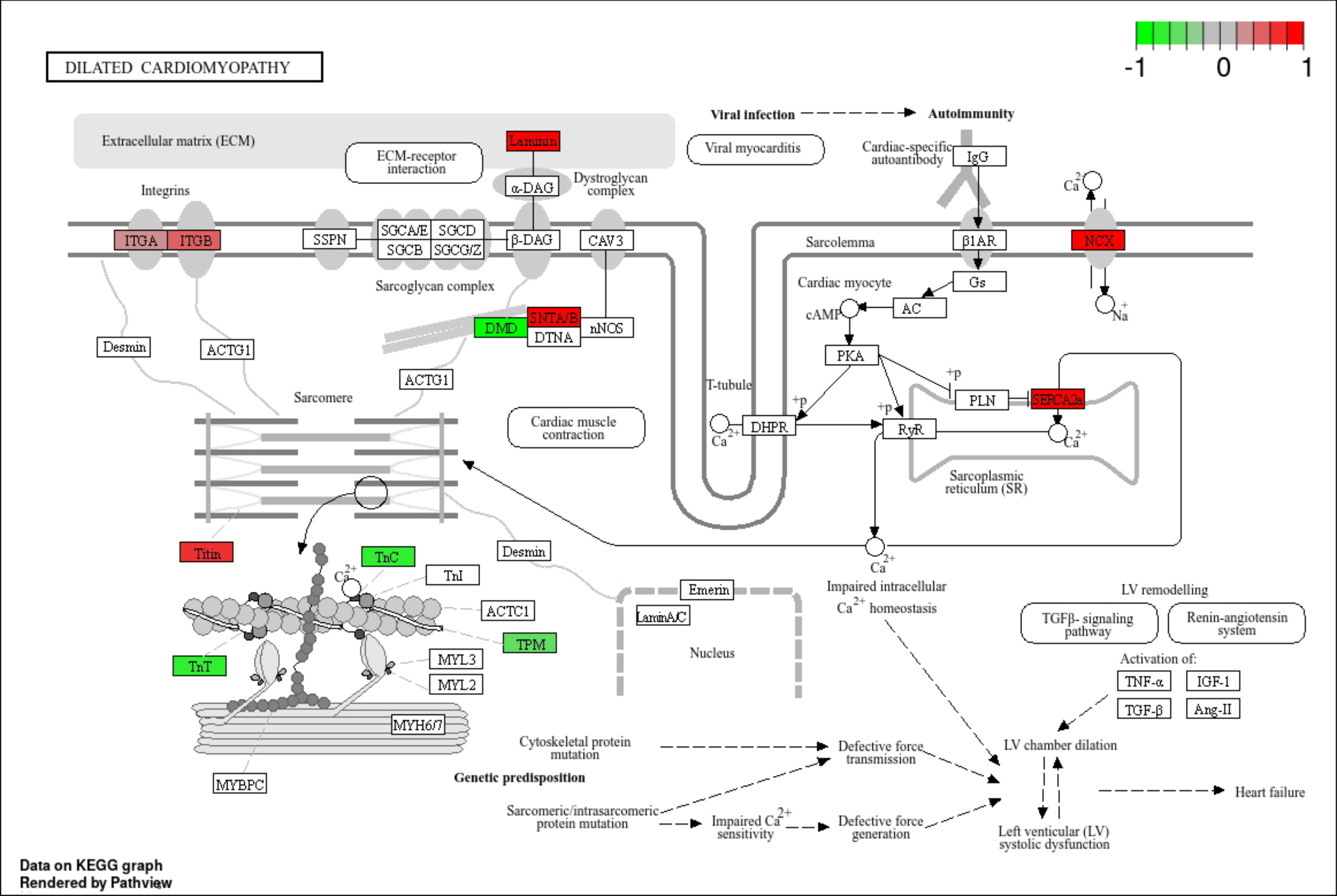
**

**Figure S24: Schematic of the dilated cardiomyopathy pathway in HSkM cells after the *DMD* and *UTRN* genes were silenced.**

The image illustrates the dilated cardiomyopathy pathway identified in the KEGG analysis (hsa05414). Green indicates genes with decreased expression, whereas red indicates genes with increased expression.

**
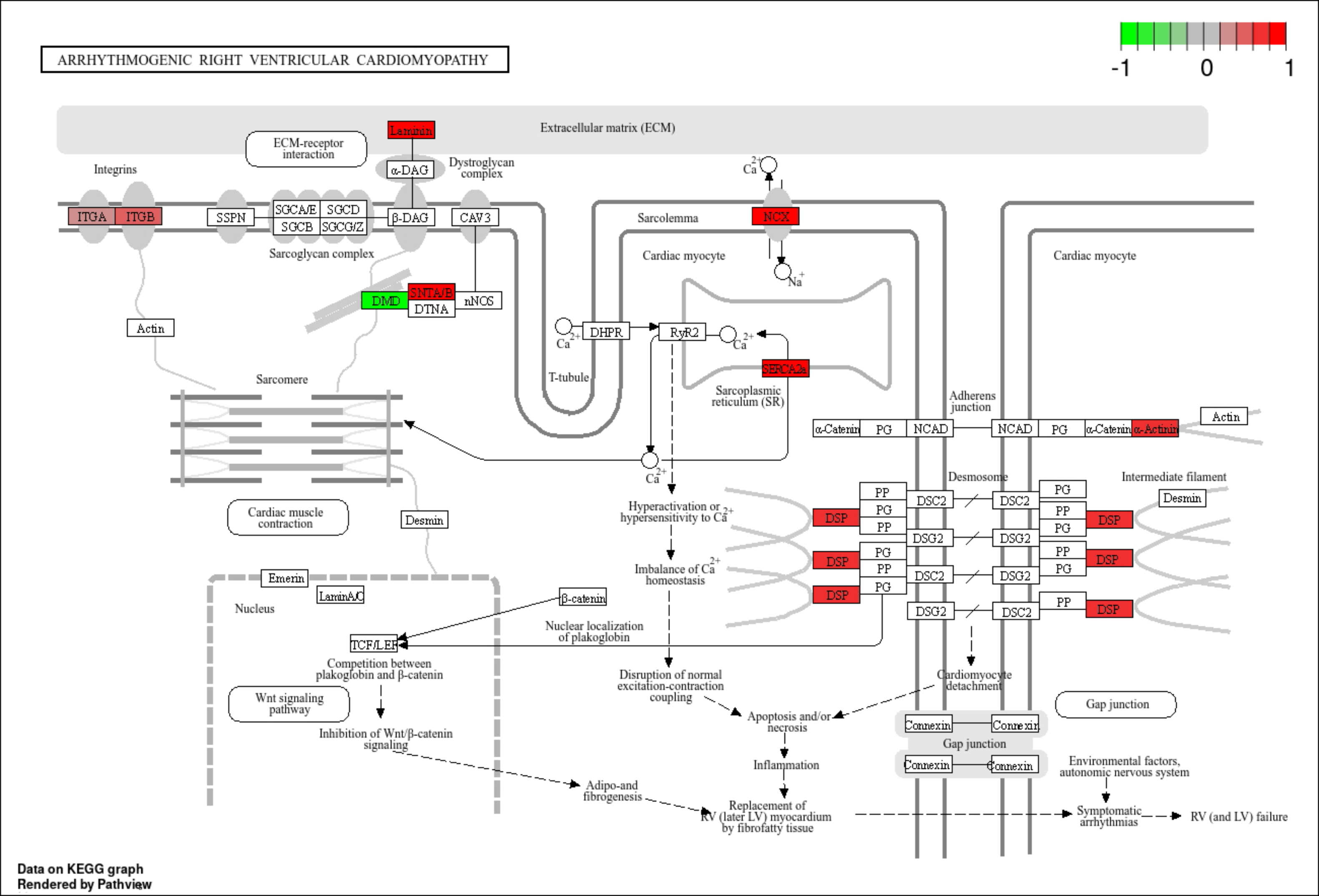
**

**Figure S25: Schematic of the arrhythmogenic right ventricular cardiomyopathy pathway in HSkM cells after the *DMD* and *UTRN* genes were silenced.**

The image illustrates the arrhythmogenic right ventricular cardiomyopathy pathway identified in the KEGG analysis (hsa05412). Green indicates genes with decreased expression, whereas red indicates genes with increased expression.
