# Supplementary material for: Dystrophins DP71 and DP427 determine cell viability during proliferation and myofibre differentiation
Source: Cell Death Dis. 2026 Apr 11;17(1):467. doi: 10.1038/s41419-026-08725-x (PMC13181019; doi:10.1038/s41419-026-08725-x)
Supplement: Supplementary file 3 — Original Data [file 41419_2026_8725_MOESM3_ESM.pdf]

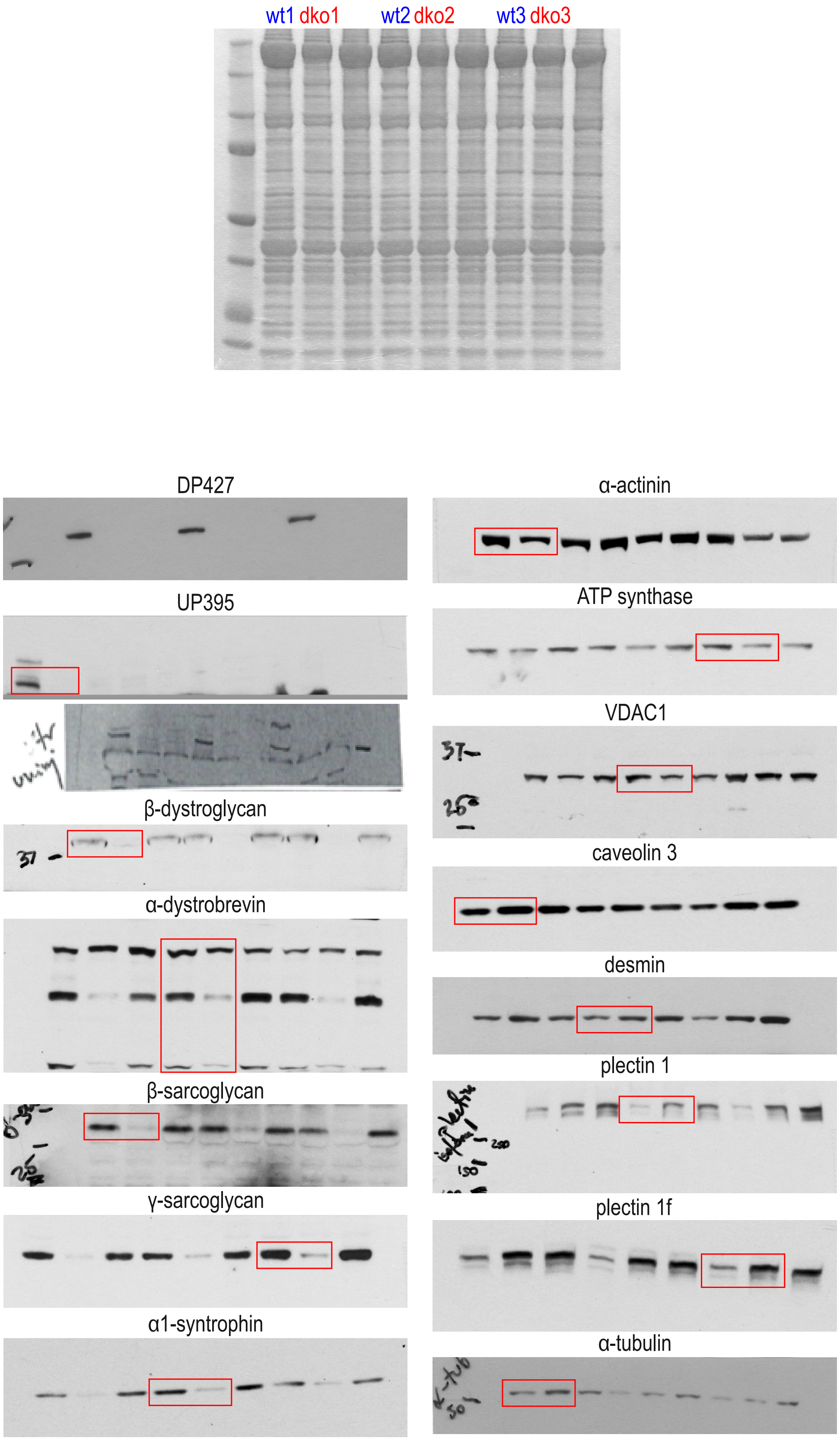
wt1 dko1
wt2 dko2
wt3 dko3
DP427
UP395
β-dystroglycan
α-dystrobrevin
β-sarcoglycan
γ-sarcoglycan
α1-syntrophin
α-actinin
ATP synthase
VDAC1
caveolin 3
desmin
plectin 1
plectin 1f
α-tubulin

Figure 1A

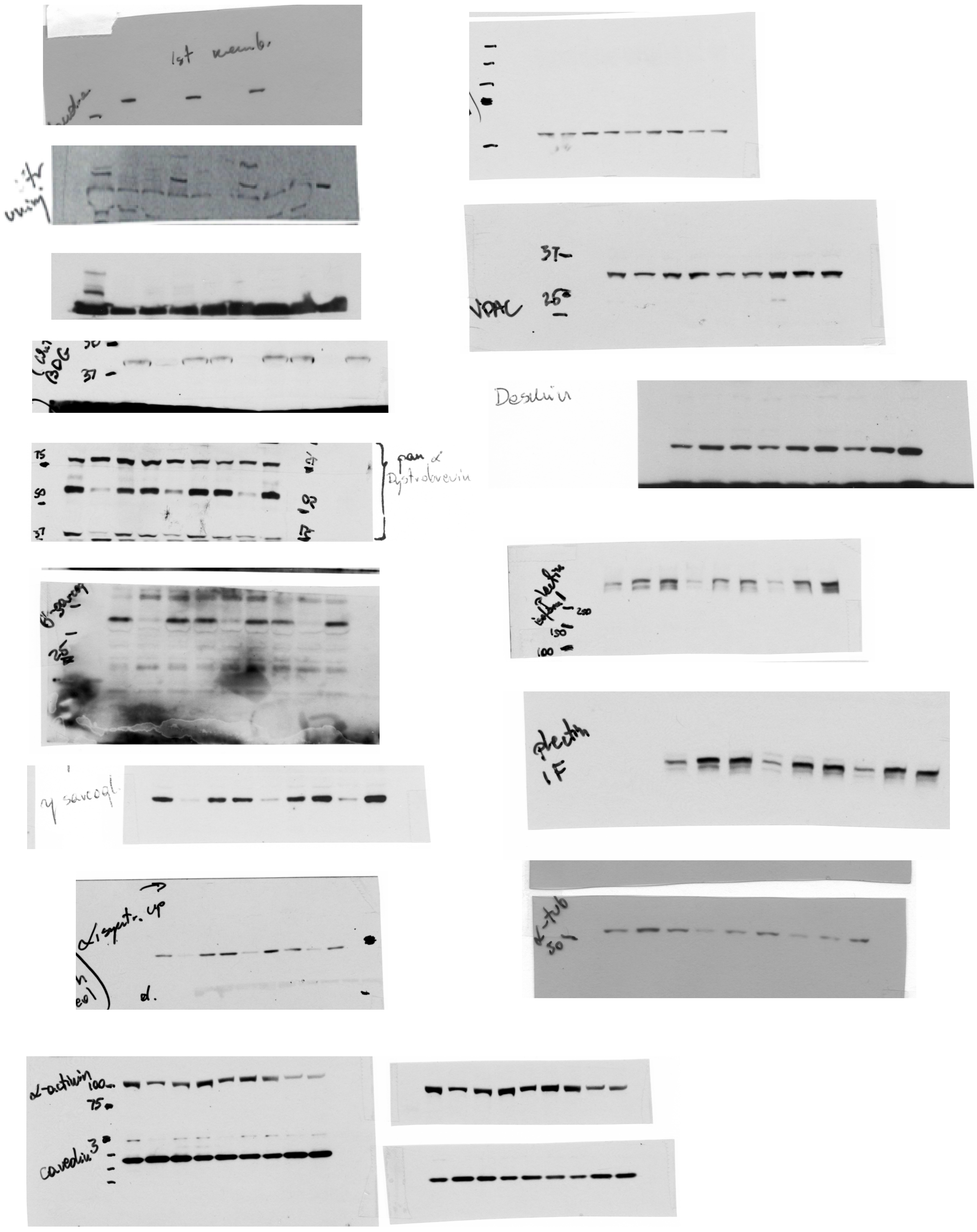

Figure 1A

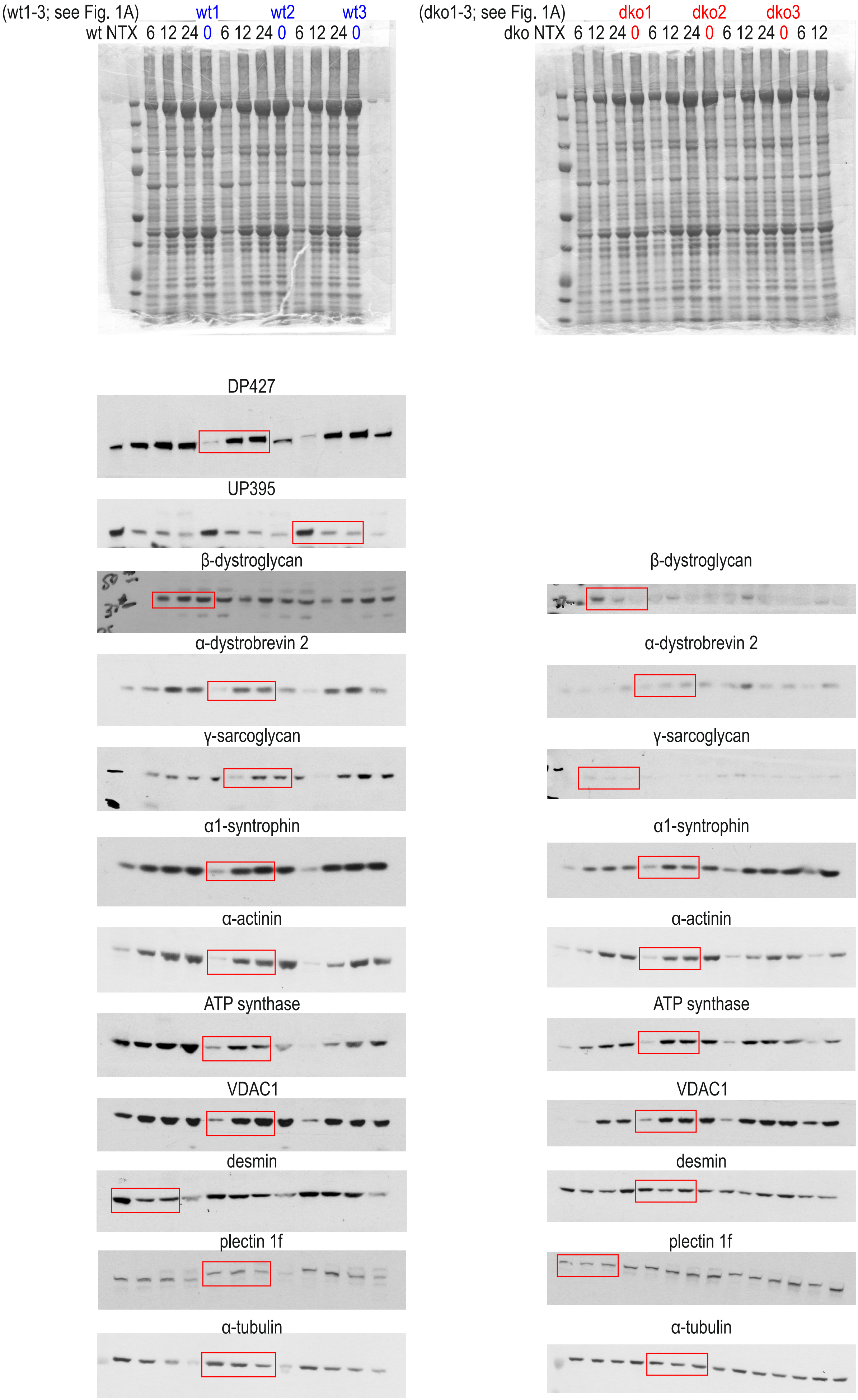
(wt1-3; see Fig. 1A)
wt1
wt2
wt3
wt NTX 6 12 24 0 6 12 24 0 6 12 24 0
(dko1-3; see Fig. 1A)
dko1
dko2
dko3
dko NTX 6 12 24 0 6 12 24 0 6 12 24 0 6 12
DP427
UP395
β-dystroglycan
α-dystrobrevin 2
γ-sarcoglycan
α1-syntrophin
α-actinin
ATP synthase
VDAC1
desmin
plectin 1f
α-tubulin

Figure 1C

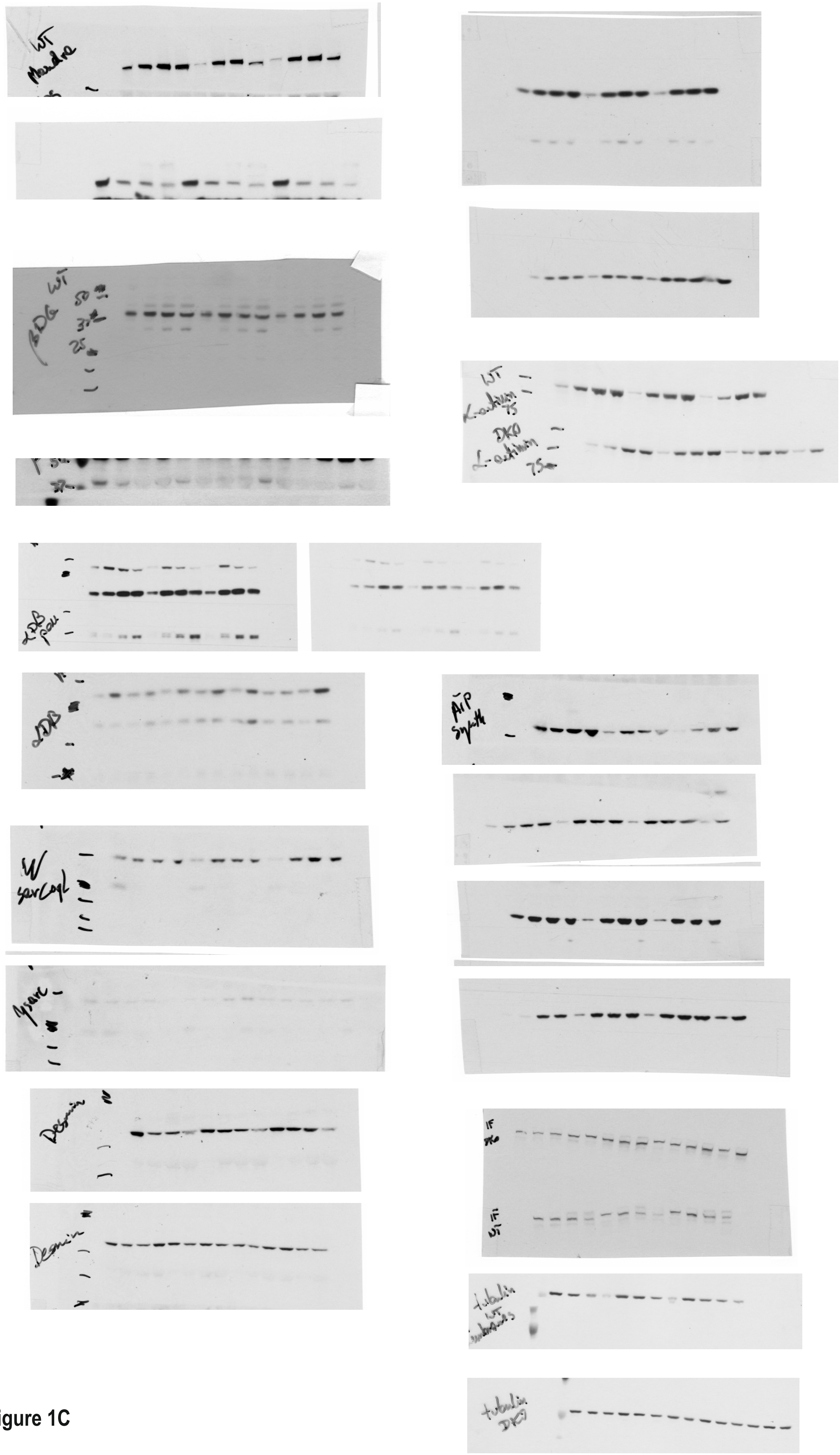
WT
WT
50
37
25
WT
75
DKO
75
ATP synth
Desmin
Desmin
IF DKO
IF WT
tubulin WT
tubulin DKO

Figure 1C

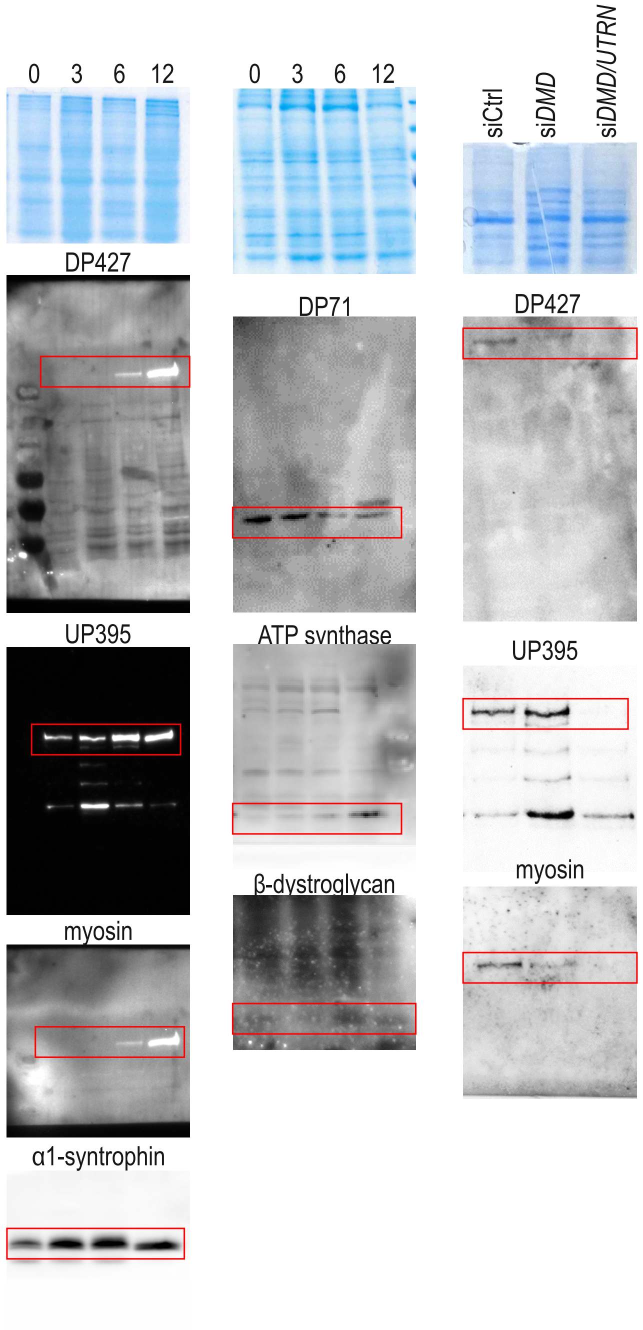

Figure 4C and 4E

d0 (proliferating myoblasts)

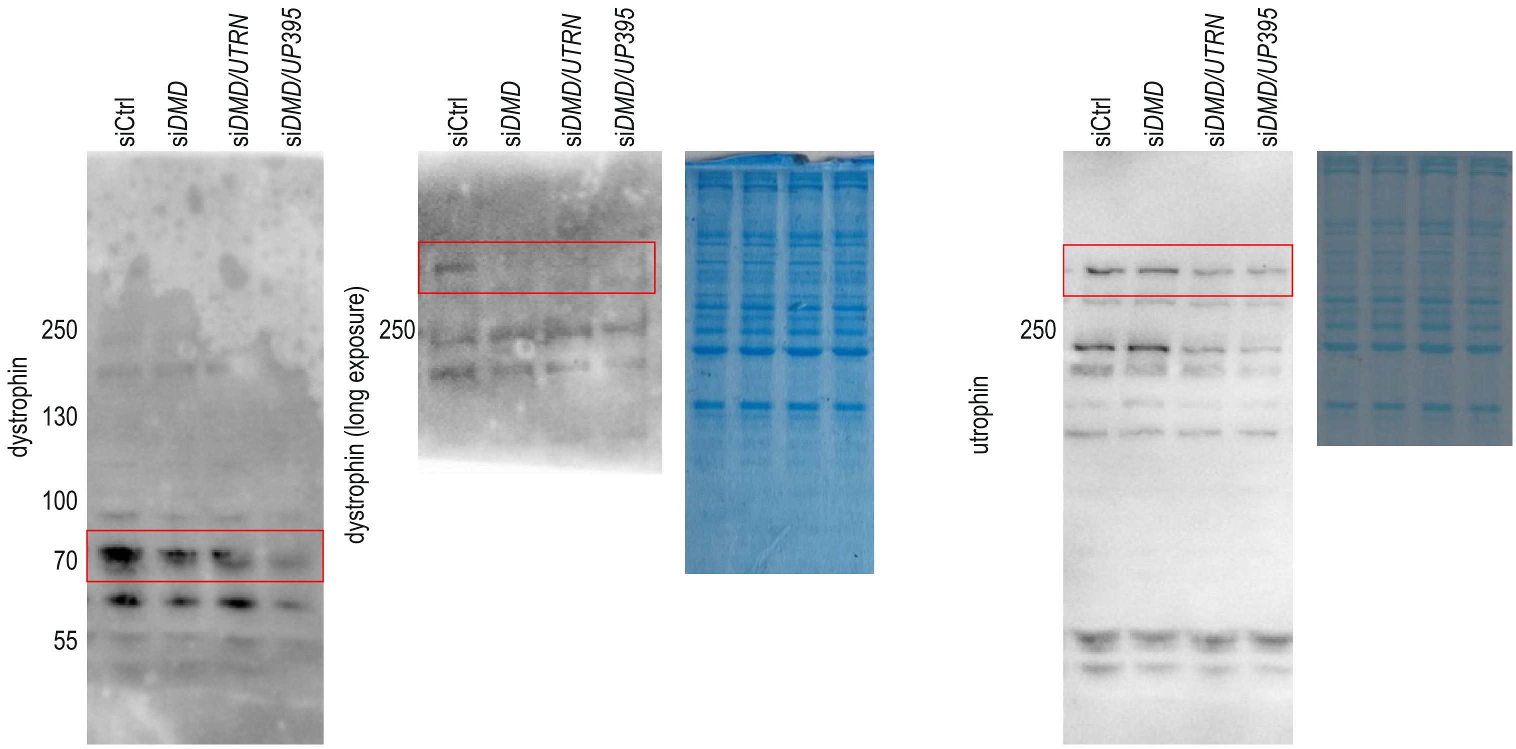

d12 (differentiated myotubes)

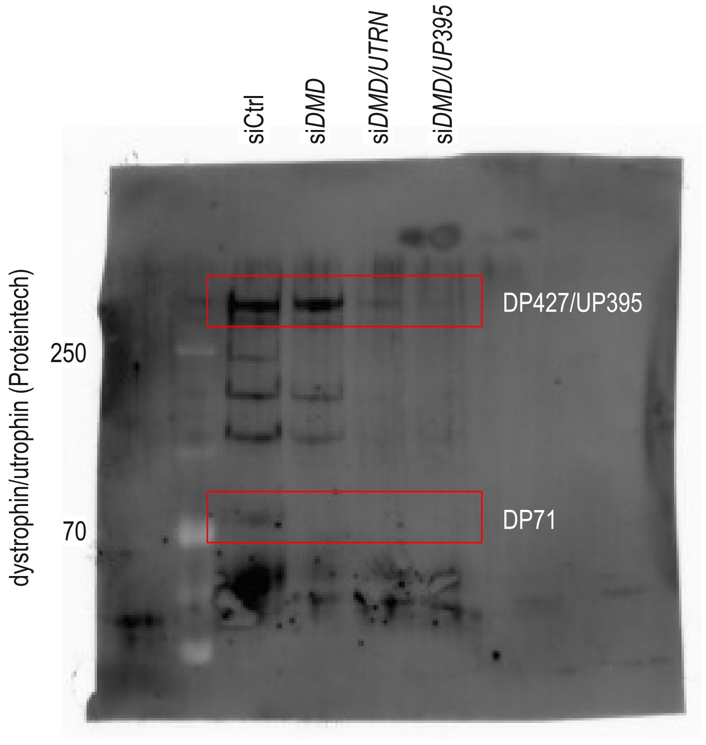

Figure 5B

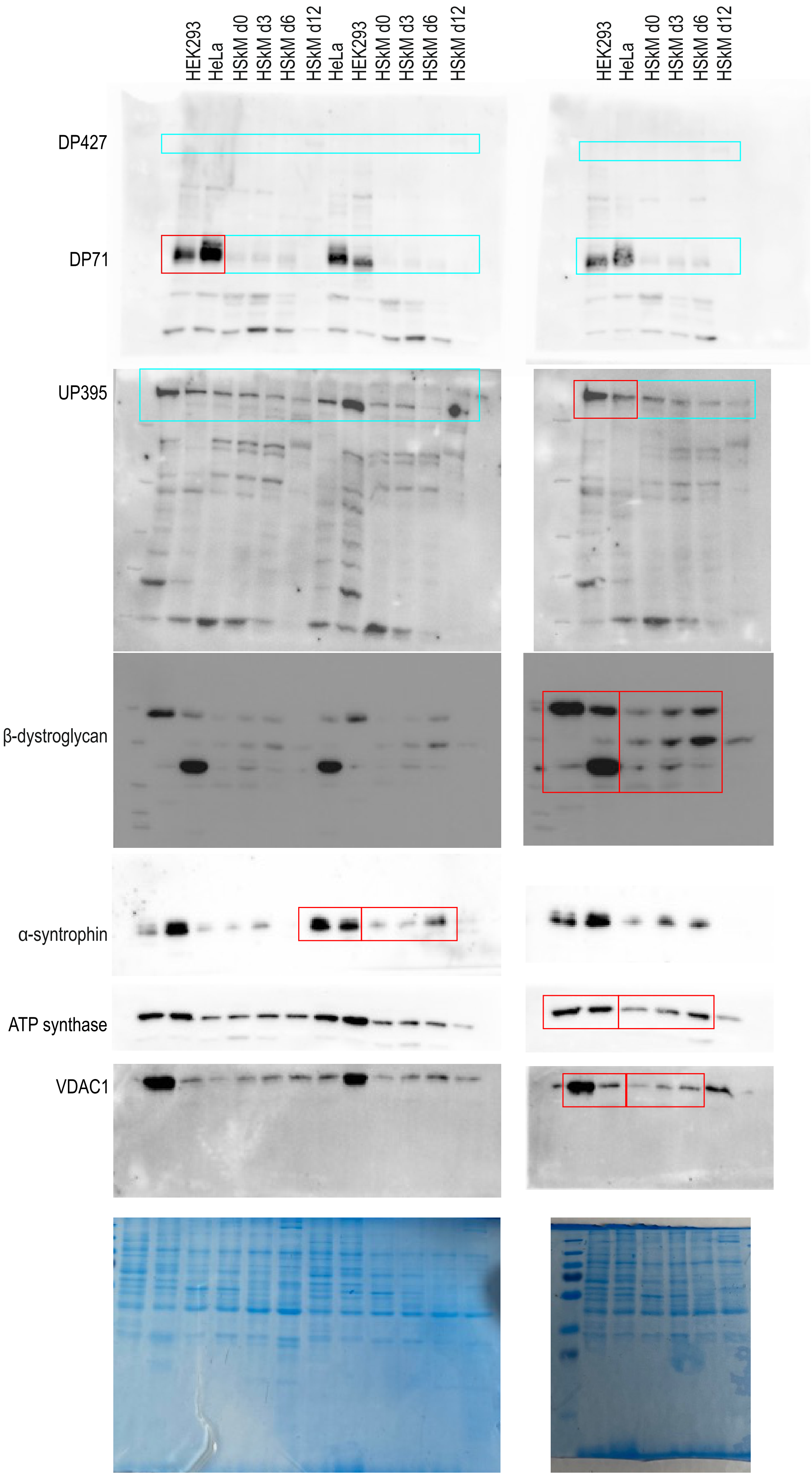

Figures 6A, S9C

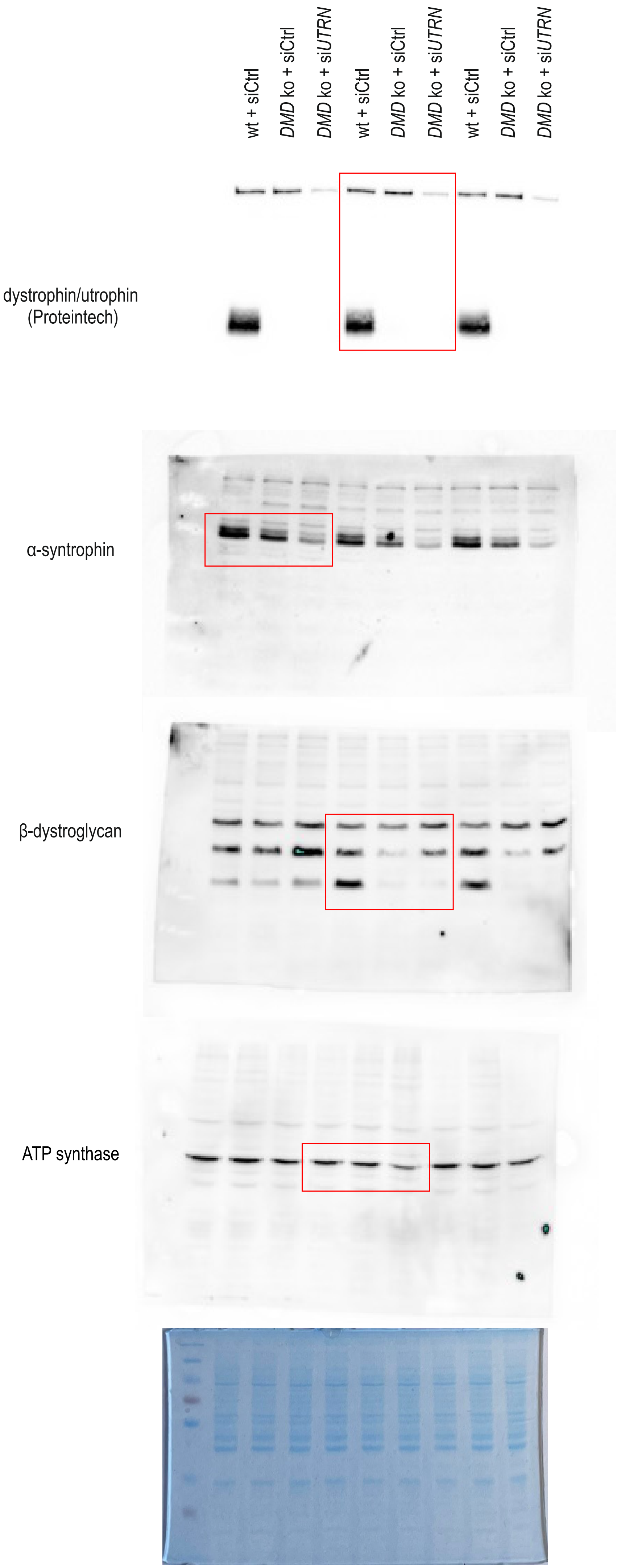

Figures 6C, S14B (HeLa)

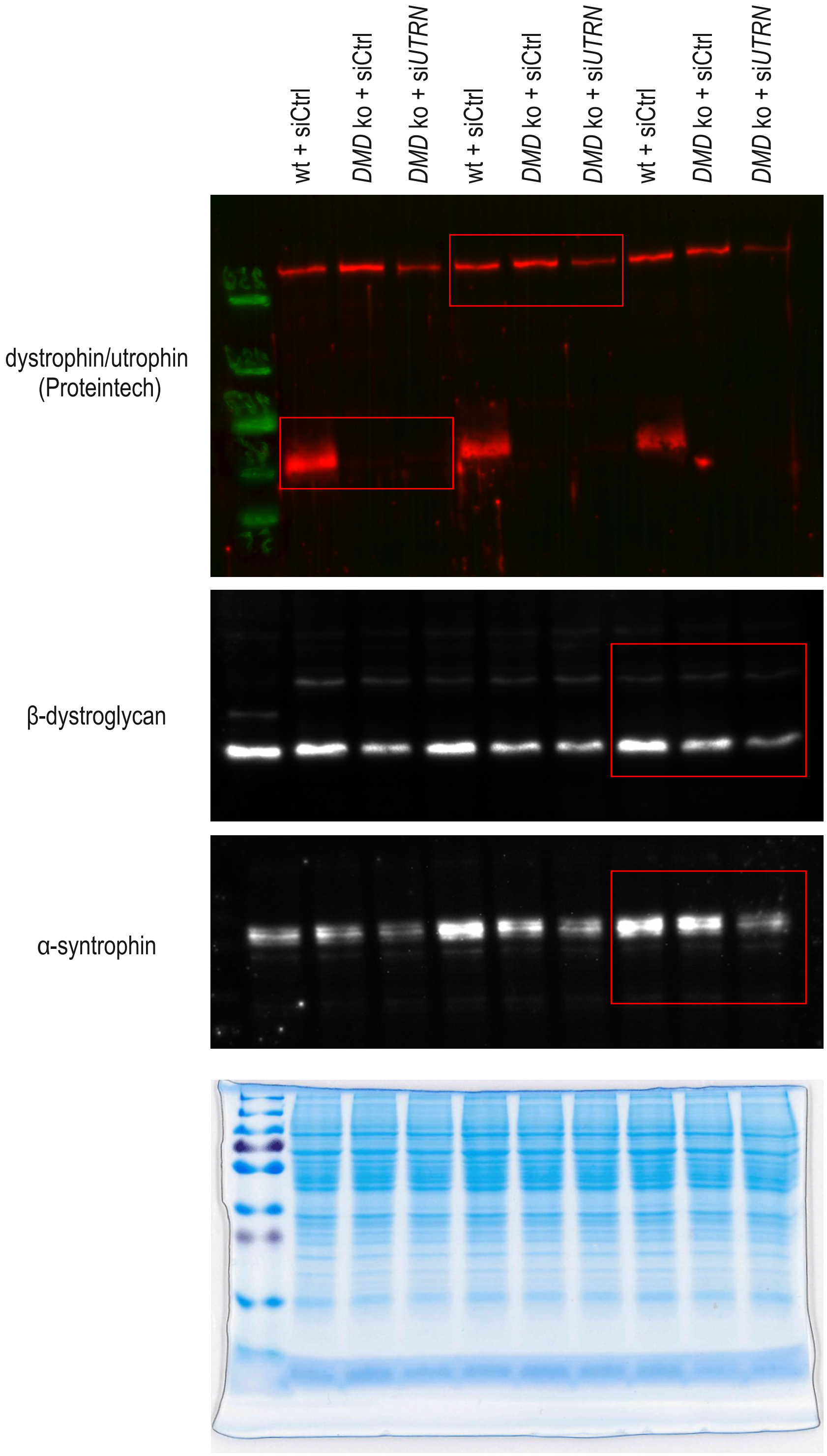

Figures 6C (HEK293)

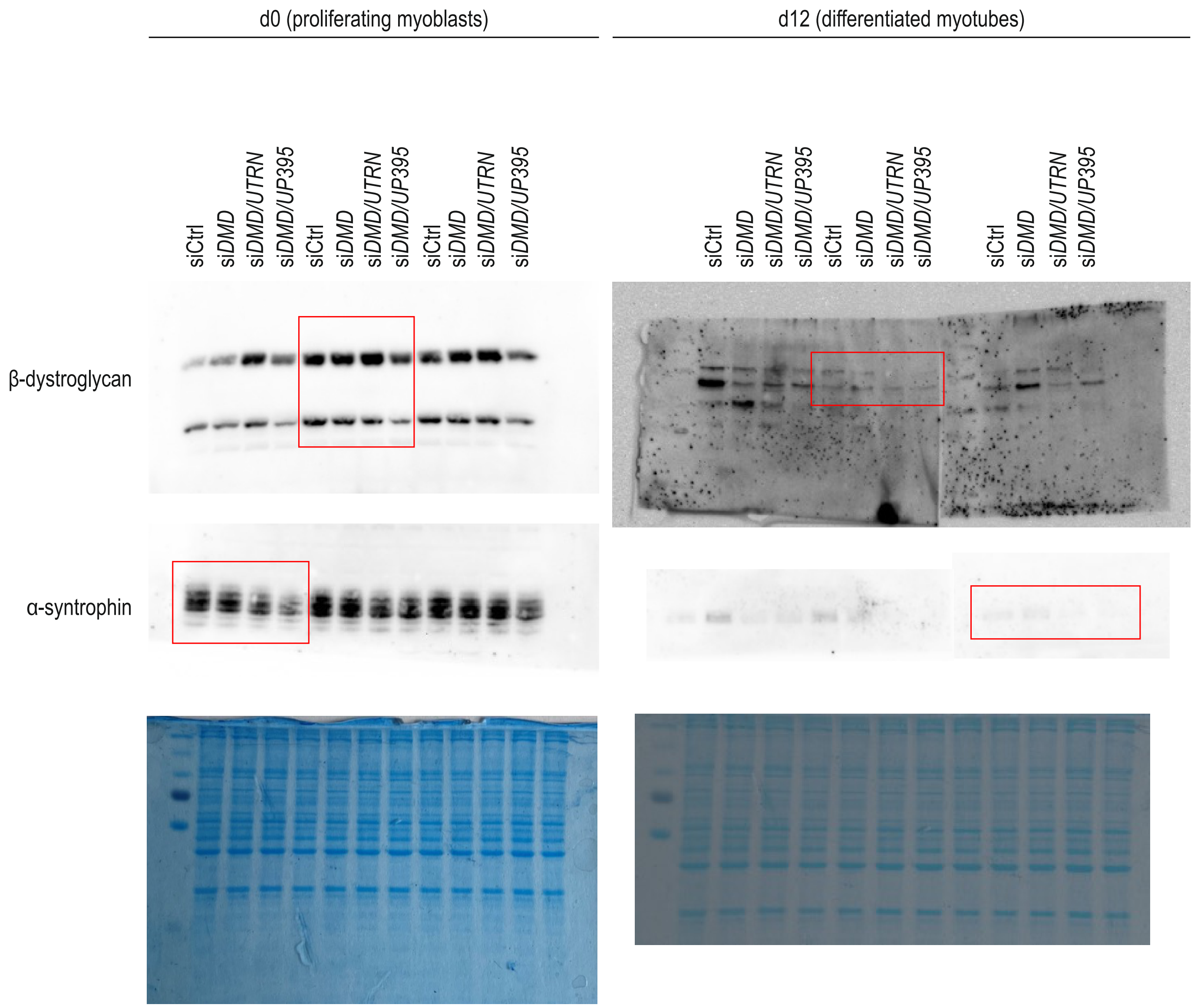
d0 (proliferating myoblasts)
d12 (differentiated myotubes)
siCtrl
siDMD
siDMD/UTRN
siDMD/UP395
siCtrl
siDMD
siDMD/UTRN
siDMD/UP395
siCtrl
siDMD
siDMD/UTRN
siDMD/UP395
siCtrl
siDMD
siDMD/UTRN
siDMD/UP395
siCtrl
siDMD
siDMD/UTRN
siDMD/UP395
siCtrl
siDMD
siDMD/UTRN
siDMD/UP395
β-dystroglycan
α-syntrophin

Figure S9D
